# Immune system-wide Mendelian randomization and triangulation analyses support autoimmunity as a modifiable component in dementia-causing diseases

**DOI:** 10.1038/s43587-022-00293-x

**Published:** 2022-10-14

**Authors:** Joni V. Lindbohm, Nina Mars, Pyry N. Sipilä, Archana Singh-Manoux, Heiko Runz, FinnGen, Gill Livingston, Sudha Seshadri, Ramnik Xavier, Aroon D. Hingorani, Samuli Ripatti, Mika Kivimäki

**Affiliations:** 1 Broad Institute of the Massachusetts Institute of Technology and Harvard University, The Klarman Cell Observatory, Cambridge, MA USA; 2 Department of Epidemiology and Public Health, University College London, London, UK; 3 Clinicum, Department of Public Health, University of Helsinki, Helsinki, Finland; 4 Institute for Molecular Medicine Finland, HiLIFE, University of Helsinki, Helsinki, Finland; 5 Université de Paris, Inserm U1153, Epidemiology of Ageing and Neurodegenerative diseases, Paris, France; 6 Research & Development, Biogen Inc., Cambridge, MA USA; 7 Division of Psychiatry, University College London, London, UK; 8 Camden and Islington NHS Foundation Trust, London, UK; 9 Glenn Biggs Institute of Alzheimer’s and Neurodegenerative Diseases, University of Texas Health Science Center, San Antonio, TX USA; 10 Boston University School of Public Health, Boston, MA USA; 11 New York University Grossman School of Medicine, New York, NY USA; 12 Boston University School of Medicine, Boston, MA USA; 13 Center for Computational and Integrative Biology, Massachusetts General Hospital and Harvard Medical School, Boston, MA USA; 14 Department of Molecular Biology, Massachusetts General Hospital and Harvard Medical School, Boston, MA USA; 15 Institute of Cardiovascular Science, University College London, London, UK; 16 University College London, British Heart Foundation Research Accelerator, London, UK; 17 Health Data Research UK, London, UK

**Keywords:** Ageing, Dementia, Autoimmune diseases

## Abstract

Immune system and blood–brain barrier dysfunction are implicated in the development of Alzheimer’s and other dementia-causing diseases, but their causal role remains unknown. We performed Mendelian randomization for 1,827 immune system- and blood–brain barrier-related biomarkers and identified 127 potential causal risk factors for dementia-causing diseases. Pathway analyses linked these biomarkers to amyloid-β, tau and α-synuclein pathways and to autoimmunity-related processes. A phenome-wide analysis using Mendelian randomization-based polygenic risk score in the FinnGen study (*n* = 339,233) for the biomarkers indicated shared genetic background for dementias and autoimmune diseases. This association was further supported by human leukocyte antigen analyses. In inverse-probability-weighted analyses that simulate randomized controlled drug trials in observational data, anti-inflammatory methotrexate treatment reduced the incidence of Alzheimer’s disease in high-risk individuals (hazard ratio compared with no treatment, 0.64, 95% confidence interval 0.49–0.88, *P* = 0.005). These converging results from different lines of human research suggest that autoimmunity is a modifiable component in dementia-causing diseases.

## Abstract (web summary)

Immune system dysfunction has been implicated in the development of dementias, but its causal role remains unknown. Providing converging results from different lines of human research, this study by Lindbohm et al. suggests that autoimmunity may be a modifiable component in diseases causing dementia.

## Main

Due to limited success in drug trials targeting the amyloid-β pathway, recent dementia research has explored alternative therapeutic targets from biomarkers linked to immune system dysfunction^1^. This new focus has been supported by epidemiological studies that have linked chronic inflammatory diseases (for example, diabetes, autoimmune diseases and severe infections) to increased risk of dementias^2–4^. In healthy state, the blood–brain barrier (BBB) protects the central nervous system (CNS) from peripheral neurotoxic molecules and pathogens, keeping the CNS immune privileged^2–4^. However, aging^5^ and peripheral inflammation that arises from low-grade systemic inflammation^6,7^ and infections^8^ can disrupt this function^9^. A dysfunctional BBB may promote expression of endothelial adhesion molecules and chemokines, leading to migration of peripheral leukocytes to the CNS^9^. These processes activate the central immune system and are hypothesized to expose the CNS to prolonged neuroinflammation and subsequent neurodegeneration^2^, which is supported by recent plasma proteomics studies^7,10^.

However, evidence on causal associations between a dysfunctional peripheral immune system, BBB and dementia-causing diseases remains limited. While some studies have observed that higher circulating C-reactive protein, IL-1, IL-6, tumor necrosis factor-α (TNF-α) and CD4 cell count may increase the risk of dementias^11–15^, these datasets are relatively small and captured only a limited number of biomarkers. A Mendelian randomization (MR) approach that uses large Genome-Wide Association Study (GWAS) libraries for unconfounded genetic proxies for biomarkers would allow a more comprehensive examination of the immune system and BBB biomarkers. This method enables the integrated use of data from multiple independent studies, testing of causality (although under strong assumptions), and has informed drug development^16^. As such, MR based on GWAS libraries is appealing in regard to explorative analyses of potential therapeutic targets for dementia-causing diseases. A complementary approach to improvement of reliability is triangulation, in which alternative methods, study designs and biomarkers with different sources of bias are used to test a common hypothesis^17^. If these converge, the results are more robust.

**Fig. 1:**
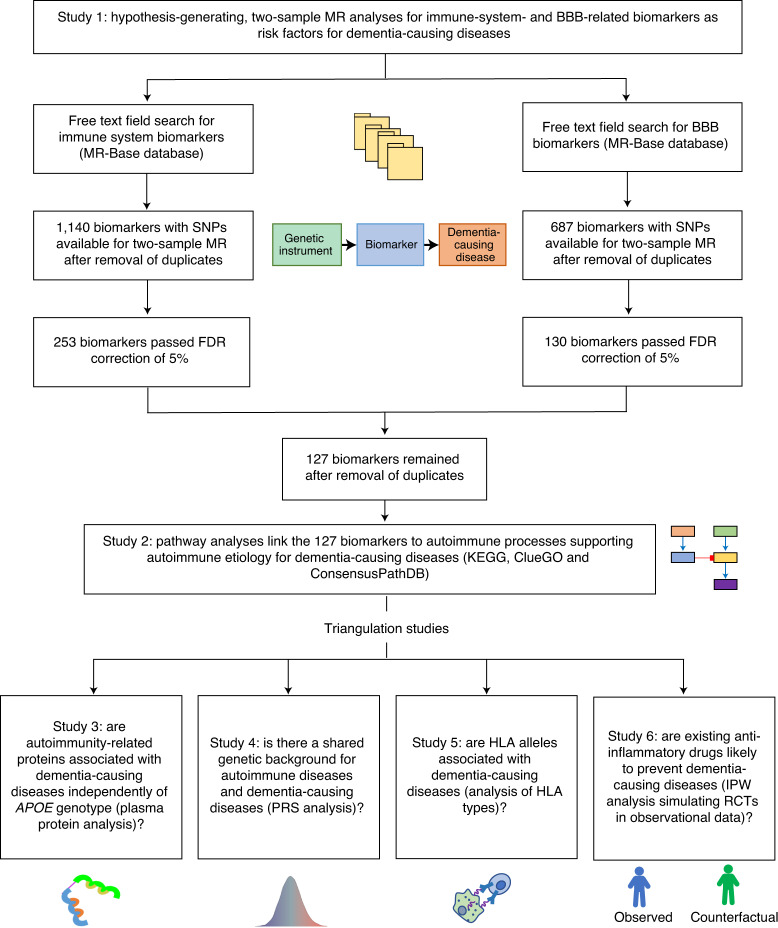
Design and rationale of six complementary studies. To study BBB- and immune system-related biology, biomarkers and drug targets for dementia-causing diseases, we conducted six separate studies. Study 1 used MR and MR-Base database to explore how BBB and immune system-related biomarkers associate with dementia-causing diseases . This hypothesis-generating study identified 127 biomarkers associated with dementia-causing diseases, many related to BBB, inflammation and self-tolerance, suggesting that inflammatory and autoimmune processes may play a role in these diseases. Study 2 is a pathway analysis on the associations of study 1. Providing additional support for the autoimmune hypothesis, the analysis showed that the biomarkers are enriched in several autoimmune-related biological processes and share pathways with amyloid-β, tau and α-synuclein proteins that characterize dementia-causing diseases. Study 3 examined the eight proteins that have protein quantitative loci near the *APOE* gene. In line with the autoimmune hypothesis, this study showed that IFIT2, an anti-inflammatory protein, decreases risk for dementia-causing diseases independent of *APOE*. Study 4 examined which diseases are associated with a polygenic risk score constructed from SNPs associated with the 127 biomarkers. Using phenome-wide analysis, this study showed that several autoimmune diseases, especially type 1 diabetes and rheumatic arthritis, share a genetic background with dementia-causing diseases. Study 5 provided further support for the autoimmune hypothesis by identifying nine HLA alleles associated with dementia-causing diseases. Study 6 used IPW analyses to simulate randomized control trials in observational data. It examined whether the autoimmune component is modifiable with anti-inflammatory medication. These analyses showed that methotrexate and TNF-α inhibitors may be preventative medications for dementia-causing diseases.

Here, we combine six studies using MR and triangulation to gain new insights into dementia etiology and to identify drug targets and anti-inflammatory medications for repurposing for dementia-causing diseases (Fig. 1 and Table 1). In the first study (study 1), we perform MR analyses based on GWAS libraries for a total of 1,827 peripheral immune system and BBB biomarkers to identify causal associations with dementia-causing diseases (including Alzheimer’s and Parkinson’s disease, vascular dementia, frontotemporal dementia and cognitive performance). The findings suggest that autoimmune biomarkers may play an important role in disease etiology. The second study (study 2) uses pathway analyses to identify biological processes in which these biomarkers are enriched and provides additional support for the autoimmune hypothesis. Studies 3–6 lend further, consistent support for the autoimmune hypothesis from four analyses that are independent of studies 1 and 2: plasma proteomics, polygenic risk scores, human leukocyte antigen (HLA) allele analyses and inverse-probability-weighted (IPW) survival analysis that simulates a randomized controlled trial (RCT) design using observational data. We identify several potential new drug targets for dementia-causing diseases and provide evidence of a shared genetic background between dementia-causing and autoimmune diseases. Based on the different lines of research from the six studies, we propose that dementia-causing diseases may have an inflammatory autoimmune component that is modifiable with currently available anti-inflammatory medications and new therapeutics targeting the identified biomarkers.

**Table 1:** Characteristics of the main cohorts used in the six studies

Cohorts used	Baseline characteristics	Exposure measurement	Outcome measurement
**Study 1: MR**			
**Exposures**			
UK Biobank, UK BiLEVE, INTERVAL	173,480 participants; mean age 54 years; 48% men	Blood cells including leukocytes, erythrocytes and platelets	–
SardiNIA dataset, Italy	3,757 participants; mean age 45 years; 43% men	Flow cytometry of detailed leukocyte types	–
INTERVAL study, UK	3,301 participants; mean age 44 years; 51% men	Plasma proteins measured with SomaScan v.3	–
KORA F4 study, Germany	3,080 participants; mean age 56 years; 49% men	Plasma proteins measured with SomaScan v.3	–
**Outcomes**			
FinnGen, Finland	339,233 participants; mean age 54 years; 44% men	–	Alzheimer’s disease and subtypes, Parkinson’s disease, frontotemporal dementia, vascular dementia, dementia outcomes from national hospital discharge (available from 1968), death (from 1969), cancer (from 1953) and medication reimbursement (from 1964) and purchase (from 1995) registries
IGAP (several cohorts)	54,162 participants; mean age 71 years; 41% men	–	Late-onset Alzheimer’s disease from hospital discharge, death, autopsy and medication reimbursement registries
Multicohort study	1,131,881 participants; mean age 61 years; 46% men	–	Cognitive performance measured using immediate word recall task, a delayed word recall task, a naming task and a counting task, Henmon–Nelson test of mental ability, overall GPA, math, science and verbal GPA and educational attainment
ADGC, EADI, CHARGE, GERAD/PERADES consortium	63,926 participants; mean age 73 years; 41% men	–	Late-onset Alzheimer’s disease from hospital discharge, death, autopsy and medication reimbursement registries
IPDGC consortium	1,474,097 participants; mean age 57 years; 45% men	–	Parkinson’s disease from hospital discharge, death, autopsy and medication reimbursement registries
**Study 2: pathway analyses**			
KEGG, ConsensusPathDB databases	ConsensusPathDB-human integrates interaction networks in humans including 31 public databases; KEGG pathways are a collection of manually drawn pathway maps of known molecular interactions	Available UniProt IDs for the 127 biomarkers	Interaction path to amyloid precursor protein, tau protein or α-synuclein that characterize Alzheimer’s and Parkinson’s disease
**Study 3: plasma proteomics**			
Whitehall II, UK	6,545 participants; mean age 56 years; 71% men	Plasma proteins measured with SomaScan v.4	National Health Services Hospital Episode Statistics database, the British National mortality register and 5-yearly clinical screening
**Study 4: PRS**			
FinnGen, Finland	339,233 participants; mean age 54 years; 44% men	Illumina and Affymetrix arrays; AxiomGT1 algorithm for Affymetrix data; imputation with population-specific SISu v.3	National hospital discharge (from 1968), death (from 1969), cancer (from 1953) and medication reimbursement (from 1964) and purchase (from 1995) registries
**Study 5: HLA analyses**			
FinnGen, Finland	339,233 participants; mean age 54 years; 44% men	rSSO-Luminex technology (Labtype, One Lambda); PCR–SSP (Micro SSP Generic HLA Class I/II DNA Typing Trays, One Lambda; Olerup SSP genotyping; AlleleSEQR PCR/Sequencing kits, Atria Genetics; BI 3130xl genetic analyzer (Applied Biosystems, Thermo Fisher Scientific); Immunochip array (Illumina); imputation HLA*IMP:0240 (The Oxford HLA Imputation Framework)	National hospital discharge (from 1968), death (from 1969), cancer (from 1953) and medication reimbursement (from 1964) and purchase (from 1995) registries
**Cohort in study 6: IPW analyses**			
FinnGen, Finland	117,773 participants; mean age 55 years; 55% men	ATC codes from medication reimbursement (1997–2019) and purchase (1997–2019) registries Illumina and Affymetrix arrays; AxiomGT1 algorithm for Affymetrix data; imputation with population-specific SISu v.3	National hospital discharge (available from 1968), death (from 1969), cancer (from 1953) and medication reimbursement (from 1964) and purchase (from 1995) registries

For consortium and multicohort studies, mean age and proportion of men are reported for each cohort. ADGC, Alzheimer Disease Genetics Consortium; CHARGE, Cohorts for Heart and Aging Research in Genomic Epidemiology Consortium; EADI, European Alzheimer’s Disease Initiative; GERAD/PERADES, Genetic and Environmental Risk in AD/Defining Genetic, Polygenic and Environmental Risk for Alzheimer’s Disease Consortium; GPA, grade point average; IGAP, International Genomics of Alzheimer’s Project; IPDGC, International Parkinson Disease Genomics Consortium; KORA Kooperative Gesundheitsforschung in der Region Augsburg

## Results

### MR and plasma protein analyses

**Fig. 2:**
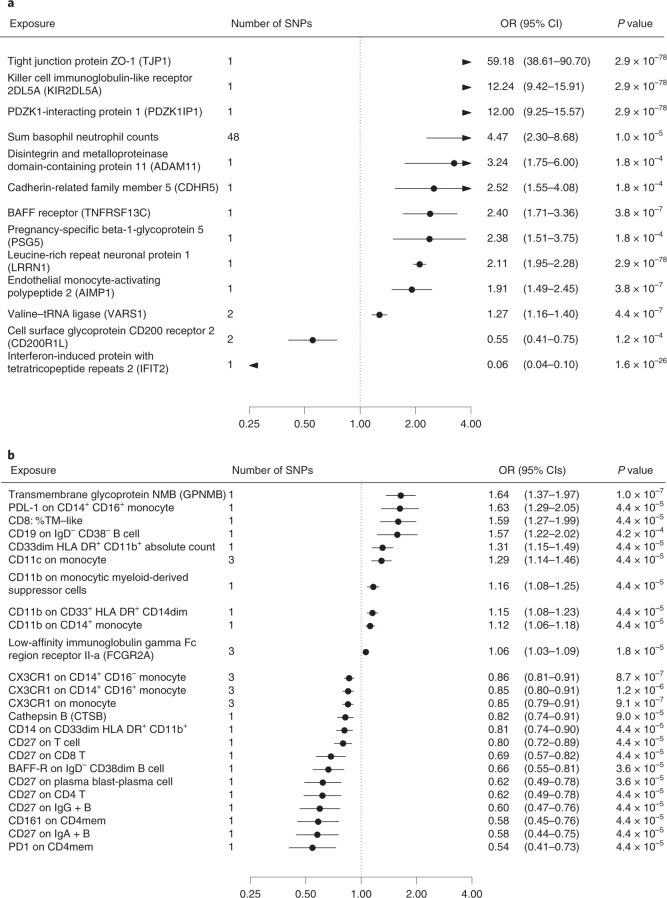
Biomarkers associated with general Alzheimer´s and Parkinson´s disease in Mendelian randomization analyses. **a**,**b**, Odds ratios (ORs) and 95% confidence intervals (CIs) for an increase of 1 s.d. in biomarkers associated with general Alzheimer’s disease outcome (**a**) and Parkinson’s disease (**b**) in MR after FDR correction of 5% (*P* < 0.00052). ORs were derived from Wald ratios when only one SNP was available, and from IVW estimates when two or more SNPs were available. All tests are two-sided.

**Fig. 3:**
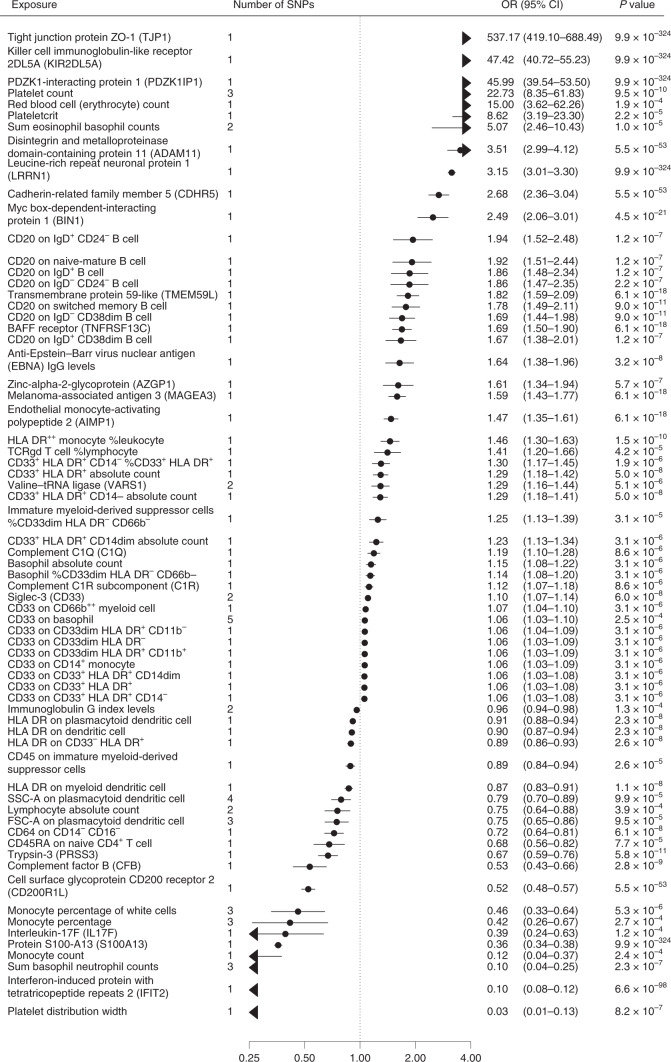
Biomarkers associated with late-onset Alzheimer´s disease in Mendelian randomization analyses. ORs and 95% CIs for an increase of 1 s.d. in biomarkers associated with late-onset Alzheimer’s disease in MR after FDR correction of 5% (*P* < 0.00052). ORs were derived from Wald ratios when only one SNP was available, and from IVW estimates when two or more SNPs were available. All tests are two-sided.

**Extended Data Fig. 1:**
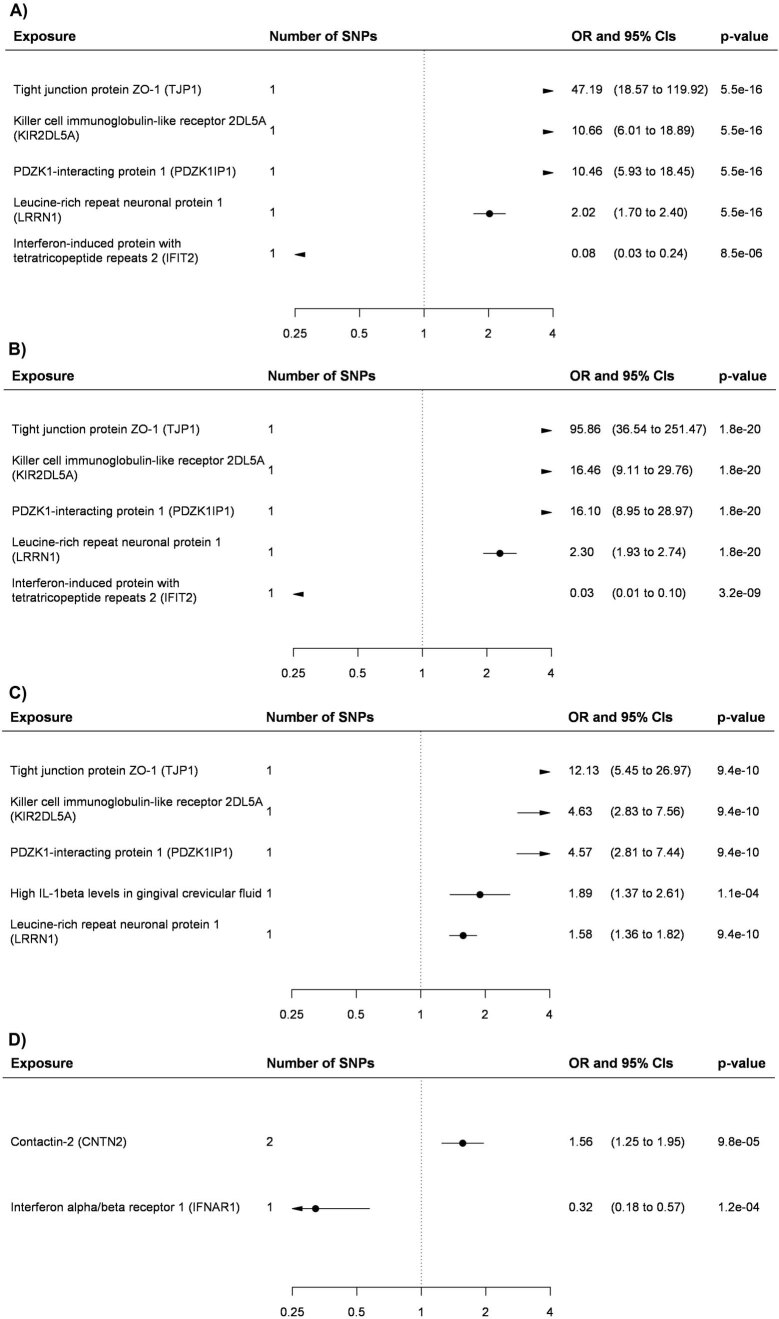
Odds ratios and 95% confidence intervals per one standard deviation increase in biomarker level derived from Wald ratios when only one SNPs was available and from inverse variance weighted Mendelian randomization when two or more SNPs were available. All biomarkers passed false discovery rate correction of 5% (p-value < 0.00052) and all the tests were two-sided. (**A**) atypical or mixed Alzheimer’s disease, (**B**) early onset Alzheimer’s disease, (**C**) vascular dementia, (**D**) frontotemporal dementia.

**Extended Data Fig. 2:**
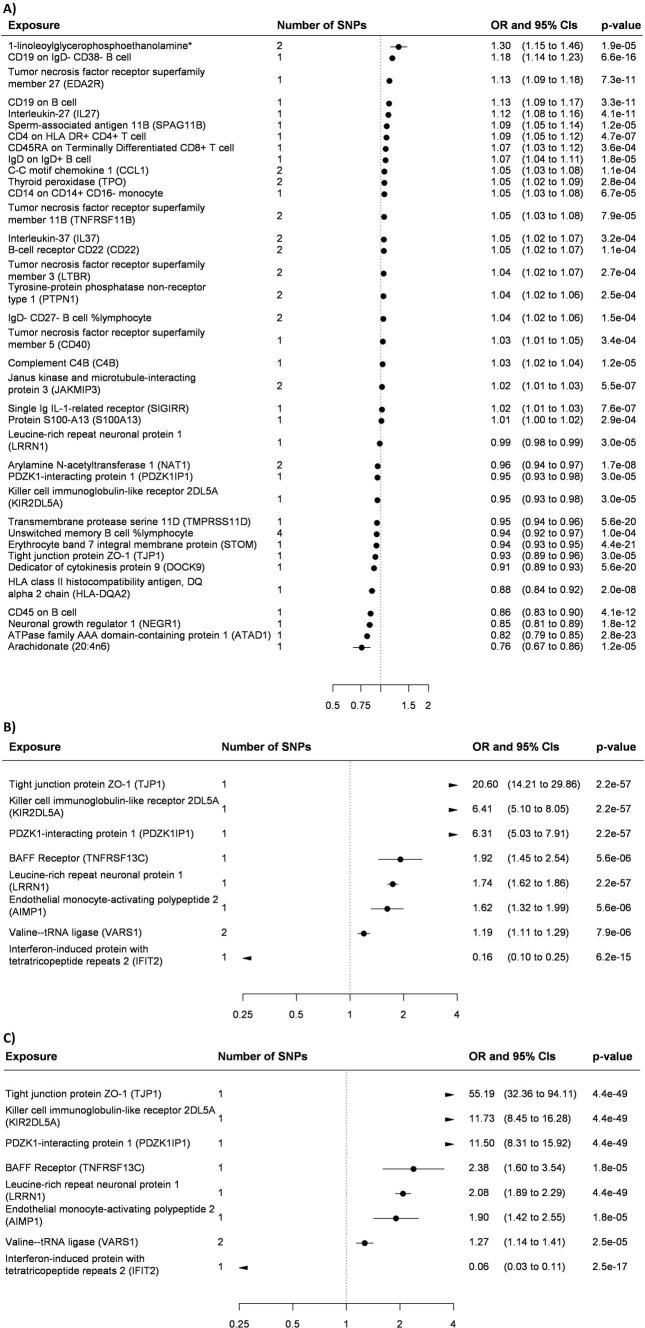
Odds ratios and 95% confidence intervals per one standard deviation increase in biomarker level derived from Wald ratios when only one SNPs was available and from inverse variance weighted Mendelian randomization when two or more SNPs were available. All biomarkers passed false discovery rate correction of 5% (p-value < 0.00052) and all the tests were two-sided. (**A**) continuous cognitive performance, (**B**) general dementia outcome, (**C**) dementia in Alzheimer’s disease.

**Extended Data Fig. 3:**
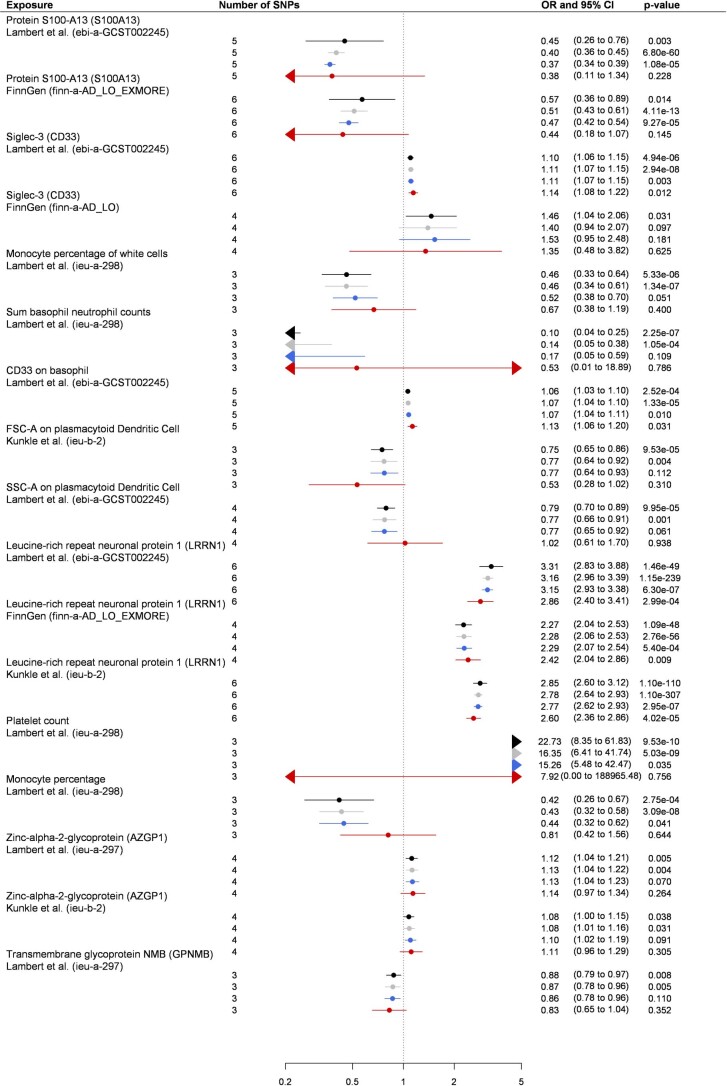
Odds ratios and 95% confidence intervals between one standard deviation change in biomarker levels and late onset Alzheimer’s disease. Results are from Mendelian randomization sensitivity analyses when at least 3 SNPs were available. All biomarkers passed false discovery rate correction of 5% (p-value < 0.00052) in inverse variance weighted Mendelian randomization and all the tests were two-sided. The source of outcome and MR-base outcome identifier is described below the Biomarker. Black = inverse variance weighted, grey = weighted median, blue = weighted mode, red = Egger Mendelian randomization derived estimate.

**Extended Data Fig. 4:**
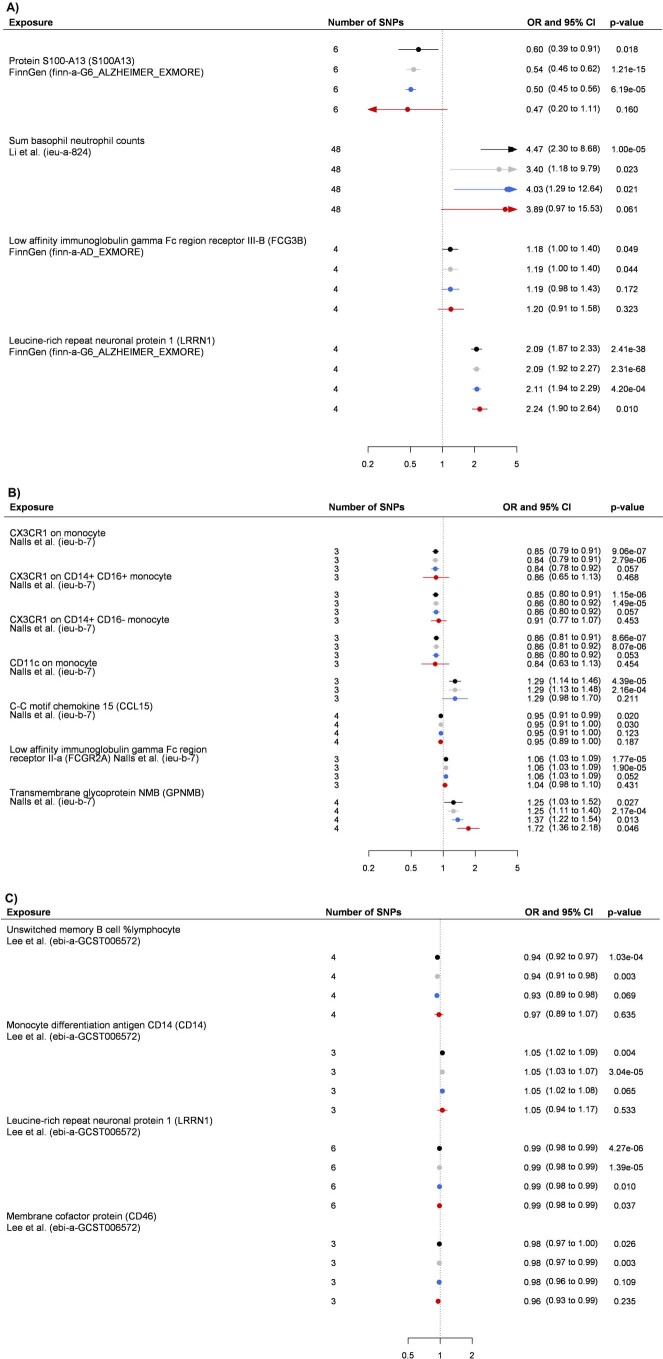
Odds ratios and 95% confidence intervals between one standard deviation change in biomarker levels and (A) Alzheimer´s diseases, (B) Parkinson’s disease, and (C) cognitive performance. Results are from Mendelian randomization sensitivity analyses when at least 3 SNPs were available. All biomarkers passed false discovery rate correction of 5% (p-value < 0.00052) in inverse variance weighted Mendelian randomization and all the tests were two-sided. The source of outcome and MR-base outcome identifier is described below the Biomarker. Black = inverse variance weighted, grey = weighted median, blue = weighted mode, red = Egger Mendelian randomization derived estimate. Egger Mendelian randomization estimate for CD11c on monocyte is omitted because it did not converge.

**Extended Data Fig. 5:**
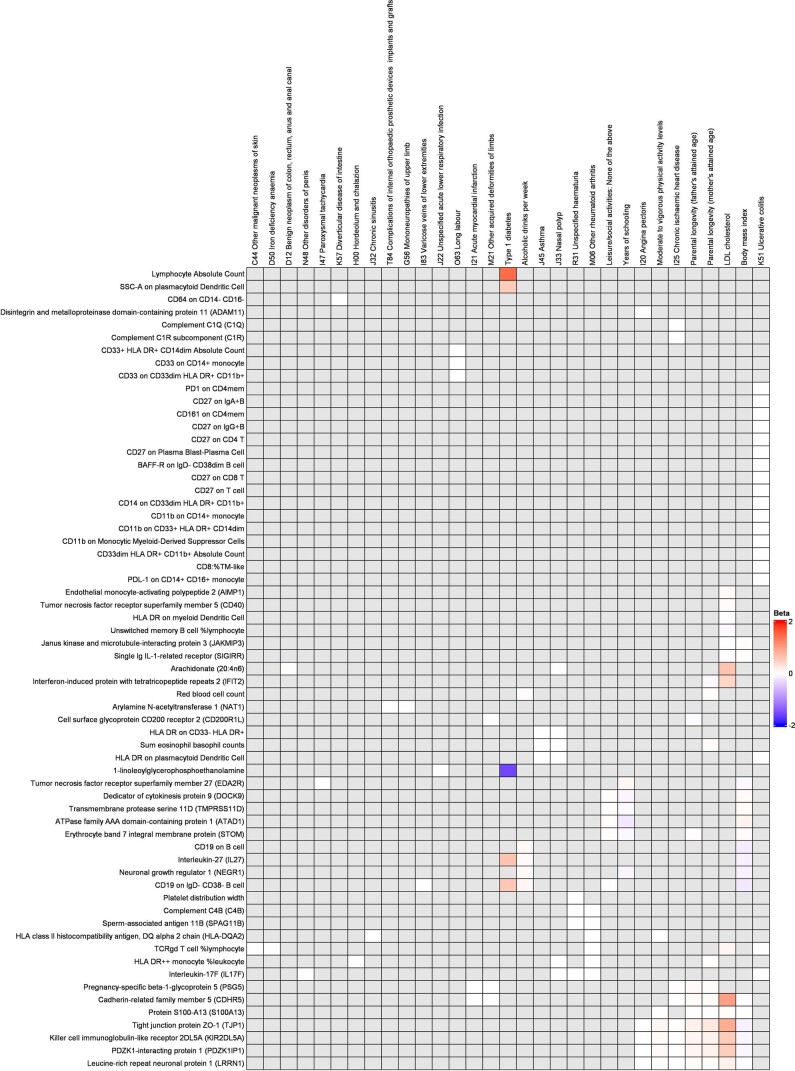
Phenome-wide Mendelian randomization analyses for the 127 biomarkers that associated with dementia causing diseases. Betas are derived from Wald ratios when only one SNP was available and from inverse variance weighted Mendelian randomization when two or more SNPs were available. Results are presented for the 63 of the 127 biomarkers that passed false discovery rate correction of 5% (p-value < 0.00029). Most biomarkers associated with only few outcomes and the grey boxes indicate no association after false discovery rate correction of 5% (p-value < 0.00029) or lack of common SNPs. All the tests were two-sided.

In the discovery step, we used MR to identify potential causal risk factors for dementias. The MR-Base search provided 1,140 biomarkers for the immune system (Supplementary Data 1) and 687 biomarkers for BBB (Supplementary Data 2). A total of 253 immune system and 130 BBB biomarkers passed the false discovery rate (FDR) correction of 5%. After removal of duplicates, 127 unique biomarkers remained: 69 were related to immune cells, five to tumor necrosis factors, five to immunoglobulins, five to interleukins, five to cell membranes, four to complement components, three to platelet characteristics, two to interferon, two to metabolites, two to adhesion molecules, one to chemokines, one to endothelium, one to erythrocyte characteristics and 22 to other immune system- or BBB-related processes. All the biomarkers associated with dementia-causing diseases at *P* <0.00052 in MR analyses using either the Wald ratio (when only one single-nucleotide polymorphism (SNP) was available) or the inverse-variance-weighted method (IVW) (when two or more SNPs were available) (Figs. 2 and 3, Extended Data Figs. 1 and 2 and Supplementary Data 3 and 4). While for three outcomes there was evidence of horizontal pleiotropy, MR sensitivity analyses showed no strong evidence of reverse causality for any of the biomarkers (Supplementary Table 1 and Extended Data Figs. 3 and 4). Of the 127 biomarkers, 49 were proteins, and for these, we identified 25 *cis* and 71 *trans* protein quantitative loci (pQTLs) (Supplementary Data 5). The *cis* loci were for AZGP1, BIN1, C1R, C4B, CFB, CD33, CD40, CNTN2, FCGR2A, GPNMB, IFNAR1, IL-27 and NEGR1. Four of these—AZGP1, CD33, FCGR2A and GPNMB—had three or more SNPs available and passed MR sensitivity analyses. In addition, several CD20- and CD33-expressing leukocytes increased the risk of Alzheimer’s disease; CD11-expressing leukocytes increased, and CD27- and CXCR1-expressing leukocytes decreased, Parkinson’s disease risk in MR sensitivity analyses. The few off-target associations for the 127 biomarkers (FDR < 5%) were mainly with type 1 diabetes and low-density lipoprotein cholesterol (Extended Data Fig. 5).

**Fig. 4:**
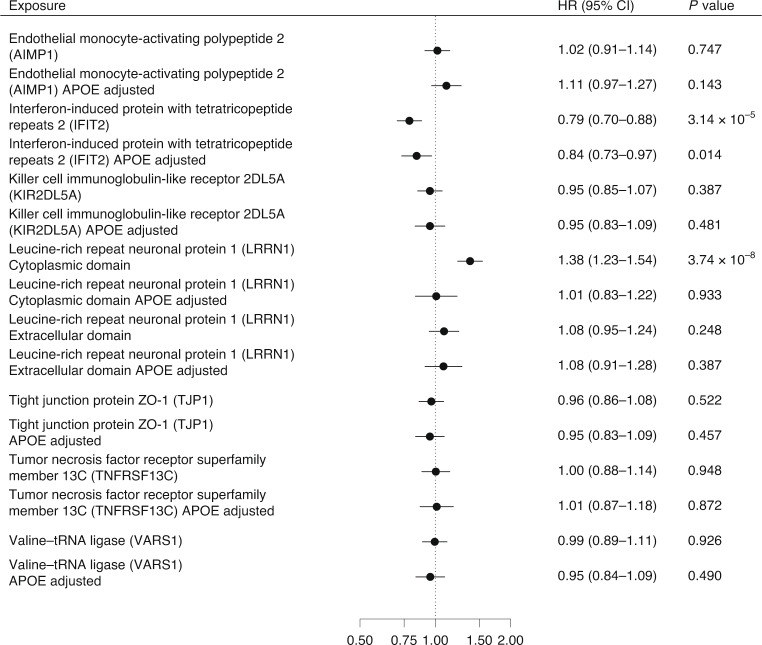
Association between plasma proteins that had pQTLs within 500 kb from *APOE* gene and dementia. Hazard ratios and 95% CIs for association between an increment of 1 s.d. in plasma protein levels and dementia in the Whitehall II cohort. The analyses included eight proteins with pQTLss clustered around *APOE* and that were associated with at least three dementia subtypes in MR analyses. Analyses were first adjusted for age and sex and then additionally for *APOE* status. This analysis was not corrected for multiple testing, and all the tests are two-sided.

Eight proteins were associated with all-cause dementia outcome and their pQTLs were centered within 500 kilobases (kb) from the *APOE* gene, one of the strongest genetic risk factors for late-onset Alzheimer’s disease (Supplementary Data 6) reduced. To examine whether these associations were attributable to the effects of *APOE,* we measured plasma proteins associated with these eight pQTLs in the Whitehall II cohort study (*n* = 6,545). The study included as an outcome a 20-year follow-up of all-cause dementia but did not have data on dementia subtypes. Of these eight proteins, two (LRRN1 and IFIT2) were associated with dementia and one (IFIT2) remained significantly associated with reduced risk of dementia after adjustment for *APOE* status (Fig. 4).

The remaining 119 non-*APOE*-linked biomarkers were more outcome specific and did not show similar enrichment around high-risk genes for Alzheimer’s disease, including *APP*, *PSEN1*, *PSEN2*, *ADAM10*, *TREM2*, *PLD3* and *UNC5C*. Instead, these were characterized by inflammatory, chemokine, complement and adhesion processes (C1Q, C1R, C4B, CCL1, CDHR5, GPNMB, IL-1β, IL-17, IL-27, IL-37, LTBR, PTP1B and SIGIRR), antigen-presenting and immune checkpoints (HLA-DR, HLA-DQ, BAFFR, C1R, C1Q, CD11, CD19, CD20, CD33, CD40, CX3CR1, PD-1 and PDL-1) and BBB tight-junction-related biomarkers (TJP1, AIMP1 and BIN1).

### Pathway analyses

**Extended Data Fig. 6:**
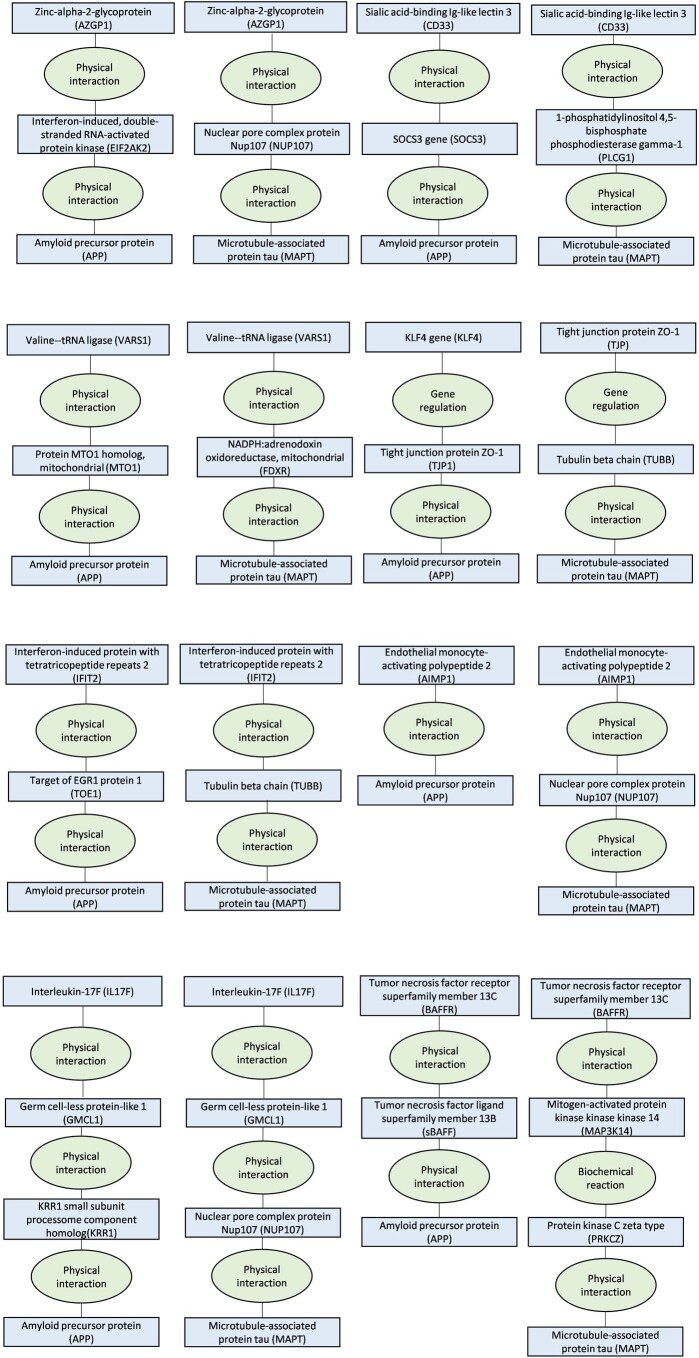
ConsensusPathDB shortest interaction path analyses for the first 8 of the 26 proteins that were associated with Alzheimer’s diseases in Mendelian randomization analyses. The figure describes shortest interaction path between biomarkers and amyloid and tau.

**Extended Data Fig. 7:**
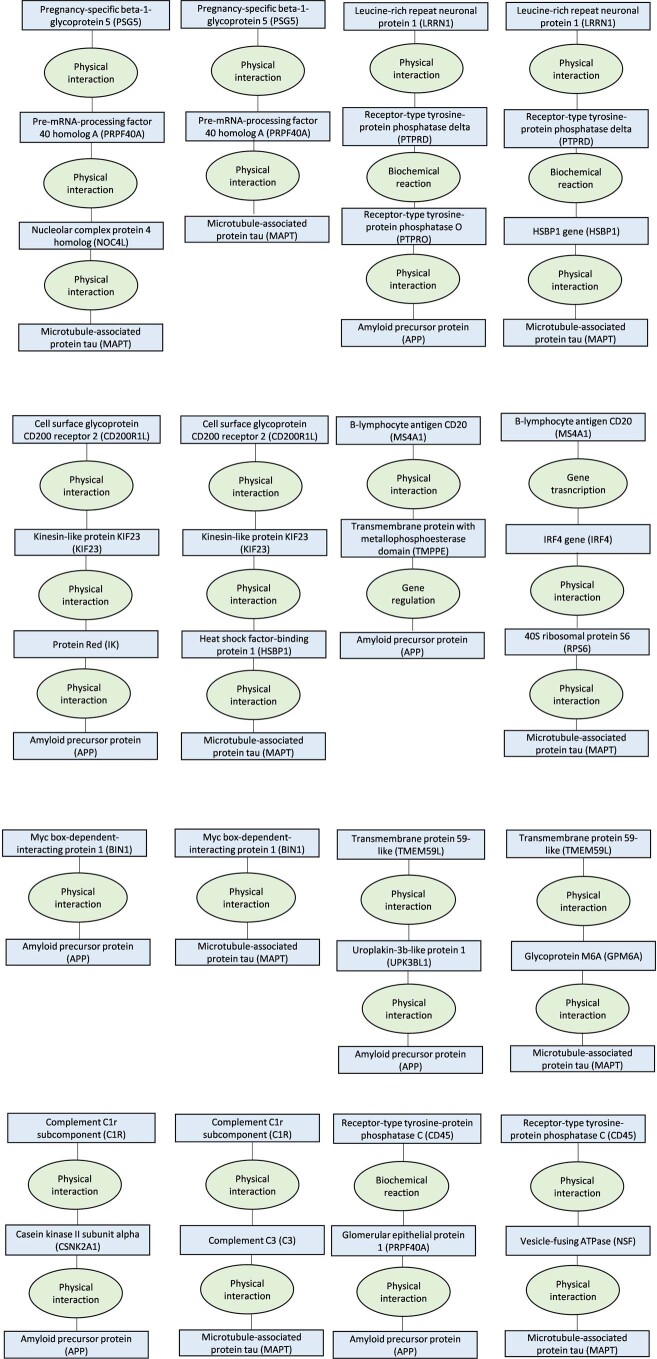
ConsensusPathDB shortest interaction path analyses for additional 8 of the 26 proteins that were associated with Alzheimer’s diseases in Mendelian randomization analyses. The figure describes shortest interaction path between biomarkers and amyloid and tau.

**Extended Data Fig. 8:**
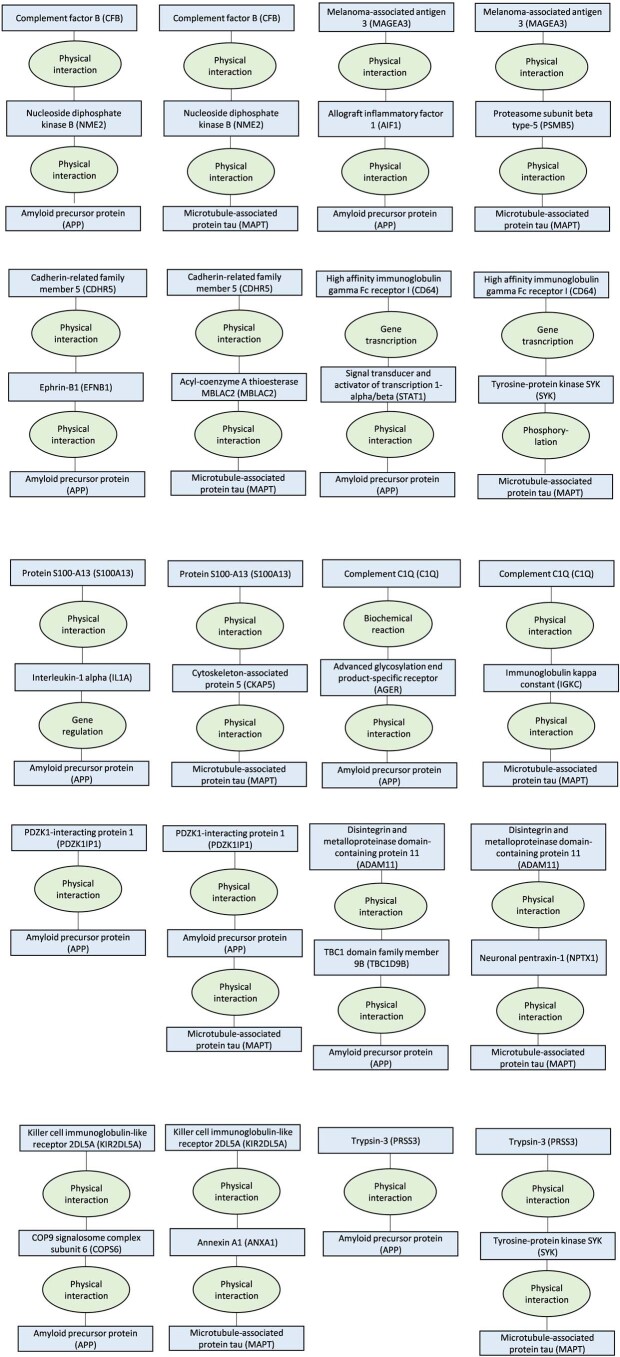
ConsensusPathDB shortest interaction path analyses for the last 10 of the 26 proteins that were associated with Alzheimer’s diseases in Mendelian randomization analyses. The figure describes shortest interaction path between biomarkers and amyloid and tau.

**Extended Data Fig. 9:**
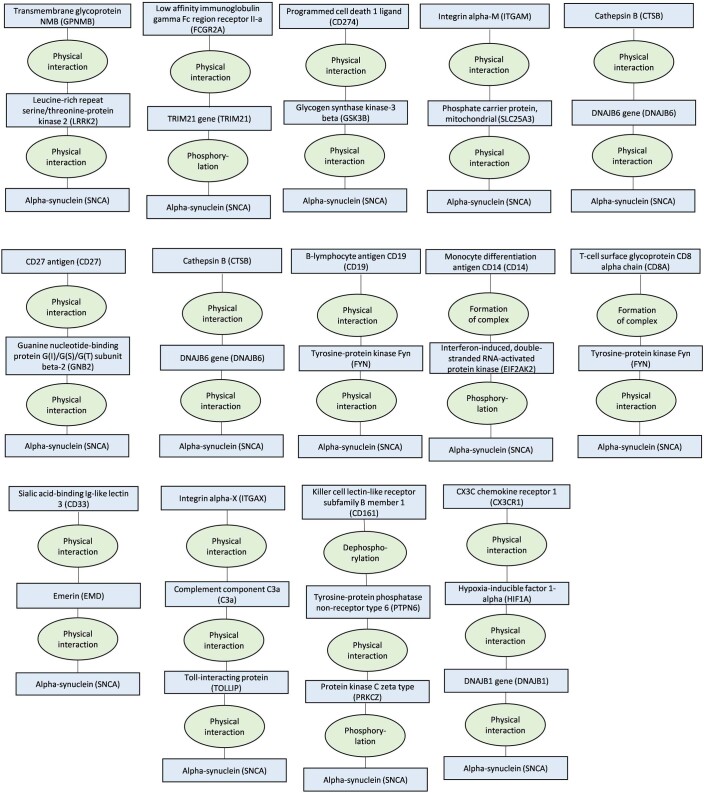
ConsensusPathDB shortest interaction path analyses for the 14 proteins that were associated with Parkinson’s diseases in Mendelian randomization analyses. The figure describes shortest interaction path between biomarkers and α-synuclein.

**Extended Data Fig. 10:**
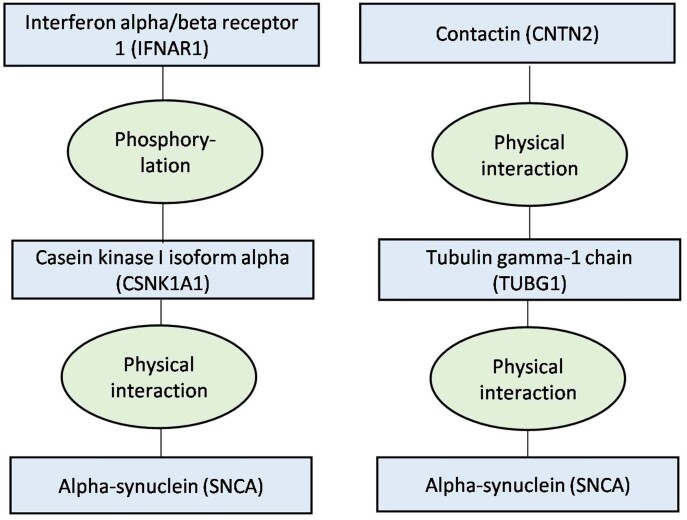
ConsensusPathDB shortest interaction path analyses for the 2 proteins that were associated with frontotemporal dementia in Mendelian randomization analyses. The figure describes shortest interaction path between biomarkers and α-synuclein.

We then used ConsensusPathDB to test whether 42 proteins of the 127 biomarkers that were not bound to any cell and were associated with frontotemporal dementia, Alzheimer’s or Parkinson’s disease play a role in pathways leading to amyloid precursor protein, tau protein or α-synuclein that characterize these diseases. These analyses showed that all of the proteins shared a common pathway and were within only zero to two molecules distance from these proteins, providing additional support for the link between proteins and dementia-causing diseases (Extended Data Figs. 6–10).

To identify other biological processes that may be regulated by the 127 biomarkers, we performed analyses based on Kyoto Encyclopedia of Genes and Genomes (KEGG), ClueGO and ConsensusPathDB databases using the 78 biomarkers that were plasma proteins or receptors on a cell and thus had an ID applicable for analysis. These analyses suggested that the biomarkers are involved in several processes of autoimmunity, ranging from hematopoiesis to self-tolerance and antigen processing and presentation. These included increased HLA-DR expression (a risk allele for several autoimmune diseases) across all hematopoietic cell lines; MHC-II-mediated antigen presentation (a key mechanism that is dysfunctional in autoimmune diseases) in several processes, including autoimmune diseases and responses to infection; increased neuronal adhesion molecule CNTN2 and increased PD-L1 in T cell–antigen interactions that reduce self-tolerance. Furthermore, the biomarkers also altered expression of several cluster differentiation molecules on leukocytes and decreased barrier-protecting IL-17F, self-tolerance-increasing PDCD1 in T cell–antigen interactions and antiviral complement factor B and IFNAR1 (Supplementary Figs. 1 and 2).

Based on MR and pathway analyses, we hypothesized that diseases causing dementia have an inflammatory autoimmune component.

### PRS and HLA analysis

**Fig. 5:**
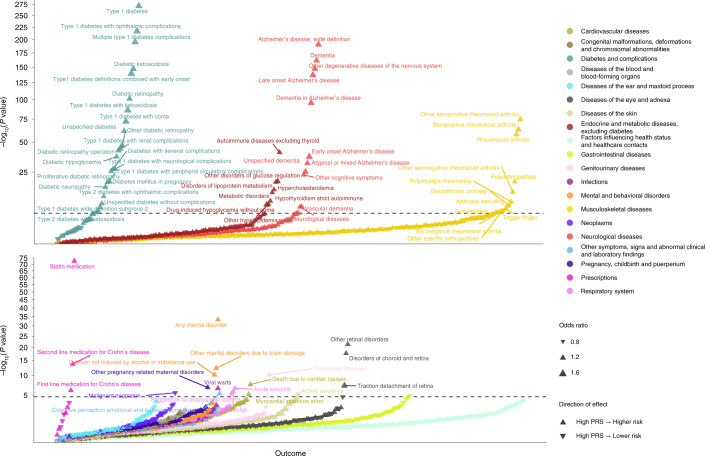
Phenome-wide association analyses for Mendelian randomization-based Alzheimer´s diseases risk polygenic risk score. Phenome-wide association analyses for MR–PRS constructed from SNPs associated with levels of causal Alzheimer’s disease biomarkers in MR Wald ratio or IVW analyses. ORs and –log_10_ *P* values are presented. Upward- and downward-pointing triangles denote increasing and decreasing risk, while larger triangles indicate larger effect size. This analysis was not corrected for multiple testing, and all tests are two-sided.

To further study autoimmunity and the combined effects of the 127 biomarkers, we created an MR-based polygenetic risk score (MR–PRS) using SNPs associated with biomarkers and then performed phenome-wide association analysis (PheWAS) (Fig. 5). PRSs were created separately for each dementia-causing disease using only SNPs linked to outcome-specific biomarkers. After linkage disequilibrium (LD) pruning, excluding extreme SNPs with beta >1.34 and SNP matching in FinnGen (*n* = 339,233), 92 SNPs were available for PRS for Alzheimer’s disease. The number of SNPs (≤25) for other outcomes was insufficient for PRS association analysis. In PheWAS analyses, Alzheimer’s PRS was associated with increased risk of all types of dementia-causing diseases and autoimmune diseases, especially rheumatic diseases and type 1 diabetes and its complications, but with reduced risk of cancers. The associations with dementia-causing diseases were largely attributable to three SNPs within 500 kb from the *APOE* region, whereas associations with autoimmune diseases were independent of *APOE* (Supplementary Table 2).

Certain HLA alleles increase risk for autoimmune diseases, including type 1 diabetes and rheumatoid arthritis^18,19^. We therefore ran an HLA allele-wide analyses for dementia-causing diseases to study autoimmunity by an additional method independent of MR and PRS. We identified nine risk HLA alleles for dementia-causing diseases after FDR correction of 5% (*P* < 0.00085) (Table 2). These analyses supported the autoimmune hypothesis.

**Table 2:** ORs and 95% CIs for association between HLA alleles and dementia-causing diseases that survived FDR correction of 5% (*P* < 0.00085). All tests are two-sided

Disease	HLA type	OR (95% CI)	*P* value	FDR *P* value
Alzheimer’s disease	DQB1 05:01	1.11 (1.06–1.17)	6.7 × 10^–5^	0.005
	DRB1 01:01	1.11 (1.05–1.17)	1.5 × 10^–4^	0.007
	DQA1 01:01	1.11 (1.05–1.17)	9.9 × 10^–5^	0.006
Parkinson’s disease	A 03:01	1.13 (1.05–1.22)	8.4 × 10^–4^	0.027
Dementia	DQA1 05:01	0.90 (0.85–0.95)	6.4 × 10^–^^5^	0.005
	DQB1 02:01	0.90 (0.85–0.95)	4.9 × 10^–^^5^	0.005
	DRB1 03:01	0.90 (0.85–0.95)	7.1 × 10^–^^5^	0.005
	DRB1 04:01	0.90 (0.85–0.96)	4.9 × 10^–^^4^	0.020
	DRB4 01:03	0.93 (0.90–0.97)	7.9 × 10^–^^4^	0.027

### IPW analyses

To evaluate the autoimmune hypothesis in relation to modifiability and drug repurposing, we examined whether commonly used anti-inflammatory and immunosuppressive medications are likely to reduce the risk of dementia-causing diseases. For this, we used IPW analyses in the FinnGen study (Table 3 and Supplementary Table 3). As a preliminary step, we validated the IPW protocol with two analyses. The first replicated the RCT effect between statin medication and myocardial infarction (a positive control), and the second replicated the null findings in anti-inflammatory medication trials for cardiovascular diseases (a negative control)^20–23^.

**Table 3:** Hazard ratios and 95% CIs for associations between dementias, methotrexate and TNF-α inhibitor medications from IPW Cox proportional-hazards survival analyses in the FinnGen study

Medication	Outcome	HR (95% CI)	*P* value
**Statins**			
Positive control	Coronary heart disease IPW	0.38 (0.19–0.78)	0.009
	Coronary heart disease RCT	0.39 (0.29–0.49)	7.3 × 10^–12^
**Methotrexate**	Alzheimer’s disease (AD)	0.74 (0.59 –0.93)	0.013
	Alzheimer’s disease including those with AD–MR–PRS ≥50%	0.64 (0.47–0.88)	0.005
	Alzheimer’s disease including those with AD–MR–PRS <50%	0.84 (0.59–1.19)	0.330
	Alzheimer’s disease including those with AD–MR–PRS ≥50%(*APOE* region excluded)	0.75 (0.54–1.04)	0.085
	Alzheimer’s disease including those with AD–MR–PRS ≥50% (*APOE* region excluded)	0.73 (0.52–1.02)	0.065
	Alzheimer’s disease including those with Jansen’s PRS ≥50%	0.70 (0.51–0.97)	0.033
	Alzheimer’s disease including those with Jansen’s PRS <50%	0.80 (0.57–1.12)	0.197
	Alzheimer’s disease including those with Jansen’s PRS ≥50% (*APOE* region excluded) ≥50%	0.74 (0.53–1.04)	0.081
	Alzheimer’s disease including those with Jansen’s PRS ≥50% (*APOE* region excluded)	0.75 (0.54–1.06)	0.102
	Alzheimer’s disease including those with at least one *APOE* ε4 allele	0.69 (0.49–0.97)	0.032
	Alzheimer’s disease with those carrying *APOE* ε4 allele excluded	0.79 (0.57–1.10)	0.168
	Vascular dementia	0.64 (0.30–1.37)	0.253
	Parkinson’s disease	0.48 (0.29–0.81)	0.006
Negative control	Coronary heart disease IPW	0.97 (0.84–1.11)	0.647
	Coronary heart disease RCT	0.96 (0.79–1.16)	0.862
**TNF-α inhibitors**	Alzheimer’s disease	0.32 (0.14–0.76)	0.010
	Vascular dementia	0.27 (0.04–1.91)	0.188
	Parkinson’s disease	0.26 (0.04–1.82)	0.173
Negative control	Coronary heart disease IPW	1.03 (0.55–1.95)	0.916
	Coronary heart disease RCT	1.09 (0.77–1.56)	0.645

Positive-control analyses validate the IPW analysis protocol by replicating the established association between statin medication and reduced coronary heart disease risk. As a further validation step, negative-control IPW analyses replicate the null finding between anti-inflammatory medications and coronary heart disease. In the main IPW analyses, baseline variables were birth year, sex, ten principal components and time-varying variables statin, ACE-blocker, AT-blocker, renin-blocker, calcium channel blocker, any diuretic, insulin, metformin, other diabetes drug, antidepressant, antipsychotic and anticoagulant medication use; as well as time-varying disease diagnosis (any cancer, myocardial infarction, atrial fibrillation, heart failure, venous thromboembolism, ischemic stroke, intracerebral hemorrhage, subarachnoid hemorrhage, obesity, sleep apnea or chronic obstructive pulmonary diseases), with informative censoring included. Analyses included only individuals who did not use anti-inflammatory studied at baseline. The only exception was TNF-α inhibitor analyses, where baseline users were included because of the small number of individuals using this medication in the FinnGen cohort. The analyses here were not corrected for multiple testing, and all tests are two-sided. The estimates for RCTs are from refs. ^20,22,23^. A secondary prevention RCT was used for methotrexate, because no primary prevention trials were available. MR–PRS, MR-based PRS for Alzheimer’s disease.

For the main analysis, we selected all anti-inflammatory medication categories if there were data for at least ten individuals who were treated with the medication and developed dementia-causing disease over the follow-up. Supporting the autoimmune hypothesis, these analyses including 117,773 participants showed that use of methotrexate or TNF-α inhibitors was associated with reduced risk of Alzheimer’s and Parkinson’s disease. Stratifying by *APOE* status, the risk of Alzheimer’s disease was reduced only in individuals with high genetic risk, as indicated by above-median MR– PRS (50% cutoff) or who had at least one *APOE ε4* allele.

To examine whether the effect was dependent on *APOE* status, we repeated the analysis in participants with above-median MR– PRS or above-median more comprehensive PRS (including 1,092,011 SNPs) derived from the largest available Alzheimer’s disease GWAS^24^ and excluded the *APOE* area from these PRSs. These analyses showed null results, suggesting that the protective effect of methotrexate is specific for those with at least one *APOE ε4* allele. The numbers of individuals using TNF-α inhibitors or developing Parkinson’s disease were too small for subgroup analyses.

To allow an additional analysis for rare medications, we searched the Open Targets database for medications that modify the levels of the 127 biomarkers. This search identified 64 drugs (mostly monoclonal antibodies) that target 18 of the 127 biomarkers, suggesting that these may also have potential for repurposing in the treatment of dementia-causing diseases (Supplementary Data 7).

## Discussion

Consistent evidence from six independent studies suggests that inflammatory autoimmunity may play a causal role in dementia-causing diseases. Our MR analyses identified causal support for 127 risk factors including inflammatory, self-tolerance and/or BBB tight-junction-related biomarkers. Pathway analyses linked these 127 biomarkers to autoimmunity via several alterations in processes, from hematopoiesis to antigen presentation and reduced self-tolerance. They also showed that all 42 circulating proteins associated with frontotemporal dementia, Alzheimer’s or Parkinson’s diseases among the 127 biomarkers are closely related to α-synuclein, amyloid precursor and tau protein pathways that characterize these diseases. A phenome-wide analysis of our MR–PRS, constructed from SNPs associated with the identified risk factors for Alzheimer’s disease, indicated shared genetic background with autoimmune diseases, such as rheumatoid arthritis and type 1 diabetes. The autoimmune hypothesis was further supported by HLA analyses showing nine HLA-type associations with dementias. According to IPW analyses mimicking randomized controlled drug trials in observational data, repurposed use of anti-inflammatory or immunomodulatory medications may reduce the risk of Alzheimer’s in individuals with an *APOE ε4* allele. This finding suggests that the inflammatory autoimmune component may be modifiable with currently available medications.

To our knowledge, this is the most comprehensive MR and triangulation study to date on immune system- and BBB-related biomarkers as risk factors of dementia-causing diseases. Our MR focused on 1,827 biomarkers whereas earlier MR analyses included fewer than 200 biomarkers specific to these systems^10,12,25–33^. We obtained the strongest causal evidence for autoimmunity- and inflammation-related AZGP1 (ref. ^34^) and CD33 (ref. ^35^) for Alzheimer’s disease, and for FCGR2A^36^ and GPNMB^37^ in Parkinson’s disease. These proteins had *cis* pQTLs available, and they passed MR sensitivity analyses. For CD33, a monoclonal antibody, gemtuzumab ozogamicin^38^ is in routine clinical use, and it may have potential for drug repurposing in Alzheimer’s disease. To our knowledge, associations of AZGP1 and FCGR2A with dementias have not been reported previously whereas an earlier MR study on CD33 and GPNMB exists^33^. As an additional supportive finding, our MR sensitivity analyses showed that several CD20- and CD33-expressing leukocytes increase the risk of Alzheimer’s disease and that CD11-expressing leukocytes may increase, and CD27- and CXCR1-expressing leukocytes may decrease, Parkinson’s disease risk.

In general, our results provide evidence on the role of BBB in the etiology of dementia-causing diseases by suggesting that higher plasma levels of the tight junction component TJP1 (ref. ^39^) and proteins degrading the tight junction, such as AIMP1 (ref. ^40^) and BIN1 (ref. ^41^), increase—and higher levels of barrier-protecting IL-17F^42^ reduce—the risk of Alzheimer’s disease. These findings suggest potential causal risk factors that support the BBB dysfunction and barrier breach hypothesis^9,43^, linking BBB breakdown to subsequent inflammatory and autoimmune responses in the CNS. The results are also in line with experimental studies that have linked cerebral vascular dysfunction to cognitive decline, and with evidence linking BBB dysfunction in the hippocampal area with increased risk of Alzheimer’s disease independent of amyloid-β or tau^44–46^.

In agreement with previous research, several proinflammatory biomarkers were associated with increased risk of dementia-causing diseases. IL-1β^47^ increased the risk for vascular dementia; C1Q, C1R, CD20 and CDHR5 (refs. ^48–50^) the risk for Alzheimer’s disease; and GPNMB and CD11b^37,51^ the risk for Parkinson’s disease^37,51^. Anti-inflammatory biomarkers C4B, IL-27, IL-37, PTP1B and SIGGIRR^52–56^ in turn improved cognitive performance. In addition, our MR analyses identified checkpoint regulators BAFFR, C1R, C1Q, CD11, CD19, CD20, CD22, CD33, CD40, CX3CR1, LTBR, PD-1 and PDL-1^51,57–66^ as potential causal risk factors for poor cognitive performance and dementia-causing diseases, uncovering the importance of checkpoint control and potential sources of autoreactivity.

Previous MR studies on Alzheimer’s disease^10,12,25–33^ suggest potential causal associations with BIN1, CCL27, C3, CD33, CD4 T cells, GDF-15 and SVEP1, whereas MR studies on Parkinson’s disease suggest a potential causal association with GPNMB, IL-6 and MIP1b. Compared with these studies, we used a stricter *P* value cutoff with multiple testing correction and were able to replicate associations between BIN1, CD33 and Alzheimer’s disease and those between GPNMB and Parkinson’s disease. Our MR analyses did not replicate the results of other biomarkers. Potential reasons for this discrepancy include the use of different sets of SNPs to test associations between these biomarkers and dementia-causing diseases, and differences in population characteristics.

Pathway analyses provided further understanding of processes that may be regulated by the identified biomarkers. In line with the MR results, these analyses revealed that the biomarkers were enriched in inflammatory and autoimmunity-related biological processes including altered hematopoiesis, cytokine–receptor interaction, responses to infections, self-tolerance, phagosome processing, cell adhesion and antigen presenting, transferring them towards increased autoreactivity. The analyses also showed that the biomarkers were involved in amyloid-β, tau protein and α-synuclein pathways that characterize diseases causing dementia. This converging support for the autoimmune hypothesis from the MR and pathway analyses adds to previous limited human evidence on this hypothesis^43,67^.

We obtained additional insights into autoimmune hypothesis from four further studies. Strengthening the supportive evidence, our PheWAS analyses showed that several autoimmune diseases share immune-related genetic background with dementia-causing diseases. In addition, MR–PRS for dementia-causing diseases was associated with reduced risk of cancers, which is a well-described beneficial side effect of reduced self-tolerance commonly harnessed in immune-oncological cancer medications^68^.

The association between MR–PRS and dementia-causing diseases was driven by SNPs in the *APOE* region that are associated with several BBB- and autoimmunity-related proteins such as IFIT2 (ref. ^69^), LRRN1 (ref. ^70^), TJP1 (ref. ^39^), KIR2DL5A^71^, AIMP1 (ref. ^72^) and BAFF receptor^57^, suggesting that the autoimmune component in *APOE ε4* allele carriers could be related to these proteins. The findings on these proteins should be interpreted cautiously, because only LRRN1 and IFIT2 were replicated using plasma proteins and only the protective effect of IFIT2 was independent of the *APOE* gene. IFIT2 is protective of viral infections^69^ and may reduce the load of acute and chronic inflammation in the CNS^73^, suggesting that it may be a promising *APOE*-independent drug target.

The autoimmunity hypothesis was further supported by HLA allele-wide analyses that identified nine HLA types associated with dementia-causing diseases. By identification of the specific risk alleles from HLA classes, these results complement previous studies^74–77^ that have identified HLA-DR and HLA-DQ as risk factors for dementia-causing diseases.

### Anti-inflammatory medications and dementias

We obtained evidence on the modifiability of the autoimmunity–dementia association in analyses simulating RCTs using observational data. The validity of these IPW survival analyses was supported by expected results from the positive and negative control analyses. To ensure sufficient data on key variables (positivity condition) and well-defined interventions and outcomes (consistency condition), we used linkage to electronic health records from high-quality, nationwide registries of filled drug prescriptions and diseases outcomes. To simulate an RCT, we included only dementia-free individuals with no history of the investigated anti-inflammatory medications at baseline and followed them up over 20 years. Thus, the analyses simulated a two-decade randomized trial focusing on the effect of preventative anti-inflammatory medication on the risk of dementia-causing diseases. The analyses supported the autoimmune hypothesis and suggested that inflammatory autoimmune processes are modifiable. More specifically, we were able to replicate the associations between the use of TNF-α inhibitors and^78^ methotrexate^79^ and reduced risk of Alzheimer’s disease and to provide evidence of the potential benefits of methotrexate in Parkinson’s disease.

As a previously unreported finding, we showed that the protective effect of methotrexate in Alzheimer’s disease is observed only in those who carry at least one *APOE ε4* allele. This finding is in line with experimental studies on the effects of the *APOE ε4* allele and methotrexate on the BBB and immune system. Individuals carrying the *APOE ε4* allele have been shown to have higher rates of BBB and immune system dysfunction^80^, whereas methotrexate targets both these vulnerabilities by protecting the BBB and being anti-inflammatory. These mechanisms are thought to improve endothelial integrity and regulatory T cell differentiation, inhibition of neutrophil adhesion and recruitment, cytokine expression in macrophages, T cell activation, T cell-mediated cell death and metalloproteinase production^81^, all mechanisms highlighted in our MR and pathway findings. These findings suggest that future RCTs on the preventative potential of methotrexate against Alzheimer’s disease might be feasible and should include only people with at least one *APOE ε4* allele. To date, no RCTs are available for methotrexate as a therapeutic treatment for dementia-causing diseases.

TNF-α inhibitors are safe and well tolerated in patients with Alzheimer’s disease dementia (phase 2 trial), but no evidence of benefits is available from studies with 1- to 2-year follow-up^82^. Similarly, RCTs with 1- to 2-year follow-up on corticosteroids, nonsteroidal anti-inflammatory drugs and anti-inflammatory minocycline that have included participants with cognitive decline or Alzheimer’s disease have shown no benefit^83–85^. A key strength of our IPW analysis is the tenfold longer follow-up. To reduce tissue damage in autoimmune diseases, early initiation of anti-inflammatory medication is of paramount importance. The protective effect in long follow-up compared with a null effect in short follow-up is in line with studies of autoimmune diseases^19^ and suggests that medication in trials for people with established dementia may come too late. In the future, trials on the effectiveness of anti-inflammatory medication in dementia-causing diseases should include high-risk individuals when they are still asymptomatic or present with only early symptoms of the disease. Future studies should also investigate autoimmunity in greater detail to determine the role of central tolerance in major immune organs and the subsequent escape of autoreactive B and T cells to the periphery^59,86^, neoepitopes and autoimmune risk-increasing HLA alleles^19,87,88^ as well as peripheral self-tolerance mechanisms, such as ignorance, anergy, suppression, inhibition and antigen presentation^59,86^. Such studies may identify antigen-specific therapeutic strategies that offer new avenues in the search for treatment for dementia-causing diseases.

### Strengths and limitations

Combining multiple lines of research allowed us to examine the role of immune system and BBB biomarkers in the etiology of dementia-causing diseases and to identify potential new drug targets and opportunities to repurpose existing medications for these diseases. The use of an MR approach across 1,827 biomarkers contributed to the evaluation of causality^16^. The findings were summarized with KEGG and ClueGO analyses, both pointing to autoimmune processes. ConsensusPathDB, one of the most comprehensive collections of databases on molecular pathways and interactions^89^, linked the biomarkers to proteinopathies in dementia-causing diseases. Plasma protein analyses allowed us to adjust effect estimates for the *APOE* genotype for certain proteins. PRS and HLA analyses used data from the FinnGen study, with a sample size of 340,000 providing sufficient statistical power. The medication analyses in 120,000 FinnGen participants relied on the IPW method, which is proposed to provide more reliable causal estimates than traditional survival analyses of observational data^90^.

Our study also has limitations. Rather than having a single dataset with complete information, we used an approach in which separate analyses were performed in separate cohorts. This heterogeneity in study samples and assessment of biomarkers and outcomes is a potential source of inconsistent results, but simultaneously, convergent findings across different studies and methodological approaches support the robustness and generalizability of the results. Although we explored 1,827 biomarkers related to the BBB and immune system, we may have missed some biomarkers due to limited numbers of immune system- and BBB-related biomarkers captured by our free text field searches or lack of SNPs available. In addition, MR provides unconfounded estimates if the genetic variants being used as an instrument for exposure are associated with that exposure but not with confounding factors, and there is no independent pathway between the genetic variants and the outcome other than through the exposure. While the first assumption was confirmed in the present study, it is not possible to exclude potential violations of the latter two assumptions. Many MR analyses had a limited number of SNPs, which increased the probability of chance findings and did not allow MR sensitivity analyses for some biomarkers. However, for biomarkers with multiple SNPs, only three showed evidence of horizontal pleiotropy. Future studies with access to fine-mapping results from protein GWASs and to in-sample LD data to perform fine-mapping on the summary statistics should further examine causality and drug targets using colocalization analyses.

Ascertainment of dementia was based on linkage to electronic health records. Although this has the advantage of providing data for everyone recruited to the study, it misses participants with milder dementia and is not the gold standard method for assessment of dementia subtypes. Furthermore, because the onset of late-onset dementias in cohort studies is often at an older age than mean age at death, our results may be subject to collider bias potentially underestimating the role of risk factors that affect longevity, including systemic inflammation, in the development of diseases causing dementia. Our plasma protein analyses were limited by lack of data on dementia subtypes. The PRS and PheWAS analyses were done on samples with European ancestry and may not apply across different ancestries. Due to the limited number of SNPs included in PRS, we may have missed important immune system- and BBB-related associations in our phenome-wide analyses. The IPW analyses on medications may include some bias due to a limited number of individuals in medication subgroups and to limitations in covariate data for simulation of RCTs. However, major bias is unlikely because the analysis protocol was supported by positive- and negative-control analyses.

In summary, this study provides new insights into autoimmunity, BBB and inflammatory dysfunction as contributors to the development of diseases causing dementias. These components are potentially modifiable with medications, suggesting that anti-inflammatory medications and antigen-specific prevention strategies may offer new avenues in the search for treatment for dementias. The present investigation generated new hypotheses on several specific drug targets for dementia-causing diseases, but these need to be validated in future experimental studies. In particular, RCTs assessing the benefits of early autoimmunity-targeted therapies for high-risk individuals are warranted.

## Methods

### MR

The SNPs for biomarkers and outcomes were searched from the MR-Base database^91^. Immune system and BBB search terms were identified using identifiers of cell types, receptors, proteins, metabolites and genes. Identifiers were searched from the literature^4,7,9,10,86,88,92–94^ and the UniProt database^95^ using the search terms ‘immune’ and ‘blood brain barrier’. A complete list is available in Supplementary Data 1 and 2. Outcomes were diseases causing dementia, including the following conditions: all types of Alzheimer’s disease, Parkinson’s disease, vascular dementia, frontotemporal dementia, dementia in general and progression of dementia. Cognitive performance was chosen as an intermediate outcome. Additional SNPs for sensitivity analyses were searched from full summary statistics of three additional plasma-proteome-wide studies^96–98^. Two-sample MR was used to analyze associations between biomarkers and outcomes^16^. The first analyses estimated effects using the Wald ratio or IVW analyses^91^. We applied a FDR correction of 5% for the total number of tests conducted within each biomarker class, leading to cutoffs of *P* < 0.00043 and *P* < 0.00052 for immune system- and BBB-related biomarkers, respectively. For biomarker–outcome pairs that passed FDR of 5% but shared fewer than three SNPs, we performed sensitivity analyses with backward MR. For biomarker–outcome pairs with three or more shared SNPs, we performed additionally weighted median, weighted mode and MR Egger analyses^16^ using the R packages TwoSampleMR and MRInstruments. To assess potential off-target effects for the observed causal biomarkers, we performed phenome-wide MR analyses separately for each biomarker using Neale laboratory GWAS summary statistics for 210 UK Biobank endpoints. The phenome-wide outcomes in these analyses also included recognized risk factors for dementia-causing diseases^93,99^.

In all analyses, we used individuals of European ancestry, a clumping cutoff *R*^2^ of 0.01 and a 500-kb window. LD proxies were searched with a threshold of *R*^2^ = 0.6 and a proxy split size of 500. Biomarkers and outcome alleles were harmonized by inference from positive-strand alleles using allele frequencies for palindromes. For these analyses, we used statistical software R (3.6.0 and 4.1.0). The novelty of MR findings was examined by systematic PubMed search using the following search terms: (Mendelian randomization) AND (dementia OR Alzheim* OR Parkin* OR cognitive decline) AND (Entrez gene symbol OR UniProt protein name) without limitations.

### Pathway analyses

We used KEGG pathway analysis with Generally Applicable Gene-set Enrichment^100^ to study the effect of biomarkers on validated pathways. We used MR Wald ratios or IVW betas and *P* values as input for expression ratios. Gene Ontology term enrichment analyses were done with ClueGO v.2.5.8 (ref. ^101^) in Cytoscape v.3.7.2 (ref. ^102^). In the hypergeometric test, we used 78 of the 127 biomarkers that were plasma proteins or receptors on a cell and thus had an ID applicable for these analyses as input, and all immune system- and BBB-related proteins from UniProt^95^ as background and a correction for 5% FDR. The shortest interaction path analyses were done with ConsensusPathDB^89^, a web-based analysis tool containing a range of biomedical databases. ConsensusPathDB was used to decipher potential common pathways between biomarkers and amyloid-β, tau protein and α-synuclein.

### Plasma protein analyses

Plasma protein measurements in the Whitehall II study were available for 6,545 individuals of whom 310 developed dementia^7,103–105^. The participants were linked to the National Health Service (NHS) Hospital Episode Statistics (HES) database and the UK national mortality register using individual NHS identification numbers for linkage^103^. The NHS provides almost complete health care coverage for all individuals legally resident in the UK. We defined incident dementia using the WHO International Classification of Diseases, revision 10 (ICD-10) codes F00, F01, F03, G30 and G31 and ICD-9 codes 290.0–290.4, 331.0–331.2, 331.82 and 331.9. We also conducted informant interviews and checked participants’ medications at each screening (in 1996–1998, 2011–2013 and 2016–2017) for dementia-related medication. Sensitivity and specificity of dementia assessment based on HES records are 0.78 and 0.92, respectively^104^.

Plasma proteins were measured using SomaScan v.4.0 and v.4.1 assays^7,106,107^. Assays were validated against an external reference population, and protein-specific conversion coefficients were used to balance technical differences between versions 4.0 and 4.1. The analyses used plasma samples measured in 1997/1999 and stored in 0.25-ml aliquots at −80 °C. Earlier studies have described in detail the performance of the SomaScan assay and the modified aptamer binding^7,105–107^. In brief, the assay uses a mix of thousands of slow, off-rate modified aptamers that bind to proteins in participants’ plasma samples, where specificity is ensured with a two-step process analogous to a conventional immunoassay. The specificity of aptamer reagents is good and has been confirmed in several ways^7,108,109^. Median intra- and interassay coefficients of variation for SomaScan v.4 are ~5% and assay sensitivity is comparable to that of typical immunoassays, with a median lower limit of detection in the femtomolar range.

In the Whitehall II study, standard self-administered questionnaires provided data on age and sex. Using DNA extracted from whole blood, a standard PCR assay determined *APOE* genotype using the salting-out method^110,111^. Two blinded independent observers read the genotype, and any discrepancies were resolved by repeating the PCR analysis.

In Whitehall II analyses, we studied the eight proteins associated with all-cause dementia in MR analyses. Dementia subtype data were not available in Whitehall. The distributions of protein values were skewed and therefore transformed to a normal distribution using inverse rank-based normal transformation. The follow-up started at clinical examination in 1997/1999 and ended at onset of dementia, death or 1 October 2019, whichever occurred first. Age, sex and *APOE*-adjusted Cox regression models estimated associations between proteins and diseases causing dementia^112^. The proportionality assumption in Cox models was assessed with Schoenfeld residuals and log–log plots^112^. We used statistical software R (3.6.0 and 4.1.0) for these analyses.

In the Whitehall II study, research ethics approvals were renewed at each wave; the most recent approval was obtained from the University College London Hospital Committee on the Ethics of Human Research (reference no. 85/0938). Written, informed consent from participants was obtained at each contact.

### PRSs and IPW analyses

FinnGen Data Freeze 8 comprises 339,233 individuals and represents approximately 7% of the adult Finnish population. FinnGen is a collection of prospective epidemiological and disease-based cohorts and hospital biobank samples that links genotypes by unique national personal identification numbers to nationwide health registries, including national hospital discharge (available from 1968 onwards), death (1969), cancer (1953) and medication reimbursement (1964) and purchase (1995) registries. The registry-based follow-up ended on 31 December 2020. Alzheimer’s disease was defined with ICD-10 codes under F00 and G30, ICD-9 codes under 3310, ICD-8 code under 29010 and medication purchase Anatomical Therapeutic Chemical (ATC) code N06D; vascular dementia with ICD-10 codes under F01 and ICD-9 codes under 4378; and Parkinson’s disease with ICD-10 codes under G20, ICD-9 codes under 3320 A, ICD-8 code under 34200 and medication reimbursement code 110.

FinnGen samples were genotyped with Illumina and Affymetrix (Thermo Fisher Scientific) arrays. Genotype calls were made with GenCall or zCall (for Illumina) and the AxiomGT1 algorithm (for Affymetrix data). Individuals with ambiguous gender, high genotype missingness (>5%), excess heterozygosity (±4 s.d.) or non-Finnish ancestry were excluded, as well as all variants with high missingness (>2%), low Hardy–Weinberg equilibrium (*P* < 1 × 10^–6^) and minor allele count <3. Array data prephasing was carried out with Eagle 2.3.5 (ref. ^113^) with the number of conditioning haplotypes set at 20,000. Genotype imputation was done using the population-specific SISu v.3 imputation reference with 3,775 high-coverage (25–30×), whole-genome sequences in Finns, described in detail at 10.17504/protocols.io.xbgfijw.

We constructed PRSs from SNPs associated with the 127 biomarkers identified from MR analyses. To ensure comparability of the SNPs, we used only studies that included participants of European ancestry from the MR-Base database; in this database, data are harmonized. To ensure interoperability, PRSs were designed to be outcome specific by creating a separate PRS for each outcome from the pool of SNPs for biomarkers associated with the outcome of interest. SNPs were LD pruned, with clumping cutoff *R*^2^ = 0.01 and a 500-kb window with the R package TwoSampleMR. The final PRS contained only SNPs available in FinnGen genotypes. Final scores were determined with PLINK v.2.00aLM3, by calculating the SNP biomarker beta-weighted sum of risk alleles for each SNP. PRSs were scaled to zero mean and one-unit variance. Of the outcome-specific PRSs, we analyzed phenome-wide associations across 2,401 disease endpoints for Alzheimer’s disease PRS that was the only one with a sufficient number of SNPs available. For validity, we also applied a second genome-wide disease PRS for Alzheimer’s disease, generated with PRS–CS^114^ (PRS–CS-auto; LD reference panel 1000 G phase 3 European-ancestry individuals), using a recent large GWAS on Alzheimer’s disease by Jansen et al.^24^ as input. For sensitivity analyses excluding the *APOE* region, we calculated Jansen PRS and MR–PRS by excluding SNPs in positions 35,000,000–70,000,000 on chromosome 19 (GRCh38 in PRS and GRCh37 in MR–PRS). The association between PRS and endpoints was studied with logistic regression, adjusting for birth year, sex and the first ten principal components of ancestry.

We used IPW analyses to simulate RCTs on the effect of anti-inflammatory medication on risk of dementias in the observational FinnGen study^90^. These analyses included participants aged over 45 years and with no dementia-causing disease at baseline (*n* = 117,773). ATC codes for anti-inflammatory medication use were searched from medication purchase registry starting from 1995. To ensure powered analyses, we included only medications with at least ten users in participants who were diagnosed with dementia during follow-up. To simulate trial design and to avoid selection and immortal time bias, each analysis included only new medication users. In IPW analyses, we assumed that when medication is initiated, it is continued until the end of follow-up, to simulate intention-to-treat analyses and to provide conservative estimates. The baseline variables in IPW analyses were birth year, sex, ten principal components of ancestry and the following time-varying variables: statin, ACE-blocker, AT-blocker, renin-blocker, calcium channel blocker, any diuretic, insulin, metformin, other diabetes drug, depression medication, antipsychotic and anticoagulant use, as well as time varying any diagnoses of cancer, myocardial infarction, atrial fibrillation, heart failure, venous thromboembolism, ischemic stroke, intracerebral hemorrhage, subarachnoid hemorrhage, obesity, sleep apnea and chronic obstructive pulmonary disease, with informative censoring included. The positive control analyses on statin medication used these same variables but did not include statin as a time-varying covariate. In IPW analyses, PRSs were categorized into individuals above and below the median (PRS ≥ 50% and <50%). IPW analyses used the weighted FinnGen data to estimate the causal effect of each medication compared to no use of the medication studied. For an RCT that did not report *P* values, these were estimated using a method described by Altman and Bland^115^. *APOE* alleles in FinnGen were inferred based on genotype (rs7412 with minor allele frequency (MAF) 0.054 in Finns, INFO 0.997; rs429358, MAF 0.18, INFO 0.999). We used R (4.1.2) for these analyses.

### HLA analyses

These analyses were done in FinnGen using HLA alleles and imputed with high accuracy using a Finnish-specific reference panel, as previously described in detail^116^. After filtering based on an HLA carrier frequency of ≥0.01 and posterior probability of ≥0.6, we assessed the association between HLA alleles and dementias and autoimmune diseases using logistic regression adjusted for birth year, sex and the first ten principal components of ancestry.

Patients and control participants in FinnGen provided informed consent for biobank research, based on the Finnish Biobank Act. Separate research cohorts, with data collected before the Finnish Biobank Act came into effect (in September 2013) and before the start of FinnGen (August 2017), were based on study-specific consents and were later transferred to the Finnish biobanks after ethical approval by Fimea (Finnish Medicines Agency), the National Supervisory Authority for Welfare and Health. Recruitment protocols followed the biobank protocols approved by Fimea. The Coordinating Ethics Committee of the Hospital District of Helsinki and Uusimaa (HUS) statement number for the FinnGen study is HUS/990/2017.

### Open Targets analyses

Medications that changed the levels of the 127 biomarkers were searched in the Open Targets database (https://www.opentargets.org/) using UniProt protein names and Entrez gene symbols.

### Statistics and reproducibility

To study BBB- and immune system-related biology, biomarkers and drug targets for dementia-causing diseases, we conducted six separate studies, the designs and data of which are described in Fig. 1 and Table 1. No statistical methods were used to predetermine sample sizes; instead, these were determined based on available data. Study 1 used the freely available MR-Base GWAS catalog and MR to examine associations between a range of biomarkers and dementia-causing diseases. The details are described in Github^117^, and the data used in these analyses are provided in Zenodo^118^. Study 2 examined the pathways regulated by the biomarkers identified in study 1 using publicly available KEGG, ClueGO and ConsensusPathDB databases, Cytoscape and web-based analysis tools. Study 3 was an observational cohort study to investigate associations between plasma proteins and dementia in the Whitehall II cohort using Cox proportional-hazards models. Plasma proteins were available for 6,545 individuals (71% men) that participated in clinical screening between 1995 and 1997; 310 participants were excluded from the analyses due to missing data. Before the analyses, proteins were inverse rank based, normal transformed due to skewed distributions. None of the proteins violated proportionality assumptions of the Cox models. The analyses of study 3 are described in Github^117^. Studies 4, 5 and 6 were observational cohort studies and used the FinnGen dataset. Studies 4 and 5 used logistic regression to examine the associations of MR-Base polygenic risk score and HLA types with dementia-causing diseases. The participants included all 339,233 individuals (44% men) that were part of FinnGen Data Freeze 8; in studies 4 and 5, 0 and 24,788 participants, respectively, were excluded due to missing data. Study 6 used an IPW Cox proportional-hazards model to simulate RCTs on the effect of anti-inflammatory medications on risk of dementia-causing diseases. These analyses included 117,773 participants (55% men) aged over 45 years not treated with the medication investigated and without dementia-causing diseases at baseline. None of these analyses violated the assumptions of the IPW Cox proportional-hazards model. The analyses of studies 4, 5 and 6 are described in Github^117^. The R package versions used were data.table 1.14.2, dplyr 1.0.7, tidyr 1.1.4, survival 3.2.13, survminer 0.4.9, ggplot2 3.3.5 plyr 1.8.6, cluster 2.1.2, lubridate 1.8.0, stats 4.1.1, readxl 1.3.1, scales 1.1.1, tidyverse 1.3.1, Hmisc 4.6.0, devtools 2.4.2, TwoSampleMR 0.5.6, MRInstruments 0.3.2, ipw 1.0.11 and metafor 3.0.2. Other software used included ClueGO v.2.5.8 Cytoscape v.3.7.2, Cromwell 61, PLINK v.2.00aLM3, BCFtools 1.7 and 1.9, Eagle 2.3.5 and Beagle 4.1 (08Jun17.d8b).

### Ethics statement

In the Whitehall II study, research ethics approvals were renewed at each wave; the most recent approval was obtained from the University College London Hospital Committee on the Ethics of Human Research (reference no. 85/0938). Written, informed consent from participants was obtained at each contact. Patients and control subjects in FinnGen provided informed consent for biobank research, based on the Finnish Biobank Act. Alternatively, separate research cohorts, collected before the Finnish Biobank Act came into effect (in September 2013) and start of FinnGen (August 2017), were collected based on study-specific consents and later transferred to the Finnish biobanks after approval by Fimea, the National Supervisory Authority for Welfare and Health. Recruitment protocols followed the biobank protocols approved by Fimea. The Coordinating Ethics Committee of HUS statement number for the FinnGen study is HUS/990/2017. The FinnGen study is approved by Finnish Institute for Health and Welfare (permit nos. THL/2031/6.02.00/2017, THL/1101/5.05.00/2017, THL/341/6.02.00/2018, THL/2222/6.02.00/2018, THL/283/6.02.00/2019, THL/1721/5.05.00/2019 and THL/1524/5.05.00/2020), the Digital and population data service agency (permit nos. VRK43431/2017-3, VRK/6909/2018-3 and VRK/4415/2019-3), the Social Insurance Institution (permit nos. KELA 58/522/2017, KELA 131/522/2018, KELA 70/522/2019, KELA 98/522/2019, KELA 134/522/2019, KELA 138/522/2019, KELA 2/522/2020 and KELA 16/522/2020), Findata permit nos. THL/2364/14.02/2020, THL/4055/14.06.00/2020, THL/3433/14.06.00/2020, THL/4432/14.06/2020, THL/5189/14.06/2020, THL/5894/14.06.00/2020, THL/6619/14.06.00/2020, THL/209/14.06.00/2021, THL/688/14.06.00/2021, THL/1284/14.06.00/2021, THL/1965/14.06.00/2021, THL/5546/14.02.00/2020, THL/2658/14.06.00/2021 and THL/4235/14.06.00/2021 and Statistics Finland (permit nos. TK-53-1041-17, TK/143/07.03.00/2020 (previously TK-53-90-20) and TK/1735/07.03.00/2021). The Biobank Access Decisions for FinnGen samples and data utilized in FinnGen Data Freeze 8 include THL Biobank BB2017_55, BB2017_111, BB2018_19, BB_2018_34, BB_2018_67, BB2018_71, BB2019_7, BB2019_8, BB2019_26 and BB2020_1, Finnish Red Cross Blood Service Biobank 7.12.2017, Helsinki Biobank HUS/359/2017, Auria Biobank AB17-5154 and amendment no. 1 (17 August 2020) and AB20-5926 and amendment no. 1 (23 April 2020), Biobank Borealis of Northern Finland_2017_1013, Biobank of Eastern Finland 1186/2018 and amendment 22 § /2020, Finnish Clinical Biobank Tampere MH0004 and amendments (21.02.2020 and 06.10.2020), Central Finland Biobank 1-2017 and Terveystalo Biobank STB 2018001.

### Reporting summary

Further information on research design is available in the Nature Research Reporting Summary linked to this article.

## Supplementary information

Supplementary Information Supplementary Figs. 1 and 2, Tables 1–3 and FinnGen author list (not authors of this study).

Reporting Summary

Supplementary Data files 1–7 were deposited at Zenodo: https: //zenodo.org/deposit/7042008.

## Data Availability

This study used publicly available data at https://www.mrbase.org/, https://www.uniprot.org/, http://cpdb.molgen.mpg.de/, https://www.genome.jp/kegg/ and https://www.opentargets.org/. Data used in MR are deposited with Zenodo^118^ at https://zenodo.org/deposit/7042008. Data, protocols and other metadata of the Whitehall II and FinnGen studies are available according to the data-sharing policies of these studies. The pre-existing data access policy for the Whitehall II study specifies that research data requests can be submitted to the study steering committee, and these will be promptly reviewed for confidentiality or intellectual property restrictions and will not unreasonably be refused. Detailed information on data sharing can be found at https://www.ucl.ac.uk/epidemiology-health-care/research/epidemiology-and-public-health/research/whitehall-ii/data-sharing Individual-level patient or protein data may further be restricted by consent, confidentiality or privacy laws/considerations. FinnGen data can be accessed through Finnish Biobanks’ FinBB portal (www.finbb.fi). FinnGen summary statistics are freely available at https://www.finngen.fi/en/access_results, with results for new data freezes updated every 6 months.

## Code availability

Code used in this manuscript can be found at Github^117^: https://github.com/JVLind/Dementias_and_autoimmunity.
