# Supplementary material for: Immune system-wide Mendelian randomization and triangulation analyses support autoimmunity as a modifiable component in dementia-causing diseases
Source: Nat Aging. Author manuscript; Available in PMC 2023 May 8. (PMC10154235; doi:10.1038/s43587-022-00293-x)
Supplement: supplementary file [file NIHMS1883843-supplement-supplementary_file.pdf]

nature aging

Article

https://doi.org/10.1038/s43587-022-00293-x

Immune system-wide Mendelian randomization and triangulation analyses support autoimmunity as a modifiable component in dementia-causing diseases

In the format provided by the authors and unedited

Supplementary Information File

Supplementary Table 1. Odds ratios and 95% confidence intervals for significant backward Mendelian randomization results with p-value below 0.05. These odds ratios were not stronger than observed in forward Mendelian randomization analyses. The estimates are derived from Wald ratios when only one SNP was available and from inverse variance weighted Mendelian randomization when two or more SNPs were available. All the tests were two-sided.

Exposure	Outcome	Number of SNPs	OR (95% CIs)	p-value
Forward MR				
Alzheimer's disease	FSC-A on plasmacystoid dendritic cell	3	0.75 (0.65 to 0.86)	9.5e-5
Late-onset Alzheimer's disease	Protein S100-A13	1	0.36 (0.34 to 0.38)	9.9e-324
Backward MR				
Alzheimer's disease	FSC-A on plasmacystoid dendritic cell	20	0.85 (0.73 to 0.98)	0.0275
Late-onset Alzheimer's disease	Protein S100-A13	13	0.51 (0.41 to 0.63)	4.9e-10

Supplementary Figure 1. ClueGO enrichment analyses for all the 78 plasma proteins and cell receptors of the 127 biomarkers that had Uniprot ID and associated with dementia causing diseases in Mendelian randomization analyses. All proteins that associated with Uniprot search terms "immune system" and "blood brain barrier" were used as a background dataset. GO-terms in blue were enriched in cluster where most significant associations were observed for cell adhesion molecules. In the red cluster, the most significant GO-term associations were observed for hematopoietic cell lineage. Big dots describe the GO-terms and small dots the genes of the proteins associated with these terms.

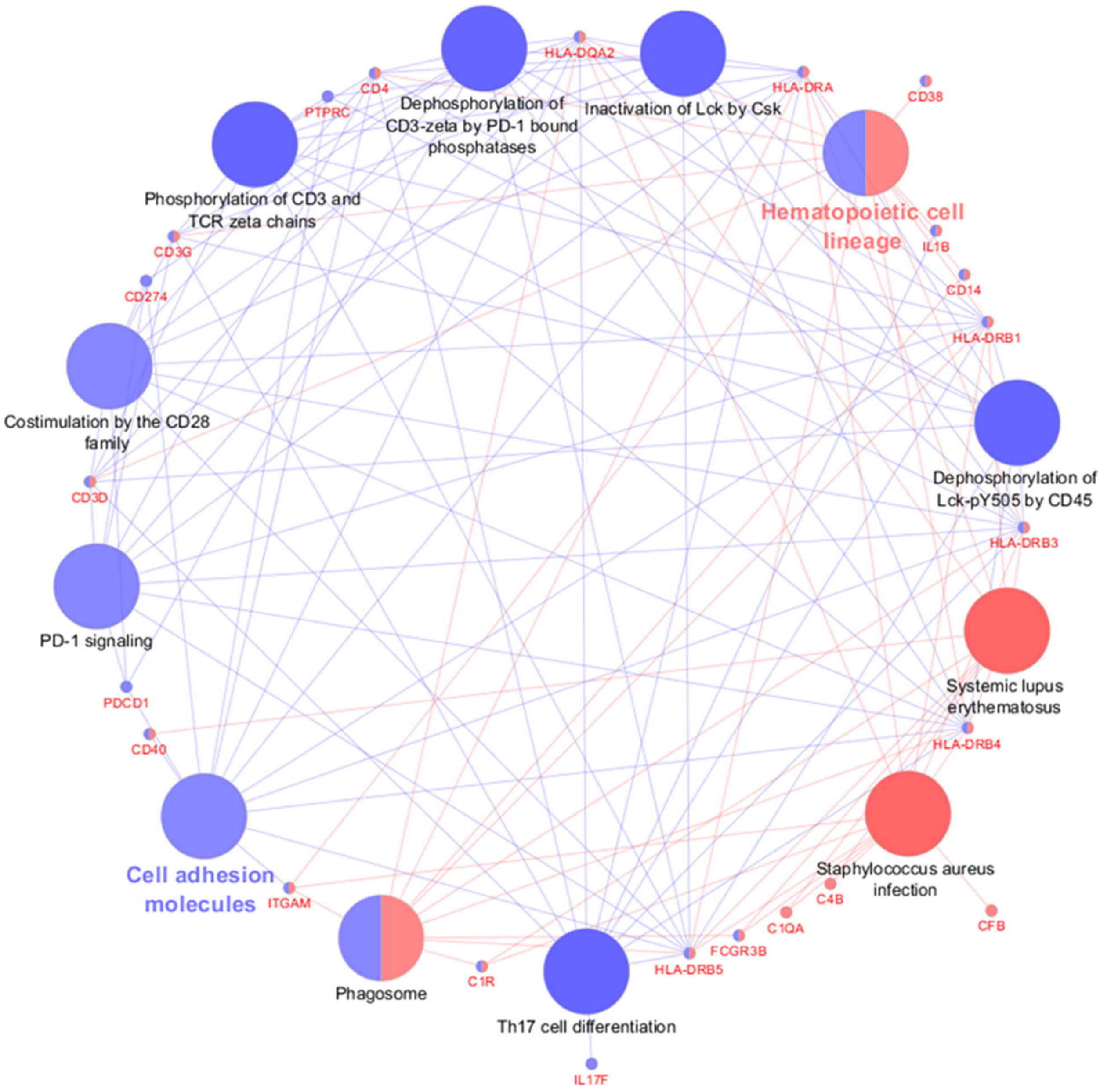

Supplementary Figure 2. KEGG functional pathway enrichment analyses for all the 78 plasma proteins and cell receptors of the 127 biomarkers that had Uniprot ID and associated with dementia causing diseases in Mendelian randomization analyses. The estimated causal betas were used in place of log fold change.

A)

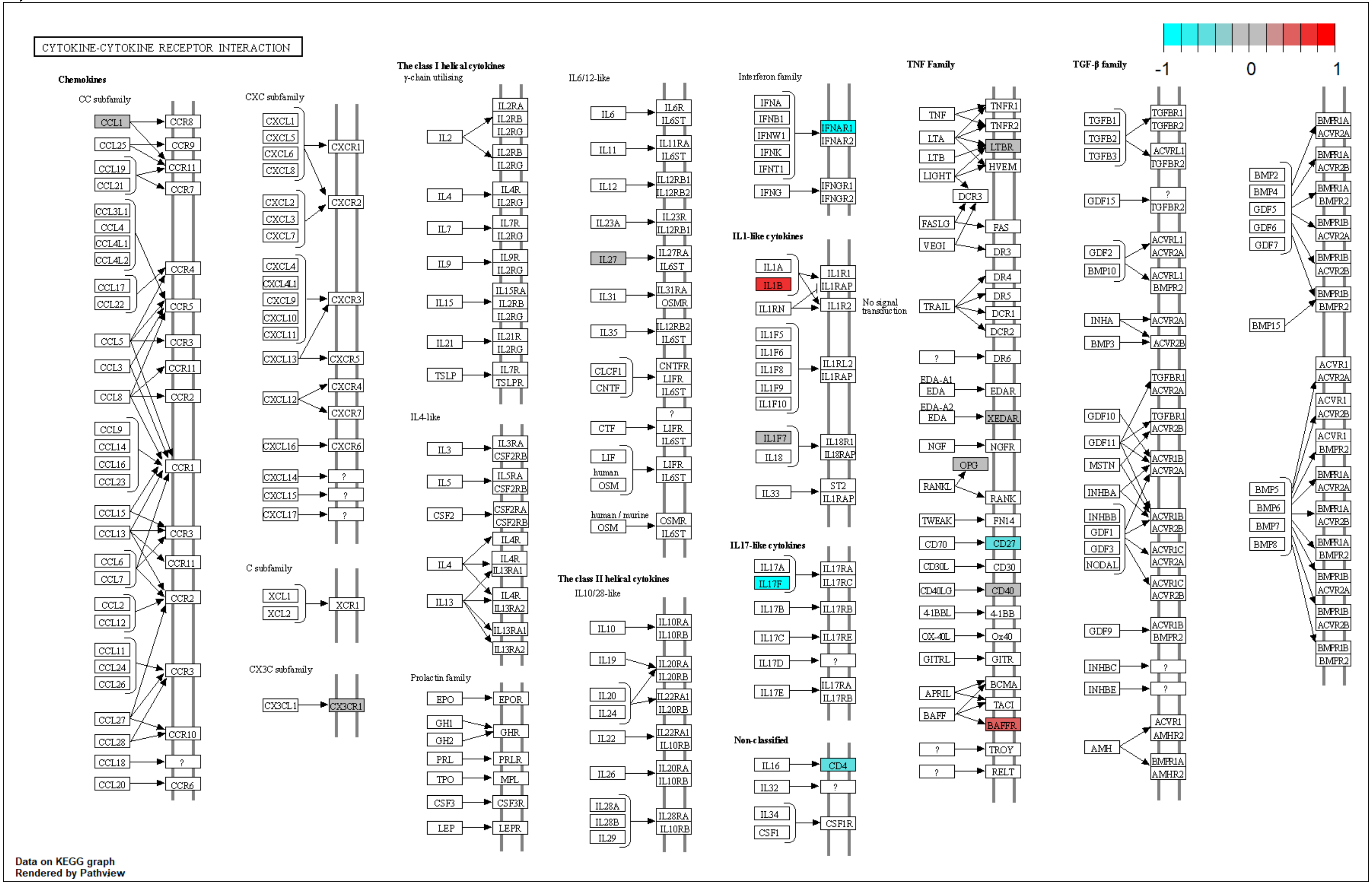
CYTOKINE-CYTOKINE RECEPTOR INTERACTION
-1
0
1
Chemokines
CC subfamily
CCL1
CCR8
CCL25
CCR9
CCL19
CCR11
CCL21
CCR7
CCL3L1
CCL4
CCL4L1
CCL4L2
CCR4
CCL17
CCL22
CCR5
CCL5
CCR3
CCL3
CCR11
CCL8
CCR2
CCL9
CCL14
CCL16
CCL23
CCR1
CCL15
CCL13
CCR3
CCL6
CCL7
CCR11
CCL2
CCL12
CCR2
CCL11
CCL24
CCL26
CCR3
CCL27
CCL28
CCR10
CCL18
?
CCL20
CCR6
CXC subfamily
CXCL1
CXCL5
CXCL6
CXCL8
CXCR1
CXCL2
CXCL3
CXCL7
CXCR2
CXCL4
CXCL4L1
CXCL9
CXCL10
CXCL11
CXCR3
CXCL13
CXCR5
CXCL12
CXCR4
CXCR7
CXCL16
CXCR6
CXCL14
?
CXCL15
?
CXCL17
?
C subfamily
XCL1
XCL2
XCR1
CX3C subfamily
CX3CL1
CX3CR1
The class I helical cytokines
γ-chain utilising
IL2
IL2RA
IL2RB
IL2RG
IL2RB
IL2RG
IL4
IL4R
IL2RG
IL7
IL7R
IL2RG
IL9
IL9R
IL2RG
IL15
IL15RA
IL2RB
IL2RG
IL21
IL21R
IL2RG
TSLP
IL7R
TSLPR
IL4-like
IL3
IL3RA
CSF2RB
IL5
IL5RA
CSF2RB
CSF2
CSF2RA
CSF2RB
IL4
IL4R
IL4R
IL13RA1
IL13
IL4R
IL13RA2
IL13RA1
IL13RA2
Prolactin family
EPO
EPOR
GH1
GH2
GHR
PRL
PRLR
TPO
MPL
CSF3
CSF3R
LEP
LEPR
IL6/12-like
IL6
IL6R
IL6ST
IL11
IL11RA
IL6ST
IL12
IL12RB1
IL12RB2
IL23A
IL23R
IL12RB1
IL27
IL27RA
IL6ST
IL31
IL31RA
OSMR
IL35
IL12RB2
IL6ST
CLCF1
CNTF
CNTFR
LIFR
IL6ST
?
CTF
LIFR
IL6ST
LIF
human
OSM
LIFR
IL6ST
human / murine
OSM
OSMR
IL6ST
The class II helical cytokines
IL10/28-like
IL10
IL10RA
IL10RB
IL19
IL20RA
IL20RB
IL20
IL24
IL22RA1
IL20RB
IL22
IL22RA1
IL10RB
IL26
IL20RA
IL10RB
IL28A
IL28B
IL29
IL28RA
IL10RB
Interferon family
IFNA
IFNB1
IFNW1
IFNK
IFNT1
IFNAR1
IFNAR2
IFNG
IFNGR1
IFNGR2
IL1-like cytokines
IL1A
IL1B
IL1R1
IL1RAP
IL1RN
IL1R2
No signal transduction
IL1F5
IL1F6
IL1F8
IL1F9
IL1F10
IL1RL2
IL1RAP
IL1F7
IL18
IL18R1
IL18RAP
IL33
ST2
IL1RAP
IL17-like cytokines
IL17A
IL17F
IL17RA
IL17RC
IL17B
IL17RB
IL17C
IL17RE
IL17D
?
IL17E
IL17RA
IL17RB
Non-classified
IL16
CD4
IL32
?
IL34
CSF1
CSF1R
TNF Family
TNF
TNFR1
TNFR2
LTA
LTB
LTBR
LIGHT
HVEM
DCR3
FASLG
FAS
VEGI
DR3
DR4
DR5
TRAIL
DCR1
DCR2
?
DR6
EDA-A1
EDA
EDAR
EDA-A2
EDA
XEDAR
NGF
NGFR
OPG
RANKL
RANK
TWEAK
FN14
CD70
CD27
CD30L
CD30
CD40LG
CD40
4-1BBL
4-1BB
OX-40L
Ox40
GITRL
GITR
APRIL
BCMA
BAFF
TACI
BAFFR
?
TROY
?
RELT
TGF-β family
TGFB1
TGFB2
TGFB3
TGFBR1
TGFBR2
ACVRL1
TGFBR2
GDF15
?
TGFBR2
GDF2
BMP10
ACVRL1
ACVR2A
ACVRL1
BMPR2
INHA
BMP3
ACVR2A
ACVR2B
GDF10
GDF11
MSTN
INHBA
INHBB
GDF1
GDF3
NODAL
TGFBR1
ACVR2A
TGFBR1
ACVR2B
ACVR1B
ACVR2A
ACVR1B
ACVR2B
ACVR1C
ACVR2A
ACVR1C
ACVR2B
GDF9
ACVR1B
BMPR2
INHBC
?
INHBE
?
AMH
ACVR1
AMHR2
BMPR1A
AMHR2
BMP2
BMP4
GDF5
GDF6
GDF7
BMPR1A
ACVR2A
BMPR1A
ACVR2B
BMPR1A
BMPR2
BMPR1B
ACVR2A
BMPR1B
ACVR2B
BMPR1B
BMPR2
BMP15
BMP5
BMP6
BMP7
BMP8
ACVR1
ACVR2A
ACVR1
ACVR2B
ACVR1
BMPR2
BMPR1A
ACVR2A
BMPR1A
ACVR2B
BMPR1A
BMPR2
BMPR1B
ACVR2A
BMPR1B
ACVR2B
BMPR1B
BMPR2
Data on KEGG graph
Rendered by Pathview

B)

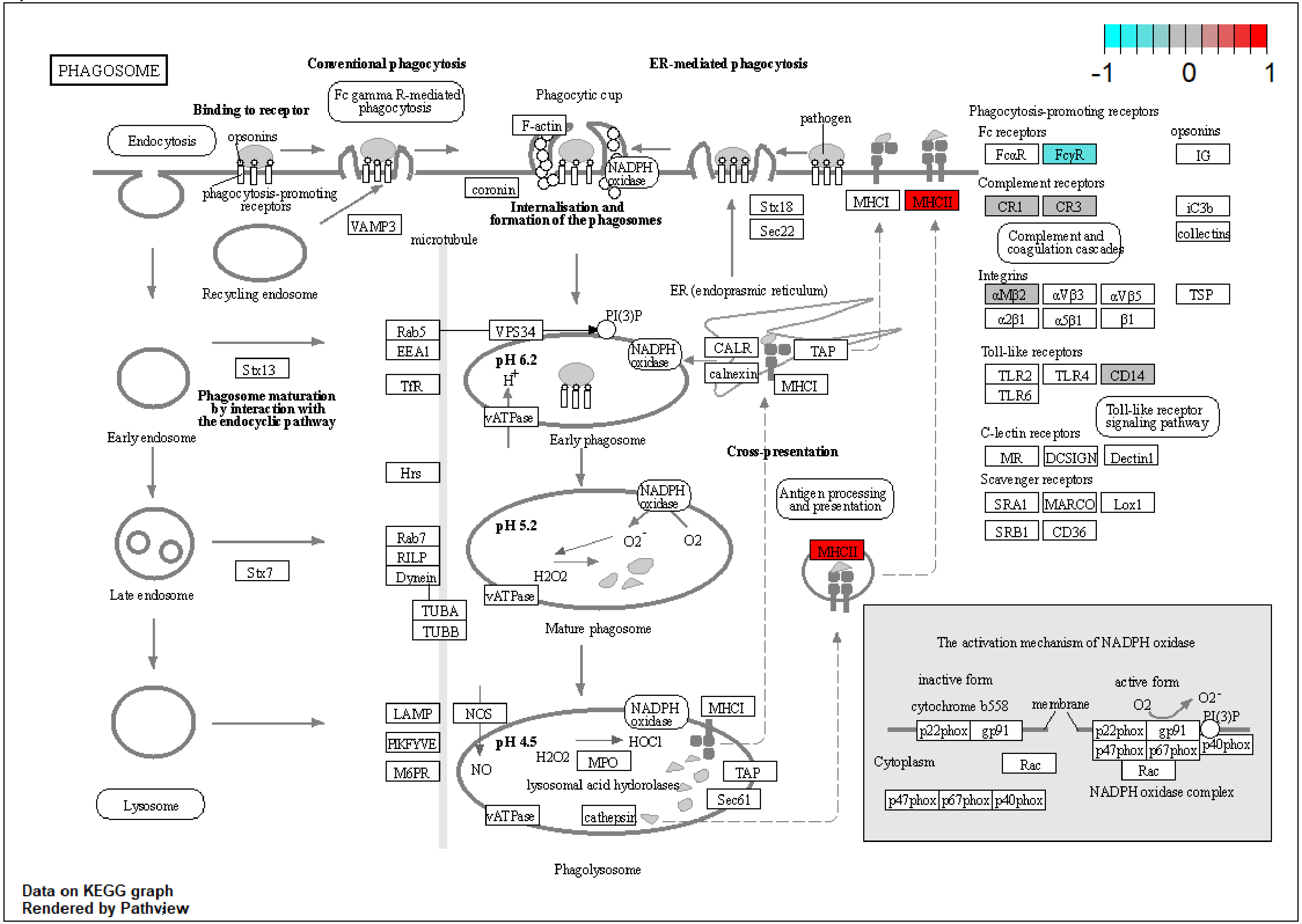
PHAGOSOME
-1
0
1
Conventional phagocytosis
ER-mediated phagocytosis
Binding to receptor
Fc gamma R-mediated phagocytosis
Phagocytic cup
pathogen
Endocytosis
opsonins
phagocytosis-promoting receptors
F-actin
NADPH oxidase
coronin
VAMP3
microtubule
Internalisation and formation of the phagosomes
Stx18
Sec22
MHCI
MHCII
Recycling endosome
ER (endoplasmic reticulum)
Rab5
EEA1
VPS34
PI(3)P
pH 6.2
H+
vATPase
TfR
Stx13
Phagosome maturation by interaction with the endocytic pathway
Early endosome
Early phagosome
CALR
calnexin
TAP
Cross-presentation
Hrs
Antigen processing and presentation
pH 5.2
O2-
O2
H2O2
Rab7
RILP
Dynein
TUBA
TUBB
Stx7
Late endosome
Mature phagosome
LAMP
NOS
PIKFYVE
M6PR
pH 4.5
HOCl
MPO
NO
lysosomal acid hydrolases
TAP
Sec61
cathepsin
Lysosome
Phagolysosome
Phagocytosis-promoting receptors
Fc receptors
FcαR
FcγR
opsonins
IG
Complement receptors
CR1
CR3
iC3b
collectins
Complement and coagulation cascades
Integrins
αMβ2
αVβ3
αVβ5
TSP
α2β1
α5β1
β1
Toll-like receptors
TLR2
TLR4
CD14
TLR6
Toll-like receptor signaling pathway
C-lectin receptors
MR
DCSIGN
Dectin1
Scavenger receptors
SRA1
MARCO
Lox1
SRB1
CD36
The activation mechanism of NADPH oxidase
inactive form
active form
cytochrome b558
membrane
O2
O2-
PI(3)P
p22phox
gp91
p40phox
p47phox
p67phox
Cytoplasm
Rac
NADPH oxidase complex
Data on KEGG graph
Rendered by Pathview

C)

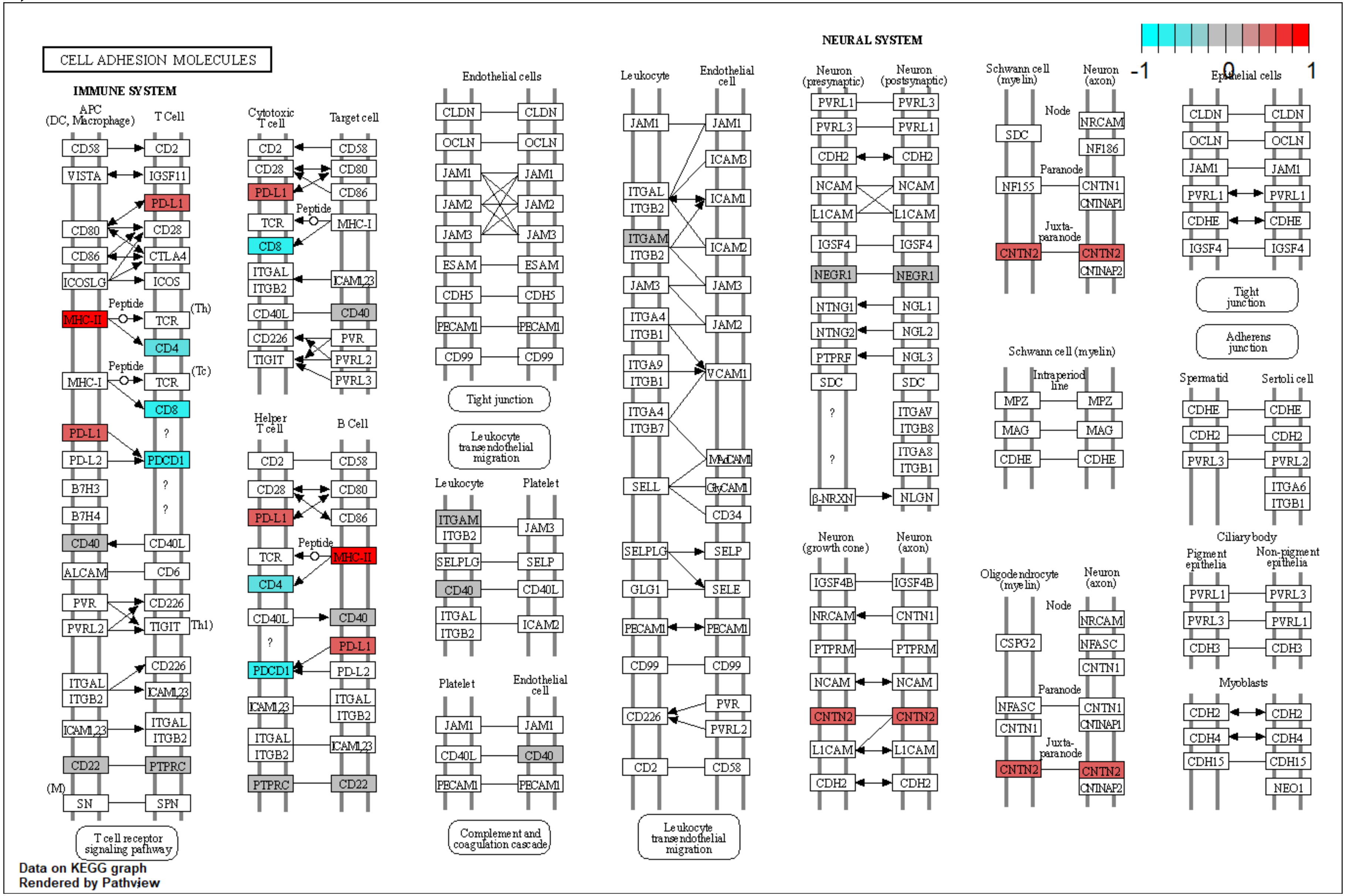
CELL ADHESION MOLECULES
IMMUNE SYSTEM
NEURAL SYSTEM
-1
0
1
APC (DC, Macrophage)
T Cell
CD58
CD2
VISTA
IGSF11
PD-L1
CD80
CD28
CD86
CTLA4
ICOSLG
ICOS
Peptide
MHC-II
TCR
(Th)
CD4
MHC-I
(Tc)
CD8
PD-L2
PDCD1
B7H3
B7H4
CD40
CD40L
ALCAM
CD6
PVR
CD226
PVRL2
TIGIT
Th1)
ITGAL
ITGB2
ICAM1,2,3
CD22
PTPRC
(M)
SN
SPN
T cell receptor signaling pathway
Cytotoxic T cell
Target cell
MHC-I
ICAM1,2,3
PVRL3
Helper T cell
B Cell
Endothelial cells
CLDN
OCLN
JAM1
JAM2
JAM3
ESAM
CDH5
PECAM1
CD99
Tight junction
Leukocyte transendothelial migration
Leukocyte
Platelet
ITGAM
SELPLG
SELP
ICAM2
Complement and coagulation cascade
Endothelial cell
ICAM3
ICAM1
ITGA4
ITGB1
ITGA9
VCAM1
ITGB7
MAdCAM1
SELL
GlyCAM1
CD34
SELE
GLG1
Neuron (presynaptic)
Neuron (postsynaptic)
PVRL1
CDH2
NCAM
L1CAM
IGSF4
NEGR1
NTNG1
NGL1
NTNG2
NGL2
PTPRF
NGL3
SDC
ITGAV
ITGB8
ITGA8
β-NRXN
NLGN
?
Neuron (growth cone)
Neuron (axon)
IGSF4B
NRCAM
CNTN1
PTPRM
CNTN2
Schwann cell (myelin)
Node
NF186
Paranode
NF155
CNTNAP1
Juxta-paranode
CNTNAP2
Intraperiod line
MPZ
MAG
CDHE
Oligodendrocyte (myelin)
CSPG2
NFASC
Epithelial cells
Adherens junction
Spermatid
Sertoli cell
ITGA6
Ciliary body
Pigment epithelia
Non-pigment epithelia
CDH3
Myoblasts
CDH4
CDH15
NEO1
Data on KEGG graph
Rendered by Pathview

D)

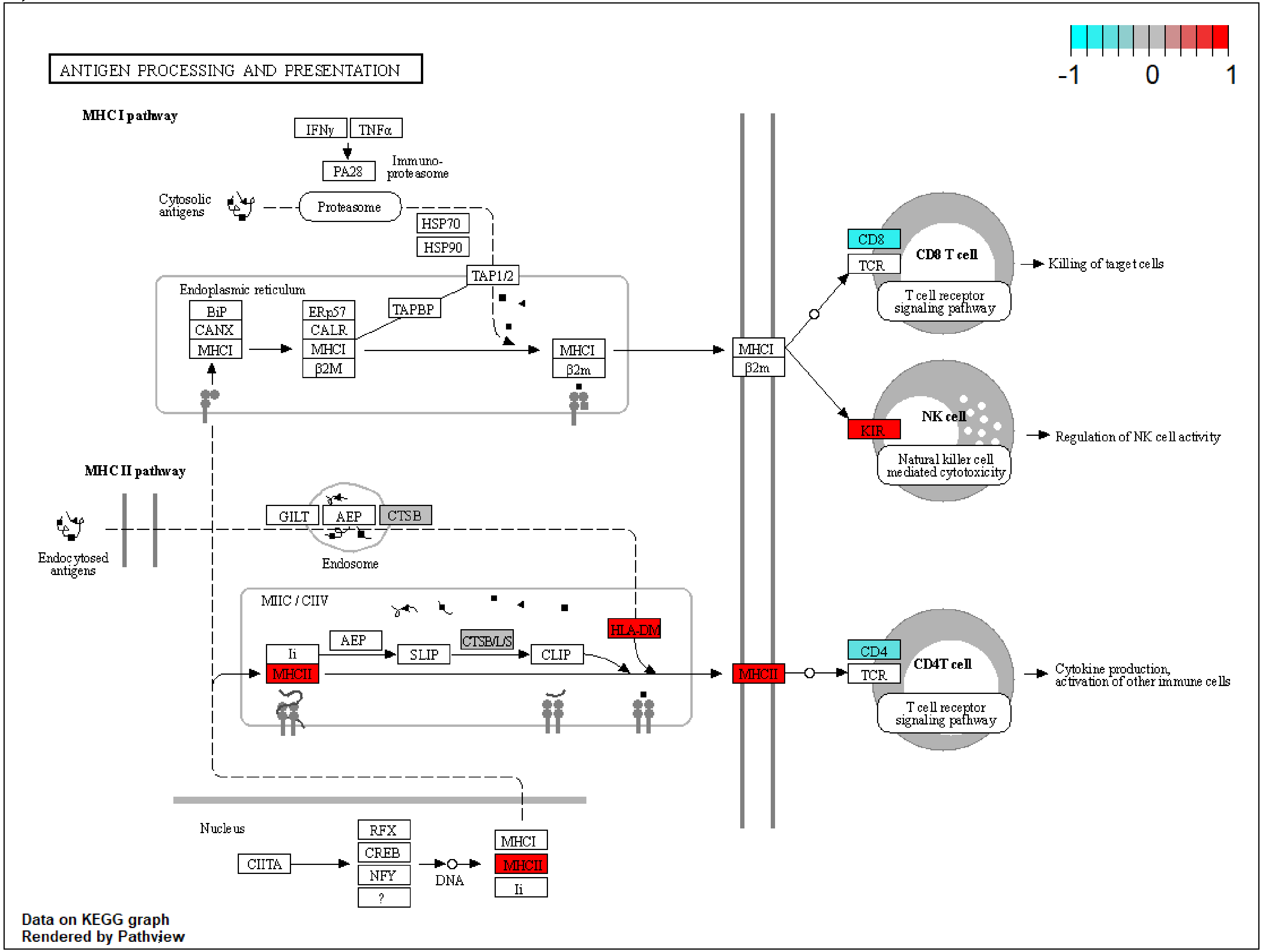
ANTIGEN PROCESSING AND PRESENTATION
-1
0
1
MHC I pathway
IFNγ
TNFα
PA28
Immuno-proteasome
Cytosolic antigens
Proteasome
HSP70
HSP90
TAP1/2
Endoplasmic reticulum
BiP
CANX
MHCI
ERp57
CALR
MHCI
β2M
TAPBP
MHCI
β2m
MHCI
β2m
CD8
TCR
CD8 T cell
T cell receptor signaling pathway
Killing of target cells
KIR
NK cell
Natural killer cell mediated cytotoxicity
Regulation of NK cell activity
MHC II pathway
Endocytosed antigens
GILT
AEP
CTSB
Endosome
MIIC / CIIV
AEP
CTSB/L/S
HLA-DM
Ii
MHCII
SLIP
CLIP
MHCII
CD4
TCR
CD4T cell
T cell receptor signaling pathway
Cytokine production, activation of other immune cells
Nucleus
CIITA
RFX
CREB
NFY
?
DNA
MHCI
MHCII
Ii
Data on KEGG graph
Rendered by Pathview

E)

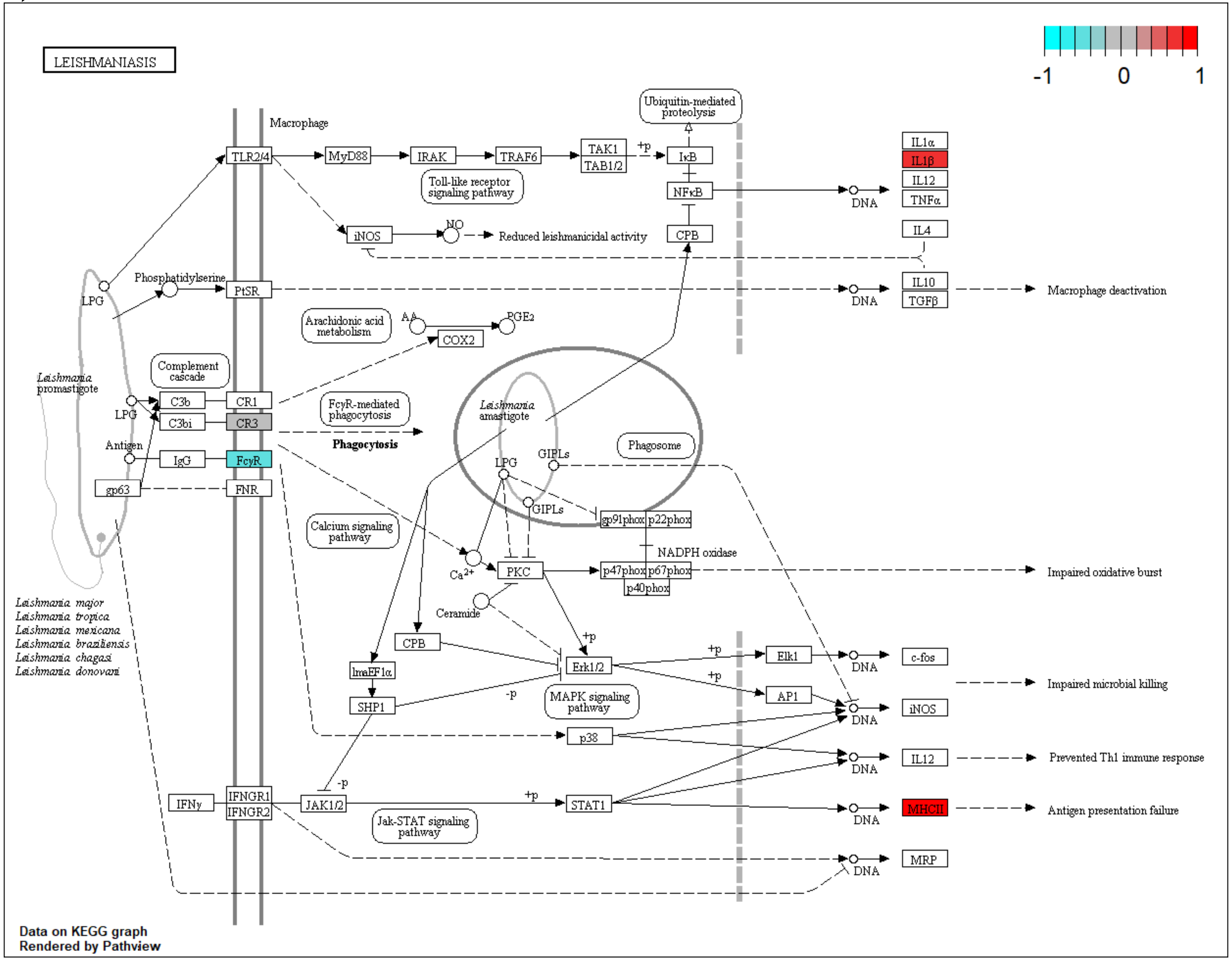

LEISHMANIASIS
-1
0
1
Macrophage
TLR2/4
MyD88
IRAK
TRAF6
TAK1
TAB1/2
+p
IκB
NFκB
CPB
Ubiquitin-mediated proteolysis
Toll-like receptor signaling pathway
IL1α
IL1β
IL12
TNFα
DNA
iNOS
NO
Reduced leishmanicidal activity
IL4
IL10
TGFβ
Macrophage deactivation
LPG
Phosphatidylserine
PtSR
Arachidonic acid metabolism
AA
PGE2
COX2
Complement cascade
Leishmania promastigote
C3b
C3bi
CR1
CR3
FcγR-mediated phagocytosis
Phagocytosis
Antigen
IgG
FcγR
gp63
FNR
Leishmania amastigote
Phagosome
GIPLs
gp91phox
p22phox
NADPH oxidase
Calcium signaling pathway
Ca2+
PKC
p47phox
p67phox
p40phox
Impaired oxidative burst
Ceramide
Leishmania major
Leishmania tropica
Leishmania mexicana
Leishmania braziliensis
Leishmania chagasi
Leishmania donovani
CPB
lmaEF1α
SHP1
-p
Erk1/2
Elk1
AP1
c-fos
Impaired microbial killing
MAPK signaling pathway
p38
IL12
Prevented Th1 immune response
IFNγ
IFNGR1
IFNGR2
JAK1/2
Jak-STAT signaling pathway
STAT1
MHCII
Antigen presentation failure
MRP
Data on KEGG graph
Rendered by Pathview

F)

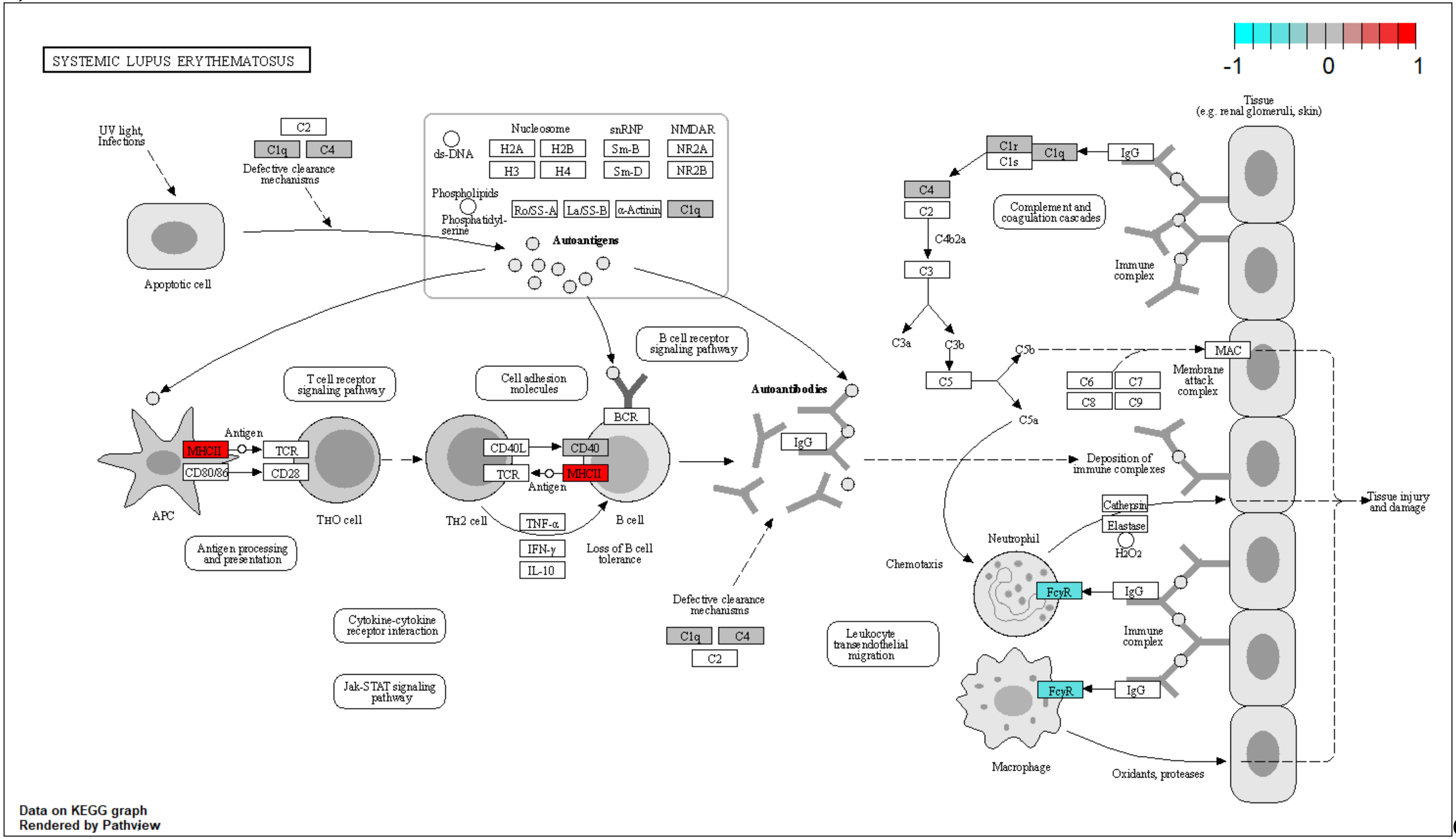
SYSTEMIC LUPUS ERYTHEMATOSUS
-1
0
1
UV light, Infections
C2
C1q
C4
Defective clearance mechanisms
Apoptotic cell
ds-DNA
Nucleosome
H2A
H2B
H3
H4
snRNP
Sm-B
Sm-D
NMDAR
NR2A
NR2B
Phospholipids
Phosphatidyl-serine
Ro/SS-A
La/SS-B
α-Actinin
C1q
Autoantigens
Tissue (e.g. renal glomeruli, skin)
C1r
C1s
C1q
IgG
C4
C2
C4b2a
C3
C3a
C3b
C5
C5b
C5a
Complement and coagulation cascades
Immune complex
MAC
Membrane attack complex
C6
C7
C8
C9
B cell receptor signaling pathway
T cell receptor signaling pathway
Cell adhesion molecules
Antigen
MHCII
TCR
CD80/86
CD28
APC
THO cell
CD40L
CD40
TCR
MHCII
Antigen
TH2 cell
BCR
B cell
TNF-α
IFN-γ
IL-10
Loss of B cell tolerance
Autoantibodies
IgG
Deposition of immune complexes
Tissue injury and damage
Cathepsin
Elastase
H2O2
Neutrophil
Chemotaxis
FcγR
IgG
Immune complex
FcγR
IgG
Macrophage
Oxidants, proteases
Antigen processing and presentation
Cytokine-cytokine receptor interaction
Jak-STAT signaling pathway
Defective clearance mechanisms
C1q
C4
C2
Leukocyte transendothelial migration
Data on KEGG graph
Rendered by Pathview

G)

G)

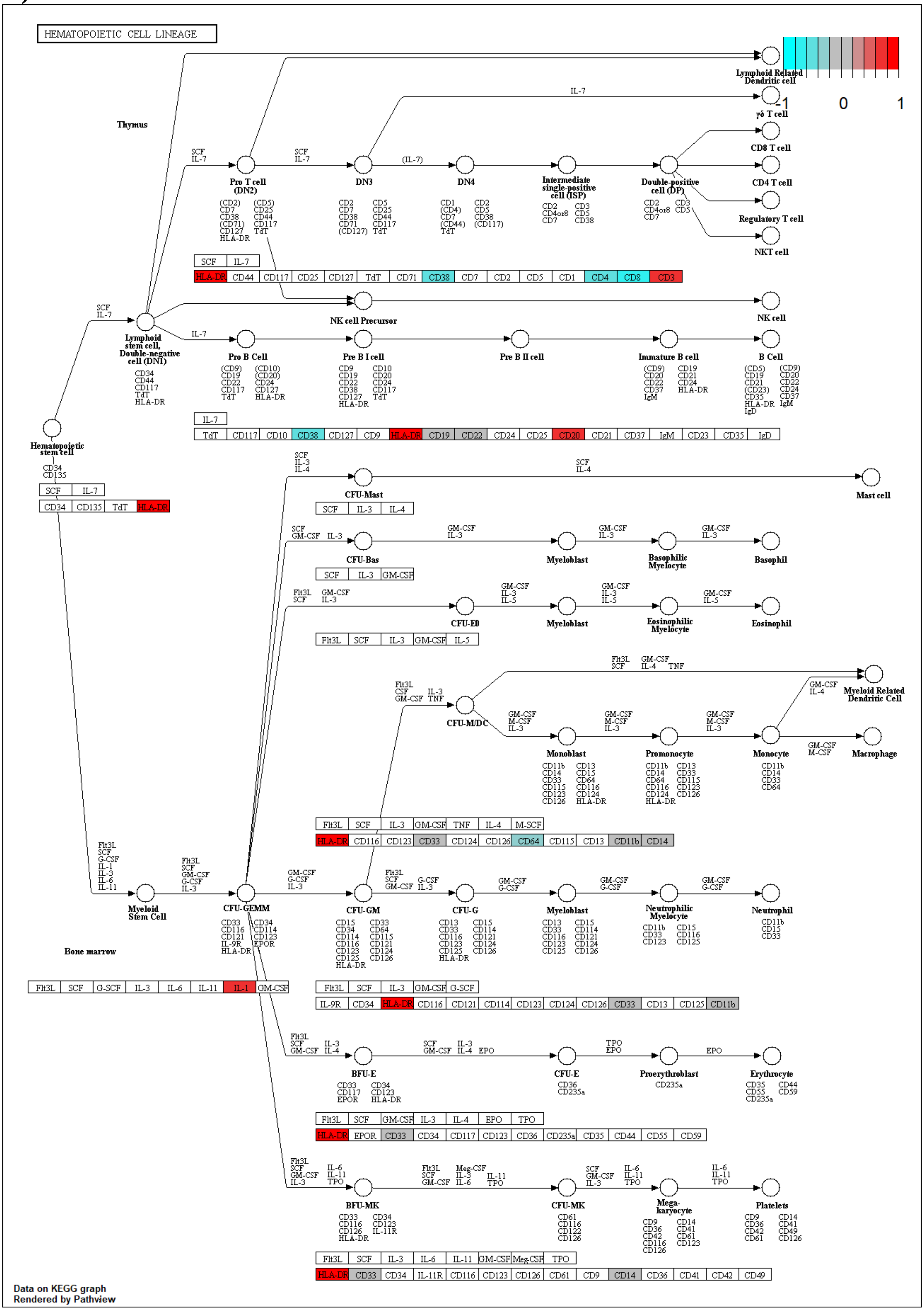

HEMATOPOIETIC CELL LINEAGE
-1
0
1
Thymus
Lymphoid Related Dendritic cell
γδ T cell
CD8 T cell
CD4 T cell
Regulatory T cell
NKT cell
Pro T cell (DN2)
DN3
DN4
Intermediate single-positive cell (ISP)
Double-positive cell (DP)
SCF IL-7
(IL-7)
IL-7
SCF
IL-7
HLA-DR
CD44
CD117
CD25
CD127
TdT
CD71
CD38
CD7
CD2
CD5
CD1
CD4
CD8
CD3
NK cell Precursor
NK cell
Lymphoid stem cell, Double-negative cell (DN1)
Pro B Cell
Pre B I cell
Pre B II cell
Immature B cell
B Cell
TdT
CD117
CD10
CD38
CD127
CD9
HLA-DR
CD19
CD22
CD24
CD25
CD20
CD21
CD37
IgM
CD23
CD35
IgD
Hematopoietic stem cell
CD34 CD135
SCF
IL-7
CD34
CD135
TdT
HLA-DR
CFU-Mast
Mast cell
SCF
IL-3
IL-4
CFU-Bas
Myeloblast
Basophilic Myelocyte
Basophil
SCF
IL-3
GM-CSF
CFU-EO
Myeloblast
Eosinophilic Myelocyte
Eosinophil
Flt3L
SCF
IL-3
GM-CSF
IL-5
CFU-M/DC
Myeloid Related Dendritic Cell
Monoblast
Promonocyte
Monocyte
Macrophage
Flt3L
SCF
IL-3
GM-CSF
TNF
IL-4
M-SCF
HLA-DR
CD116
CD123
CD33
CD124
CD126
CD64
CD115
CD13
CD11b
CD14
Myeloid Stem Cell
CFU-GEMM
CFU-GM
CFU-G
Myeloblast
Neutrophilic Myelocyte
Neutrophil
Bone marrow
Flt3L
SCF
G-SCF
IL-3
IL-6
IL-11
IL-1
GM-CSF
Flt3L
SCF
IL-3
GM-CSF
G-SCF
IL-9R
CD34
HLA-DR
CD116
CD121
CD114
CD123
CD124
CD126
CD33
CD13
CD125
CD11b
BFU-E
CFU-E
Proerythroblast
Erythrocyte
Flt3L
SCF
GM-CSF
IL-3
IL-4
EPO
TPO
HLA-DR
EPOR
CD33
CD34
CD117
CD123
CD36
CD235a
CD35
CD44
CD55
CD59
BFU-MK
CFU-MK
Mega-karyocyte
Platelets
Flt3L
SCF
IL-3
IL-6
IL-11
GM-CSF
Meg-CSF
TPO
HLA-DR
CD33
CD34
IL-11R
CD116
CD123
CD126
CD61
CD9
CD14
CD36
CD41
CD42
CD49
Data on KEGG graph
Rendered by Pathview

H)

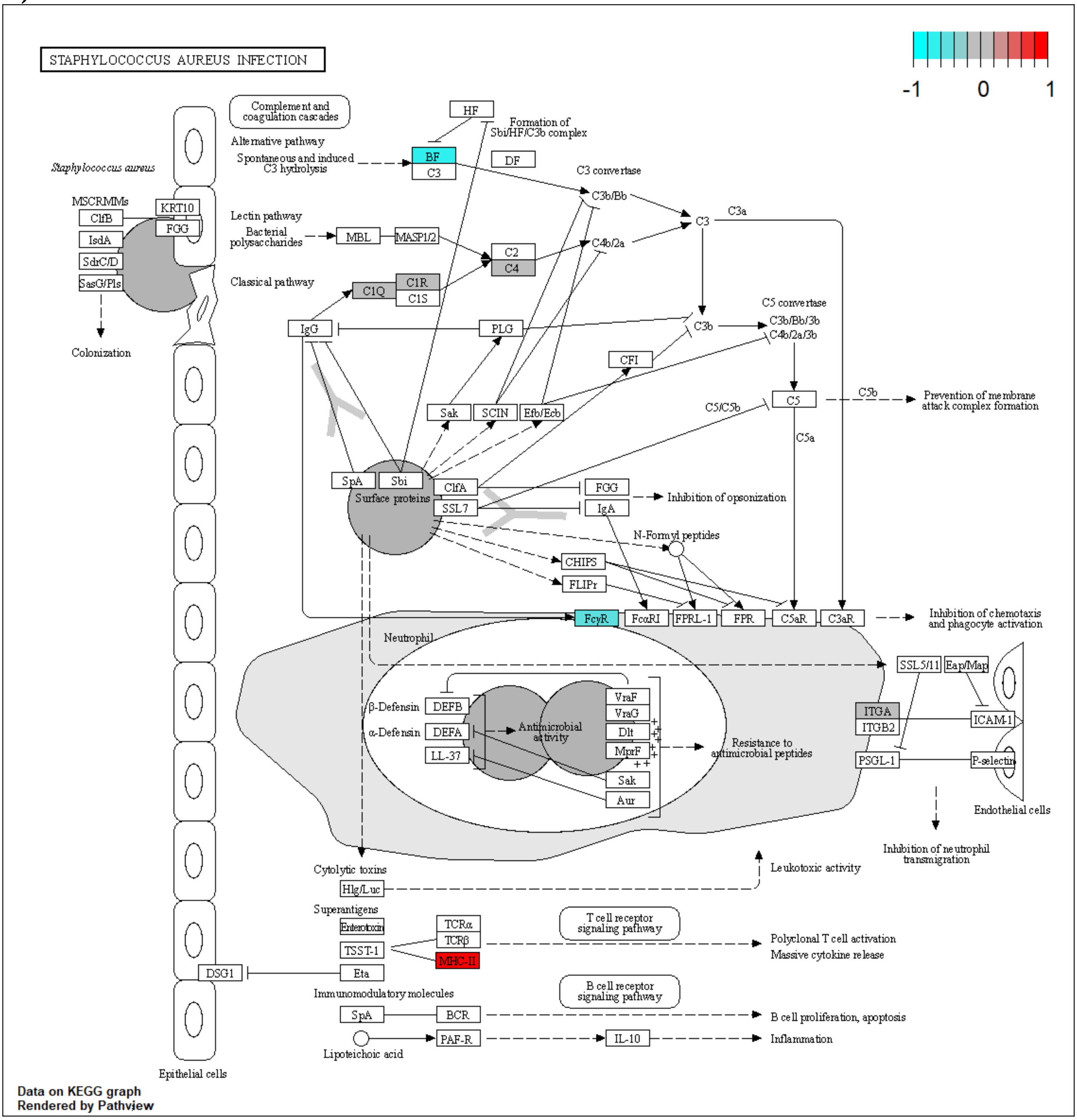
STAPHYLOCOCCUS AUREUS INFECTION
-1
0
1
Complement and coagulation cascades
Alternative pathway
Spontaneous and induced C3 hydrolysis
HF
BF
C3
DF
Formation of Sbi/HF/C3b complex
C3 convertase
C3b/Bb
C4b/2a
C3
C3a
Staphylococcus aureus
MSCRMMs
ClfB
IsdA
SdrC/D
SasG/Pls
KRT10
FGG
Colonization
Lectin pathway
Bacterial polysaccharides
MBL
MASP1/2
C2
C4
Classical pathway
C1Q
C1R
C1S
IgG
PLG
C3b
C5 convertase
C3b/Bb/3b
C4b/2a/3b
CFI
Sak
SCIN
Efb/Ecb
C5/C5b
C5
C5b
C5a
Prevention of membrane attack complex formation
SpA
Sbi
Surface proteins
ClfA
SSL7
FGG
IgA
Inhibition of opsonization
N-Formyl peptides
CHIPS
FLIPr
FcγR
FcαRI
FPRL-1
FPR
C5aR
C3aR
Inhibition of chemotaxis and phagocyte activation
Neutrophil
SSL5/11
Eap/Map
β-Defensin
DEFB
α-Defensin
DEFA
LL-37
Antimicrobial activity
VraF
VraG
Dlt
MprF
Sak
Aur
Resistance to antimicrobial peptides
ITGA
ITGB2
PSGL-1
ICAM-1
P-selectin
Endothelial cells
Inhibition of neutrophil transmigration
Cytolytic toxins
Hlg/Luc
Leukotoxic activity
Superantigens
Enterotoxin
TSST-1
TCRα
TCRβ
MHC-II
T cell receptor signaling pathway
Polyclonal T cell activation
Massive cytokine release
DSG1
Eta
Immunomodulatory molecules
SpA
BCR
B cell receptor signaling pathway
B cell proliferation, apoptosis
PAF-R
IL-10
Inflammation
Lipoteichoic acid
Epithelial cells
Data on KEGG graph
Rendered by Pathview

Supplementary Table 2. Associations between the main PheWAS results and the Mendelian randomization polygenic risk score when *APOE* region is excluded. The Mendelian randomization polygenic risk score was derived from SNPs that change the levels of the biomarkers associated with Alzheimer's disease in Mendelian randomization but are not located in *APOE* region. All the tests were two-sided.

Outcome	HR and 95% CIs per SD	p-value
Alzheimer´s disease	1.00 (0.98 to 1.02)	0.7013
Type 1 diabetes	1.72 (1.68 to 1.76)	9.9e-324
Seropositive rheumatoid arthritis	1.45 (1.40 to 1.49)	4.4e-122

Supplementary Table 3. Hazard ratios and 95% confidence intervals for associations between antihistamine, corticosteroids, non-steroidal anti-inflammatory drugs, salazine medications and dementia causing diseases from inverse probability weighted (IPW) Cox proportional hazards survival analyses in the FinnGen study. The positive control analyses in Table 3 validate the IPW analysis protocol by replicating the established association between statin medication and reduced coronary heart disease risk. As a further validation step, the negative control IPW analyses replicate the null finding between anti-inflammatory medications and coronary heart disease. In the IPW analyses, the baseline variables were birth year, sex, ten principal components and time-varying variables statin, ACE-blocker, AT-blocker, renin-blocker, calcium channel blocker, any diuretic, insulin, metformin, other diabetes drug, antidepressant, antipsychotic, and anticoagulant medication use; as well as time varying disease diagnosis (any cancer, myocardial infarction, atrial fibrillation, heart failure, venous thromboembolism, ischemic stroke, intracerebral haemorrhage, subarachnoid haemorrhage, obesity, sleep apnoea, and chronic obstructive pulmonary diseases), with informative censoring included. Analyses include only medication-free individuals at baseline. This analysis was not corrected for multiple testing and all the tests were two-sided. The estimate for randomized control trial is from reference 21. MR-PRS, Mendelian randomization based polygenic risk score for Alzheimer's disease; NSAID, non-steroidal anti-inflammatory drugs; IPW, inverse probability weighted analyses; RCT, randomized control trials.

Medication	Outcome	HR and 95% CIs per SD	p-value
NSAIDs	Alzheimer´s disease	0.37 (0.35 to 0.40)	1.9e-152
	Vascular dementia	0.25 (0.20 to 0.32)	1.2e-33
	Parkinson´s disease	0.65 (0.57 to 0.75)	1.3e-9
Negative control	Coronary heart disease IPW	0.38 (0.34 to 0.41)	4.8e-118
	Coronary heart disease RCT	1.48 (1.00 to 2.26)	0.059
Antihistamines	Alzheimer´s disease	0.54 (0.48 to 0.61)	8.6e-22
	Vascular dementia	0.43 (0.29 to 0.64)	3.1e-5
	Parkinson´s disease	0.70 (0.56 to 0.89)	0.003
Negative control	Coronary heart disease IPW	0.59 (0.51 to 0.69)	0.009
	Coronary heart disease RCT	No RCT available	
Corticosteroids	Alzheimer´s disease	0.65 (0.40 to 1.06)	0.085
	Vascular dementia	1.02 (0.32 to 3.21)	0.972
	Parkinson´s disease	0.50 (0.12 to 2.18)	0.358
Negative control	Coronary heart disease IPW	0.55 (0.25 to 1.23)	0.556
	Coronary heart disease RCT	No RCT available	
Salazines	Alzheimer´s disease	0.89 (0.71 to 1.11)	0.296
	Vascular dementia	0.82 (0.41 to 1.66)	0.583
	Parkinson´s disease	0.69 (0.42 to 1.11)	0.128
Negative control	Coronary heart disease IPW	1.06 (0.89 to 1.27)	0.490
	Coronary heart disease RCT	No RCT available	

FinnGen

Full Name	Affiliation	E-mail	Role 1	Role 2
Aarno Palotie	Institute for Molecular Medicine Finland (FIMM), HiLIFE, University of Helsinki, Helsinki,	aarno.palotie@helsinki.fi	Steering Committee	Steering Committee
Mark Daly	Institute for Molecular Medicine Finland (FIMM), HiLIFE, University of Helsinki, Helsinki,	mark.daly@helsinki.fi	Steering Committee	Steering Committee
Bridget Riley-Gills	Abbvie, Chicago, IL, United States	bridget.rileygillis@abbvie.com	Steering Committee	Pharmaceutical companies
Howard Jacob	Abbvie, Chicago, IL, United States	howard.jacob@abbvie.com	Steering Committee	Pharmaceutical companies
Dirk Paul	Astra Zeneca, Cambridge, United Kingdom	dirk.paul@astrazeneca.com	Steering Committee	Pharmaceutical companies
Athena Matakidou	Astra Zeneca, Cambridge, United Kingdom	athena.x.matakidou@gsk.com	Steering Committee	Pharmaceutical companies
Adam Platt	Astra Zeneca, Cambridge, United Kingdom	adam.platt@astrazeneca.com	Steering Committee	Pharmaceutical companies
Heiko Runz	Biogen, Cambridge, MA, United States	heiko.runz@biogen.com	Steering Committee	Pharmaceutical companies
Sally John	Biogen, Cambridge, MA, United States	sally.john@biogen.com	Steering Committee	Pharmaceutical companies
George Okafo	Boehringer Ingelheim, Ingelheim am Rhein, Germany	george.okafo@boehringer-ingelheim.com	Steering Committee	Pharmaceutical companies
Nathan Lawless	Boehringer Ingelheim, Ingelheim am Rhein, Germany	nathan.lawless@boehringer-ingelheim.com	Steering Committee	Pharmaceutical companies
Robert Plenge	Bristol Myers Squibb, New York, NY, United States	robert.plenge@bms.com	Steering Committee	Pharmaceutical companies
Joseph Maranville	Bristol Myers Squibb, New York, NY, United States	joseph.maranville@bms.com	Steering Committee	Pharmaceutical companies
Mark McCarthy	Genentech, San Francisco, CA, United States	mccarthy.mark@gene.com	Steering Committee	Pharmaceutical companies
Julie Hunkapiller	Genentech, San Francisco, CA, United States	hunkapiller.julie@gene.com	Steering Committee	Pharmaceutical companies
Margaret G. Ehm	GlaxoSmithKline, Collegeville, PA, United States	meg.g.ehm@gsk.com	Steering Committee	Pharmaceutical companies
Kirsi Auro	GlaxoSmithKline, Espoo, Finland	kirsi.m.auro@gsk.com	Steering Committee	Pharmaceutical companies
Simonne Longerich	Merck, Kenilworth, NJ, United States	simonne.longerich@merck.com	Steering Committee	Pharmaceutical companies
Caroline Fox	Merck, Kenilworth, NJ, United States	caroline.fox@merck.com	Steering Committee	Pharmaceutical companies
Anders Mälarstig	Pfizer, New York, NY, United States	anders.malarstig@pfizer.com	Steering Committee	Pharmaceutical companies
Katherine Klinger	Translational Sciences, Sanofi R&D, Framingham, MA, USA	katherine.klinger@sanofi.com	Steering Committee	Pharmaceutical companies
Deepak Raipal	Translational Sciences, Sanofi R&D, Framingham, MA, USA	deepak.rajpal@sanofi.com	Steering Committee	Pharmaceutical companies
Eric Green	Maze Therapeutics, San Francisco, CA, United States	egreen@mazetx.com	Steering Committee	Pharmaceutical companies
Robert Graham	Maze Therapeutics, San Francisco, CA, United States	rgraham@mazetx.com	Steering Committee	Pharmaceutical companies
Robert Yang	Janssen Biotech, Beerse, Belgium	ryang31@its.jnj.com	Steering Committee	Pharmaceutical companies
Chris O´Donnell	Novartis Institutes for BioMedical Research, Cambridge, MA, United States	chris.odonnell@novartis.com	Steering Committee	Pharmaceutical companies
Tomi P. Mäkelä	HiLIFE, University of Helsinki, Finland, Finland	tomi.makela@helsinki.fi	Steering Committee	University of Helsinki & Biobanks
Jaakko Kaprio	Institute for Molecular Medicine Finland (FIMM), HiLIFE, University of Helsinki, Helsinki, Finland	jaakko.kaprio@helsinki.fi	Steering Committee	University of Helsinki & Biobanks
Petri Virolainen	Auria Biobank / University of Turku / Hospital District of Southwest Finland, Turku, Finland	petri.virolainen@tyks.fi	Steering Committee	University of Helsinki & Biobanks
Antti Hakanen	Auria Biobank / University of Turku / Hospital District of Southwest Finland, Turku, Finland	antti.hakanen@tyks.fi	Steering Committee	University of Helsinki & Biobanks

Terhi Kilpi	THL Biobank / Finnish Institute for Health and Welfare (THL), Helsinki, Finland	terhi.kilpi@thl.fi	**Steering Committee**	**University of Helsinki & Biobanks**
Markus Perola	THL Biobank / Finnish Institute for Health and Welfare (THL), Helsinki, Finland	markus.perola@thl.fi	**Steering Committee**	**University of Helsinki & Biobanks**
Jukka Partanen	Finnish Red Cross Blood Service / Finnish Hematology Registry and Clinical Biobank, Helsinki, Finland	jukka.partanen@veripalvelu.fi	**Steering Committee**	**University of Helsinki & Biobanks**
Anne Pitkäranta	Helsinki Biobank / Helsinki University and Hospital District of Helsinki and Uusimaa, Helsinki	anne.pitkaranta@hus.fi	**Steering Committee**	**University of Helsinki & Biobanks**
Juhani Junttila	Northern Finland Biobank Borealis / University of Oulu / Northern Ostrobothnia Hospital District, Oulu, Finland	juhani.junttila@ppshp.fi	**Steering Committee**	**University of Helsinki & Biobanks**
Raisa Serpi	Northern Finland Biobank Borealis / University of Oulu / Northern Ostrobothnia Hospital District, Oulu, Finland	raisa.serpi@ppshp.fi	**Steering Committee**	**University of Helsinki & Biobanks**
Tarja Laitinen	Finnish Clinical Biobank Tampere / University of Tampere / Pirkanmaa Hospital District, Tampere, Finland	tarja.laitinen@pshp.fi	**Steering Committee**	**University of Helsinki & Biobanks**
Veli-Matti Kosma	Biobank of Eastern Finland / University of Eastern Finland / Northern Savo Hospital District, Kuopio, Finland	veli-matti.kosma@uef.fi	**Steering Committee**	**University of Helsinki & Biobanks**
Jari Laukkanen	Central Finland Biobank / University of Jyväskylä / Central Finland Health Care District, Jyväskylä, Finland	jari.laukkanen@ksshp.fi	**Steering Committee**	**University of Helsinki & Biobanks**
Marco Hautalahti	FINBB - Finnish biobank cooperative	marco.hautalahti@finbb.fi	**Steering Committee**	**University of Helsinki & Biobanks**
Outi Tuovila	Business Finland, Helsinki, Finland	outi.tuovila@businessfinland.fi	**Steering Committee**	**Other Experts/ Non-Voting Members**
Raimo Pakkanen	Business Finland, Helsinki, Finland	raimo.pakkanen@businessfinland.fi	**Steering Committee**	**Other Experts/ Non-Voting Members**
Jeffrey Waring	Abbvie, Chicago, IL, United States	jeff.waring@abbvie.com	**Scientific Committee**	**Pharmaceutical companies**
Bridget Riley-Gillis	Abbvie, Chicago, IL, United States	bridget.rileygillis@abbvie.com	**Scientific Committee**	**Pharmaceutical companies**
Fedik Rahimov	Abbvie, Chicago, IL, United States	fedik.rahimov@abbvie.com	**Scientific Committee**	**Pharmaceutical companies**
Ioanna Tachmazidou	Astra Zeneca, Cambridge, United Kingdom	ioanna.tachmazidou@astrazeneca.com	**Scientific Committee**	**Pharmaceutical companies**
Chia-Yen Chen	Biogen, Cambridge, MA, United States	chiayen.chen@biogen.com	**Scientific Committee**	**Pharmaceutical companies**
Heiko Runz	Biogen, Cambridge, MA, United States	heiko.runz@biogen.com	**Scientific Committee**	**Pharmaceutical companies**
Zhihao Ding	Boehringer Ingelheim, Ingelheim am Rhein, Germany	zhihao.ding@boehringer-ingelheim.com	**Scientific Committee**	**Pharmaceutical companies**
Marc Jung	Boehringer Ingelheim, Ingelheim am Rhein, Germany	marc_oliver.jung@boehringer-ingelheim.com	**Scientific Committee**	**Pharmaceutical companies**
Shameek Biswas	Bristol Myers Squibb, New York, NY, United States	Shameek.Biswas@bms.com	**Scientific Committee**	**Pharmaceutical companies**
Rion Pendergrass	Genentech, San Francisco, CA, United States	penders2@gene.com	**Scientific Committee**	**Pharmaceutical companies**
Julie Hunkapiller	Genentech, San Francisco, CA, United States	hunkapiller.julie@gene.com	**Scientific Committee**	**Pharmaceutical companies**
Margaret G. Ehm	GlaxoSmithKline, Collegeville, PA, United States	meg.g.ehm@gsk.com	**Scientific Committee**	**Pharmaceutical companies**
David Pulford	GlaxoSmithKline, Stevenage, United Kingdom	david.x.pulford@gsk.com	**Scientific Committee**	**Pharmaceutical companies**
Neha Raghavan	Merck, Kenilworth, NJ, United States	neha.raghavan@merck.com	**Scientific Committee**	**Pharmaceutical companies**
Adriana Huertas-Vazquez	Merck, Kenilworth, NJ, United States	adriana.huertas.vazquez@merck.com	**Scientific Committee**	**Pharmaceutical companies**
Jae-Hoon Sul	Merck, Kenilworth, NJ, United States	jae.hoon.sul@merck.com	**Scientific Committee**	**Pharmaceutical companies**
Anders Mälarstig	Pfizer, New York, NY, United States	anders.malarstig@pfizer.com	**Scientific Committee**	**Pharmaceutical companies**

Xinli Hu	Pfizer, New York, NY, United States	xinli.hu@pfizer.com	Scientific Committee	**Pharmaceutical companies**
Katherine Klinger	Translational Sciences, Sanofi R&D, Framingham, MA, USA	katherine.klinger@sanofi.com	Scientific Committee	**Pharmaceutical companies**
Robert Graham	Maze Therapeutics, San Francisco, CA, United States	rgraham@mazetx.com	Scientific Committee	**Pharmaceutical companies**
Eric Green	Maze Therapeutics, San Francisco, CA, United States	egreen@mazetx.com	Scientific Committee	**Pharmaceutical companies**
Sahar Mozaffari	Maze Therapeutics, San Francisco, CA, United States	smozaffari@mazetx.com	Scientific Committee	**Pharmaceutical companies**
Dawn Waterworth	Janssen Research & Development, LLC, Spring House, PA, United States	dwaterwo@its.jnj.com	Scientific Committee	**Pharmaceutical companies**
Nicole Renaud	Novartis Institutes for BioMedical Research, Cambridge, MA, United States	nicole.renaud@novartis.com	Scientific Committee	**Pharmaceutical companies**
Ma´en Obeidat	Novartis Institutes for BioMedical Research, Cambridge, MA, United States	maen.obeidat@novartis.com	Scientific Committee	**Pharmaceutical companies**
Samuli Ripatti	Institute for Molecular Medicine Finland (FIMM), HiLIFE, University of Helsinki, Helsinki, Finland	samuli.ripatti@helsinki.fi	Scientific Committee	**University of Helsinki & Biobanks**
Johanna Schleutker	Auria Biobank / Univ. of Turku / Hospital District of Southwest Finland, Turku, Finland	johanna.schleutker@utu.fi	Scientific Committee	**University of Helsinki & Biobanks**
Markus Perola	THL Biobank / Finnish Institute for Health and Welfare (THL), Helsinki, Finland	markus.perola@thl.fi	Scientific Committee	**University of Helsinki & Biobanks**
Mikko Arvas	Finnish Red Cross Blood Service / Finnish Hematology Registry and Clinical Biobank, Helsinki, Finland	mikko.arvas@veripalvelu.fi	Scientific Committee	**University of Helsinki & Biobanks**
Olli Carpén	Helsinki Biobank / Helsinki University and Hospital District of Helsinki and Uusimaa, Helsinki	olli.carpen@helsinki.fi	Scientific Committee	**University of Helsinki & Biobanks**
Reetta Hinttala	Northern Finland Biobank Borealis / University of Oulu / Northern Ostrobothnia Hospital District, Oulu, Finland	reetta.hinttala@oulu.fi	Scientific Committee	**University of Helsinki & Biobanks**
Johannes Kettunen	Northern Finland Biobank Borealis / University of Oulu / Northern Ostrobothnia Hospital District, Oulu, Finland	johannes.kettunen@oulu.fi	Scientific Committee	**University of Helsinki & Biobanks**
Arto Mannermaa	Biobank of Eastern Finland / University of Eastern Finland / Northern Savo Hospital District, Kuopio, Finland	arto.mannermaa@uef.fi	Scientific Committee	**University of Helsinki & Biobanks**
Katriina Aalto-Setälä	Faculty of Medicine and Health Technology, Tampere University, Tampere, Finland	katriina.aalto-setala@tuni.fi	Scientific Committee	**University of Helsinki & Biobanks**
Mika Kähönen	Finnish Clinical Biobank Tampere / University of Tampere / Pirkanmaa Hospital District, Tampere, Finland	mika.kahonen@uta.fi	Scientific Committee	**University of Helsinki & Biobanks**
Jari Laukkanen	Central Finland Biobank / University of Jyväskylä / Central Finland Health Care District, Jyväskylä, Finland	jari.laukkanen@ksshp.fi	Scientific Committee	**University of Helsinki & Biobanks**
Johanna Mäkelä	FINBB - Finnish biobank cooperative	johanna.makela@finbb.fi	Scientific Committee	**University of Helsinki & Biobanks**
Reetta Kälviäinen	Northern Savo Hospital District, Kuopio, Finland	reetta.kalviainen@kuh.fi	Clinical Groups	**Neurology Group**
Valtteri Julkunen	Northern Savo Hospital District, Kuopio, Finland	valtteri.julkunen@kuh.fi	Clinical Groups	**Neurology Group**
Hilkka Soininen	Northern Savo Hospital District, Kuopio, Finland	hilkka.soininen@uef.fi	Clinical Groups	**Neurology Group**
Anne Remes	Northern Ostrobothnia Hospital District, Oulu, Finland	anne.remes@oulu.fi	Clinical Groups	**Neurology Group**
Mikko Hiltunen	University of Eastern Finland, Kuopio, Finland	mikko.hiltunen@uef.fi	Clinical Groups	**Neurology Group**
Jukka Peltola	Pirkanmaa Hospital District, Tampere, Finland	jukka.peltola@pshp.fi	Clinical Groups	**Neurology Group**
Minna Raivio	Hospital District of Helsinki and Uusimaa, Helsinki, Finland	minna.raivio@geri.fi	Clinical Groups	**Neurology Group**
Pentti Tienari	Hospital District of Helsinki and Uusimaa, Helsinki, Finland	pentti.tienari@hus.fi	Clinical Groups	**Neurology Group**
Juha Rinne	Hospital District of Southwest Finland, Turku, Finland	juha.rinne@tyks.fi	Clinical Groups	**Neurology Group**

Roosa Kallionpää	Hospital District of Southwest Finland, Turku, Finland	roosa.kallionpaa@tyks.fi	**Clinical Groups**	**Neurology Group**
Juulia Partanen	Institute for Molecular Medicine Finland, HiLIFE, University of Helsinki, Finland	juulia.partanen@helsinki.fi	**Clinical Groups**	**Neurology Group**
Ali Abbasi	Abbvie, Chicago, IL, United States	ali.abbasi@abbvie.com	**Clinical Groups**	**Neurology Group**
Adam Ziemann	Abbvie, Chicago, IL, United States	adam.ziemann@abbvie.com	**Clinical Groups**	**Neurology Group**
Nizar Smaoui	Abbvie, Chicago, IL, United States	nizar.smaoui@abbvie.com	**Clinical Groups**	**Neurology Group**
Anne Lehtonen	Abbvie, Chicago, IL, United States	anne.lehtonen@abbvie.com	**Clinical Groups**	**Neurology Group**
Susan Eaton	Biogen, Cambridge, MA, United States	susan.eaton@biogen.com	**Clinical Groups**	**Neurology Group**
Heiko Runz	Biogen, Cambridge, MA, United States	heiko.runz@biogen.com	**Clinical Groups**	**Neurology Group**
Sanni Lahdenperä	Biogen, Cambridge, MA, United States	sanni.lahdenpera@biogen.com	**Clinical Groups**	**Neurology Group**
Shameek Biswas	Bristol Myers Squibb, New York, NY, United States	shameek.biswas@bms.com	**Clinical Groups**	**Neurology Group**
Julie Hunkapiller	Genentech, San Francisco, CA, United States	hunkapiller.julie@gene.com	**Clinical Groups**	**Neurology Group**
Natalie Bowers	Genentech, San Francisco, CA, United States	bowersn1@gene.com	**Clinical Groups**	**Neurology Group**
Edmond Teng	Genentech, San Francisco, CA, United States	teng.edmond@gene.com	**Clinical Groups**	**Neurology Group**
Rion Pendergrass	Genentech, San Francisco, CA, United States	penders2@gene.com	**Clinical Groups**	**Neurology Group**
Fanli Xu	GlaxoSmithKline, Brentford, United Kingdom	chun-fang.2.xu@gsk.com	**Clinical Groups**	**Neurology Group**
David Pulford	GlaxoSmithKline, Stevenage, United Kingdom	david.x.pulford@gsk.com	**Clinical Groups**	**Neurology Group**
Kirsi Auro	GlaxoSmithKline, Espoo, Finland	kirsi.m.auro@gsk.com	**Clinical Groups**	**Neurology Group**
Laura Addis	GlaxoSmithKline, Brentford, United Kingdom	laura.x.addis@gsk.com	**Clinical Groups**	**Neurology Group**
John Eicher	GlaxoSmithKline, Brentford, United Kingdom	john.d.eicher@gsk.com	**Clinical Groups**	**Neurology Group**
Qingqin S Li	Janssen Research & Development, LLC, Titusville, NJ 08560, United States	QLi2@its.jnj.com	**Clinical Groups**	**Neurology Group**
Karen He	Janssen Research & Development, LLC, Spring House, PA, United States	khe2@its.jnj.com	**Clinical Groups**	**Neurology Group**
Ekaterina Khramtsova	Janssen Research & Development, LLC, Spring House, PA, United States	ekhramts@its.jnj.com	**Clinical Groups**	**Neurology Group**
Neha Raghavan	Merck, Kenilworth, NJ, United States	neha.raghavan@merck.com	**Clinical Groups**	**Neurology Group**
Martti Färkkilä	Hospital District of Helsinki and Uusimaa, Helsinki, Finland	martti.farkkila@hus.fi	**Clinical Groups**	**Gastroenterology Group**
Jukka Koskela	Hospital District of Helsinki and Uusimaa, Helsinki, Finland	jukka.koskela@helsinki.fi	**Clinical Groups**	**Gastroenterology Group**
Sampsa Pikkarainen	Hospital District of Helsinki and Uusimaa, Helsinki, Finland	sampsa.pikkarainen@hus.fi	**Clinical Groups**	**Gastroenterology Group**
Airi Jussila	Pirkanmaa Hospital District, Tampere, Finland	airi.jussila@pshp.fi	**Clinical Groups**	**Gastroenterology Group**
Katri Kaukinen	Pirkanmaa Hospital District, Tampere, Finland	katri.kaukinen@tuni.fi	**Clinical Groups**	**Gastroenterology Group**
Timo Blomster	Northern Ostrobothnia Hospital District, Oulu, Finland	timo.blomster@ppshp.fi	**Clinical Groups**	**Gastroenterology Group**
Mikko Kiviniemi	Northern Savo Hospital District, Kuopio, Finland	mikko.kiviniemi@kuh.fi	**Clinical Groups**	**Gastroenterology Group**
Markku Voutilainen	Hospital District of Southwest Finland, Turku, Finland	markku.voutilainen@tyks.fi	**Clinical Groups**	**Gastroenterology Group**
Mark Daly	Institute for Molecular Medicine, Finland (FIMM), HiLIFE, University of Helsinki, Helsinki, Finland; Broad Institute of MIT and Harvard; Massachusetts General Hospital	mark.daly@helsinki.fi	**Clinical Groups**	**Gastroenterology Group**
Ali Abbasi	Abbvie, Chicago, IL, United States	ali.abbasi@abbvie.com	**Clinical Groups**	**Gastroenterology Group**
Jeffrey Waring	Abbvie, Chicago, IL, United States	jeff.waring@abbvie.com	**Clinical Groups**	**Gastroenterology Group**

Nizar Smaoui	Abbvie, Chicago, IL, United States	nizar.smaoui@abbvie.com	Clinical Groups	**Gastroenterology Group**
Fedik Rahimov	Abbvie, Chicago, IL, United States	fedik.rahimov@abbvie.com	Clinical Groups	**Gastroenterology Group**
Anne Lehtonen	Abbvie, Chicago, IL, United States	anne.lehtonen@abbvie.com	Clinical Groups	**Gastroenterology Group**
Tim Lu	Genentech, San Francisco, CA, United States	lut8@gene.com	Clinical Groups	**Gastroenterology Group**
Natalie Bowers	Genentech, San Francisco, CA, United States	bowersn1@gene.com	Clinical Groups	**Gastroenterology Group**
Rion Pendergrass	Genentech, San Francisco, CA, United States	penders2@gene.com	Clinical Groups	**Gastroenterology Group**
Linda McCarthy	GlaxoSmithKline, Brentford, United Kingdom	linda.c.mccarthy@gsk.com	Clinical Groups	**Gastroenterology Group**
Amy Hart	Janssen Research & Development, LLC, Spring House, PA, United States	ahart13@its.jnj.com	Clinical Groups	**Gastroenterology Group**
Meijian Guan	Janssen Research & Development, LLC, Spring House, PA, United States	mguan4@its.jnj.com	Clinical Groups	**Gastroenterology Group**
Jason Miller	Merck, Kenilworth, NJ, United States	jason.miller4@merck.com	Clinical Groups	**Gastroenterology Group**
Kirsi Kalpala	Pfizer, New York, NY, United States	kirsi.kalpala@pfizer.com	Clinical Groups	**Gastroenterology Group**
Melissa Miller	Pfizer, New York, NY, United States	melissa.r.miller@pfizer.com	Clinical Groups	**Gastroenterology Group**
Xinli Hu	Pfizer, New York, NY, United States	xinli.hu@pfizer.com	Clinical Groups	**Gastroenterology Group**
Kari Eklund	Hospital District of Helsinki and Uusimaa, Helsinki, Finland	kari.eklund@hus.fi	Clinical Groups	**Rheumatology Group**
Antti Palomäki	Hospital District of Southwest Finland, Turku, Finland	ajpalo@utu.fi	Clinical Groups	**Rheumatology Group**
Pia Isomäki	Pirkanmaa Hospital District, Tampere, Finland	pia.isomaki@pshp.fi	Clinical Groups	**Rheumatology Group**
Laura Pirilä	Hospital District of Southwest Finland, Turku, Finland	laura.pirila@fimnet.fi,laura.pirila@tyks.fi	Clinical Groups	**Rheumatology Group**
Oili Kaipiainen-Seppänen	Northern Savo Hospital District, Kuopio, Finland	oili.kaipiainen-seppanen@kuh.fi	Clinical Groups	**Rheumatology Group**
Johanna Huhtakangas	Northern Ostrobothnia Hospital District, Oulu, Finland	johanna.huhtakangas@kuh.fi	Clinical Groups	**Rheumatology Group**
Nina Mars	Institute for Molecular Medicine Finland (FIMM), HiLIFE, University of Helsinki, Helsinki, Finland	nina.mars@helsinki.fi	Clinical Groups	**Rheumatology Group**
Ali Abbasi	Abbvie, Chicago, IL, United States	ali.abbasi@abbvie.com	Clinical Groups	**Rheumatology Group**
Jeffrey Waring	Abbvie, Chicago, IL, United States	jeff.waring@abbvie.com	Clinical Groups	**Rheumatology Group**
Fedik Rahimov	Abbvie, Chicago, IL, United States	fedik.rahimov@abbvie.com	Clinical Groups	**Rheumatology Group**
Apinya Lertratanakul	Abbvie, Chicago, IL, United States	apinya.lertratanakul@abbvie.com	Clinical Groups	**Rheumatology Group**
Nizar Smaoui	Abbvie, Chicago, IL, United States	nizar.smaoui@abbvie.com	Clinical Groups	**Rheumatology Group**
Anne Lehtonen	Abbvie, Chicago, IL, United States	anne.lehtonen@abbvie.com	Clinical Groups	**Rheumatology Group**
David Close	Astra Zeneca, Cambridge, United Kingdom	david.close@astrazeneca.com	Clinical Groups	**Rheumatology Group**
Marla Hochfeld	Bristol Myers Squibb, New York, NY, United States	mhochfeld@celgene.com	Clinical Groups	**Rheumatology Group**
Natalie Bowers	Genentech, San Francisco, CA, United States	bowersn1@gene.com	Clinical Groups	**Rheumatology Group**
Rion Pendergrass	Genentech, San Francisco, CA, United States	penders2@gene.com	Clinical Groups	**Rheumatology Group**
Jorge Esparza Gordillo	GlaxoSmithKline, Brentford, United Kingdom	jorge.x.esparza-gordillo@gsk.com	Clinical Groups	**Rheumatology Group**
Kirsi Auro	GlaxoSmithKline, Espoo, Finland	kirsi.m.auro@gsk.com	Clinical Groups	**Rheumatology Group**
Dawn Waterworth	Janssen Research & Development, LLC, Spring House, PA, United States	dwaterwo@its.jnj.com	Clinical Groups	**Rheumatology Group**
Fabiana Farias	Merck, Kenilworth, NJ, United States	fabiana.farias@merck.com	Clinical Groups	**Rheumatology Group**
Kirsi Kalpala	Pfizer, New York, NY, United States	kirsi.kalpala@pfizer.com	Clinical Groups	**Rheumatology Group**

Nan Bing	Pfizer, New York, NY, United States	nan.bing@pfizer.com	**Clinical Groups**	**Rheumatology Group**
Xinli Hu	Pfizer, New York, NY, United States	xinli.hu@pfizer.com	**Clinical Groups**	**Rheumatology Group**
Tarja Laitinen	Pirkanmaa Hospital District, Tampere, Finland	tarja.laitinen@pshp.fi	**Clinical Groups**	**Pulmonology Group**
Margit Pelkonen	Northern Savo Hospital District, Kuopio, Finland	margit.pelkonen@kuh.fi	**Clinical Groups**	**Pulmonology Group**
Paula Kauppi	Hospital District of Helsinki and Uusimaa, Helsinki, Finland	paula.kauppi@hus.fi	**Clinical Groups**	**Pulmonology Group**
Hannu Kankaanranta	University of Gothenburg, Gothenburg, Sweden/ Seinäjoki Central Hospital, Seinäjoki, Finland/ Tampere University, Tampere, Finland	hannu.kankaanranta@tuni.fi	**Clinical Groups**	**Pulmonology Group**
Terttu Harju	Northern Ostrobothnia Hospital District, Oulu, Finland	terttu.harju@oulu.fi	**Clinical Groups**	**Pulmonology Group**
Riitta Lahesmaa	Hospital District of Southwest Finland, Turku, Finland	rilahes@utu.fi	**Clinical Groups**	**Pulmonology Group**
Nizar Smaoui	Abbvie, Chicago, IL, United States	nizar.smaoui@abbvie.com	**Clinical Groups**	**Pulmonology Group**
Alex Mackay	Astra Zeneca, Cambridge, United Kingdom	alex.mackay@astrazeneca.com	**Clinical Groups**	**Pulmonology Group**
Glenda Lassi	Astra Zeneca, Cambridge, United Kingdom	glenda.lassi@astrazeneca.com	**Clinical Groups**	**Pulmonology Group**
Susan Eaton	Biogen, Cambridge, MA, United States	susan.eaton@biogen.com	**Clinical Groups**	**Pulmonology Group**
Hubert Chen	Genentech, San Francisco, CA, United States	chenh37@gene.com	**Clinical Groups**	**Pulmonology Group**
Rion Pendergrass	Genentech, San Francisco, CA, United States	penders2@gene.com	**Clinical Groups**	**Pulmonology Group**
Natalie Bowers	Genentech, San Francisco, CA, United States	bowersn1@gene.com	**Clinical Groups**	**Pulmonology Group**
Joanna Betts	GlaxoSmithKline, Brentford, United Kingdom	joanna.c.betts@gsk.com	**Clinical Groups**	**Pulmonology Group**
Kirsi Auro	GlaxoSmithKline, Espoo, Finland	kirsi.m.auro@gsk.com	**Clinical Groups**	**Pulmonology Group**
Rajashree Mishra	GlaxoSmithKline, Brentford, United Kingdom	rajashree.x.mishra@gsk.com	**Clinical Groups**	**Pulmonology Group**
Majd Mouded	Novartis, Basel, Switzerland	majd.mouded@novartis.com	**Clinical Groups**	**Pulmonology Group**
Debby Ngo	Novartis, Basel, Switzerland	debby.ngo@novartis.com	**Clinical Groups**	**Pulmonology Group**
Teemu Niiranen	Finnish Institute for Health and Welfare (THL), Helsinki, Finland	teemu.niiranen@thl.fi	**Clinical Groups**	**Cardiometabolic Diseases Group**
Felix Vaura	Finnish Institute for Health and Welfare (THL), Helsinki, Finland	fechva@utu.fi	**Clinical Groups**	**Cardiometabolic Diseases Group**
Veikko Salomaa	Finnish Institute for Health and Welfare (THL), Helsinki, Finland	veikko.salomaa@thl.fi	**Clinical Groups**	**Cardiometabolic Diseases Group**
Kaj Metsärinne	Hospital District of Southwest Finland, Turku, Finland	kaj.metsarinne@tyks.fi	**Clinical Groups**	**Cardiometabolic Diseases Group**
Jenni Aittokallio	Hospital District of Southwest Finland, Turku, Finland	jemato@utu.fi	**Clinical Groups**	**Cardiometabolic Diseases Group**
Mika Kähönen	Pirkanmaa Hospital District, Tampere, Finland	mika.kahonen@uta.fi	**Clinical Groups**	**Cardiometabolic Diseases Group**
Jussi Hernesniemi	Pirkanmaa Hospital District, Tampere, Finland	jussi.hernesniemi@tuni.fi	**Clinical Groups**	**Cardiometabolic Diseases Group**
Daniel Gordin	Hospital District of Helsinki and Uusimaa, Helsinki, Finland	daniel.gordin@hus.fi	**Clinical Groups**	**Cardiometabolic Diseases Group**
Juha Sinisalo	Hospital District of Helsinki and Uusimaa, Helsinki, Finland	juha.sinisalo@hus.fi	**Clinical Groups**	**Cardiometabolic Diseases Group**
Marja-Riitta Taskinen	Hospital District of Helsinki and Uusimaa, Helsinki, Finland	marja-riitta.taskinen@helsinki.fi	**Clinical Groups**	**Cardiometabolic Diseases Group**
Tiinamaija Tuomi	Hospital District of Helsinki and Uusimaa, Helsinki, Finland	tiinamaija.tuomi@hus.fi	**Clinical Groups**	**Cardiometabolic Diseases Group**
Timo Hiltunen	Hospital District of Helsinki and Uusimaa, Helsinki, Finland	timo.hiltunen@hus.fi	**Clinical Groups**	**Cardiometabolic Diseases Group**
Jari Laukkanen	Central Finland Health Care District, Jyväskylä, Finland	jari.laukkanen@ksshp.fi	**Clinical Groups**	**Cardiometabolic Diseases Group**

Amanda Elliott	Institute for Molecular Medicine Finland (FIMM), HiLIFE, University of Helsinki, Helsinki, Finland; Broad Institute, Cambridge, MA, USA and Massachusetts General Hospital, Boston,	aelliott@broadinstitute.org	Clinical Groups	**Cardiometabolic Diseases Group**
Mary Pat Reeve	Institute for Molecular Medicine Finland (FIMM), HiLIFE, University of Helsinki, Helsinki, Finland	mary.reeve@helsinki.fi	Clinical Groups	**Cardiometabolic Diseases Group**
Sanni Ruotsalainen	Institute for Molecular Medicine Finland (FIMM), HiLIFE, University of Helsinki, Helsinki, Finland	sanni.ruotsalainen@helsinki.fi	Clinical Groups	**Cardiometabolic Diseases Group**
Benjamin Challis	Astra Zeneca, Cambridge, United Kingdom	benjamin.challis@astrazeneca.com	Clinical Groups	**Cardiometabolic Diseases Group**
Dirk Paul	Astra Zeneca, Cambridge, United Kingdom	dirk.paul@astrazeneca.com	Clinical Groups	**Cardiometabolic Diseases Group**
Julie Hunkapiller	Genentech, San Francisco, CA, United States	hunkapiller.julie@gene.com	Clinical Groups	**Cardiometabolic Diseases Group**
Natalie Bowers	Genentech, San Francisco, CA, United States	bowersn1@gene.com	Clinical Groups	**Cardiometabolic Diseases Group**
Rion Pendergrass	Genentech, San Francisco, CA, United States	penders2@gene.com	Clinical Groups	**Cardiometabolic Diseases Group**
Audrey Chu	GlaxoSmithKline, Brentford, United Kingdom	audrey.y.chu@gsk.com	Clinical Groups	**Cardiometabolic Diseases Group**
Kirsi Auro	GlaxoSmithKline, Espoo, Finland	kirsi.m.auro@gsk.com	Clinical Groups	**Cardiometabolic Diseases Group**
Dermot Reilly	Janssen Research & Development, LLC, Boston, MA, United States	dreill11@its.jnj.com	Clinical Groups	**Cardiometabolic Diseases Group**
Mike Mendelson	Novartis, Boston, MA, United States	mike.mendelson@novartis.com	Clinical Groups	**Cardiometabolic Diseases Group**
Jaakko Parkkinen	Pfizer, New York, NY, United States	jaakko.parkkinen@pfizer.com	Clinical Groups	**Cardiometabolic Diseases Group**
Melissa Miller	Pfizer, New York, NY, United States	melissa.r.miller@pfizer.com	Clinical Groups	**Cardiometabolic Diseases Group**
Tuomo Meretoja	Hospital District of Helsinki and Uusimaa, Helsinki, Finland	tuomo.meretoja@hus.fi	Clinical Groups	**Oncology Group**
Heikki Joensuu	Hospital District of Helsinki and Uusimaa, Helsinki, Finland	heikki.joensuu@hus.fi	Clinical Groups	**Oncology Group**
Olli Carpén	Hospital District of Helsinki and Uusimaa, Helsinki, Finland	olli.carpen@helsinki.fi	Clinical Groups	**Oncology Group**
Johanna Mattson	Hospital District of Helsinki and Uusimaa, Helsinki, Finland	johanna.mattson@hus.fi	Clinical Groups	**Oncology Group**
Eveliina Salminen	Hospital District of Helsinki and Uusimaa, Helsinki, Finland	eveliina.e.salminen@hus.fi	Clinical Groups	**Oncology Group**
Annika Auranen	Pirkanmaa Hospital District , Tampere, Finland	anaura@utu.fi	Clinical Groups	**Oncology Group**
Peeter Karihtala	Northern Ostrobothnia Hospital District, Oulu, Finland	peeter.karihtala@oulu.fi	Clinical Groups	**Oncology Group**
Päivi Auvinen	Northern Savo Hospital District, Kuopio, Finland	paivi.auvinen@kuh.fi	Clinical Groups	**Oncology Group**
Klaus Elenius	Hospital District of Southwest Finland, Turku, Finland	klaus.elenius@utu.fi	Clinical Groups	**Oncology Group**
Johanna Schleutker	Hospital District of Southwest Finland, Turku, Finland	johanna.schleutker@utu.fi	Clinical Groups	**Oncology Group**
Esa Pitkänen	Institute for Molecular Medicine Finland (FIMM), HiLIFE, University of Helsinki, Helsinki, Finland	esa.pitkanen@helsinki.fi	Clinical Groups	**Oncology Group**
Nina Mars	Institute for Molecular Medicine Finland (FIMM), HiLIFE, University of Helsinki, Helsinki, Finland	nina.mars@helsinki.fi	Clinical Groups	**Oncology Group**
Mark Daly	Institute for Molecular Medicine Finland (FIMM), HiLIFE, University of Helsinki, Helsinki, Finland; Broad Institute of MIT and Harvard; Massachusetts General Hospital	mark.daly@helsinki.fi	Clinical Groups	**Oncology Group**
Relja Popovic	Abbvie, Chicago, IL, United States	relja.popovic@abbvie.com	Clinical Groups	**Oncology Group**
Jeffrey Waring	Abbvie, Chicago, IL, United States	jeff.waring@abbvie.com	Clinical Groups	**Oncology Group**
Bridget Riley-Gillis	Abbvie, Chicago, IL, United States	bridget.rileygillis@abbvie.com	Clinical Groups	**Oncology Group**
Anne Lehtonen	Abbvie, Chicago, IL, United States	anne.lehtonen@abbvie.com	Clinical Groups	**Oncology Group**
Jennifer Schutzman	Genentech, San Francisco, CA, United States	schutzman.jennifer@gene.com	Clinical Groups	**Oncology Group**
Julie Hunkapiller	Genentech, San Francisco, CA, United States	hunkapiller.julie@gene.com	Clinical Groups	**Oncology Group**

Natalie Bowers	Genentech, San Francisco, CA, United States	bowersn1@gene.com	Clinical Groups	**Oncology Group**
Rion Pendergrass	Genentech, San Francisco, CA, United States	penders2@gene.com	Clinical Groups	**Oncology Group**
Diptee Kulkarni	GlaxoSmithKline, Brentford, United Kingdom	diptee.a.kulkarni@gsk.com	Clinical Groups	**Oncology Group**
Kirsi Auro	GlaxoSmithKline, Espoo, Finland	kirsi.m.auro@gsk.com	Clinical Groups	**Oncology Group**
Alessandro Porello	Janssen Research & Development, LLC, Spring House, PA, United States	APorrell@ITS.JNJ.com	Clinical Groups	**Oncology Group**
Andrey Loboda	Merck, Kenilworth, NJ, United States	andrey_loboda@merck.com	Clinical Groups	**Oncology Group**
Heli Lehtonen	Pfizer, New York, NY, United States	heli.lehtonen@pfizer.com	Clinical Groups	**Oncology Group**
Stefan McDonough	Pfizer, New York, NY, United States	stefan.McDonough@pfizer.com	Clinical Groups	**Oncology Group**
Sauli Vuoti	Janssen-Cilag Oy, Espoo, Finland	svuoti@its.jnj.com	Clinical Groups	**Oncology Group**
Kai Kaarniranta	Northern Savo Hospital District, Kuopio, Finland	kai.kaarniranta@uef.fi	Clinical Groups	**Opthalmology Group**
Joni A Turunen	Helsinki University Hospital and University of Helsinki, Helsinki, Finland; Eye Genetics Group, Folkhälsan Research Center, Helsinki, Finland	joni.turunen@helsinki.fi	Clinical Groups	**Opthalmology Group**
Terhi Ollila	Hospital District of Helsinki and Uusimaa, Helsinki, Finland	terhi.ollila@hus.fi	Clinical Groups	**Opthalmology Group**
Hannu Uusitalo	Pirkanmaa Hospital District, Tampere, Finland	hannu.uusitalo@tuni.fi	Clinical Groups	**Opthalmology Group**
Juha Karjalainen	Institute for Molecular Medicine Finland (FIMM), HiLIFE, University of Helsinki, Helsinki, Finland	juha.karjalainen@helsinki.fi	Clinical Groups	**Opthalmology Group**
Esa Pitkänen	Institute for Molecular Medicine Finland (FIMM), HiLIFE, University of Helsinki, Helsinki, Finland	esa.pitkanen@helsinki.fi	Clinical Groups	**Opthalmology Group**
Mengzhen Liu	Abbvie, Chicago, IL, United States	mengzhen.liu@abbvie.com	Clinical Groups	**Opthalmology Group**
Heiko Runz	Biogen, Cambridge, MA, United States	heiko.runz@biogen.com	Clinical Groups	**Opthalmology Group**
Stephanie Loomis	Biogen, Cambridge, MA, United States	stephanie.loomis@biogen.com	Clinical Groups	**Opthalmology Group**
Erich Strauss	Genentech, San Francisco, CA, United States	strauss.erich@gene.com	Clinical Groups	**Opthalmology Group**
Natalie Bowers	Genentech, San Francisco, CA, United States	bowersn1@gene.com	Clinical Groups	**Opthalmology Group**
Hao Chen	Genentech, San Francisco, CA, United States	haoc@gene.com	Clinical Groups	**Opthalmology Group**
Rion Pendergrass	Genentech, San Francisco, CA, United States	penders2@gene.com	Clinical Groups	**Opthalmology Group**
Kaisa Tasanen	Northern Ostrobothnia Hospital District, Oulu, Finland	kaisa.tasanen-maatta@oulu.fi	Clinical Groups	**Dermatology Group**
Laura Huilaja	Northern Ostrobothnia Hospital District, Oulu, Finland	laura.huilaja@oulu.fi	Clinical Groups	**Dermatology Group**
Katariina Hannula-Jouppi	Hospital District of Helsinki and Uusimaa, Helsinki, Finland	katariina.hannula-jouppi@hus.fi	Clinical Groups	**Dermatology Group**
Teea Salmi	Pirkanmaa Hospital District, Tampere, Finland	teea.salmi@pshp.fi	Clinical Groups	**Dermatology Group**
Sirkku Peltonen	Hospital District of Southwest Finland, Turku, Finland	sipelto@utu.fi	Clinical Groups	**Dermatology Group**
Leena Koulu	Hospital District of Southwest Finland, Turku, Finland	leena.koulu@tyks.fi	Clinical Groups	**Dermatology Group**
Nizar Smaoui	Abbvie, Chicago, IL, United States	nizar.smaoui@abbvie.com	Clinical Groups	**Dermatology Group**
Fedik Rahimov	Abbvie, Chicago, IL, United States	fedik.rahimov@abbvie.com	Clinical Groups	**Dermatology Group**
Anne Lehtonen	Abbvie, Chicago, IL, United States	anne.lehtonen@abbvie.com	Clinical Groups	**Dermatology Group**
David Choy	Genentech, San Francisco, CA, United States	choy.david@gene.com	Clinical Groups	**Dermatology Group**
Rion Pendergrass	Genentech, San Francisco, CA, United States	penders2@gene.com	Clinical Groups	**Dermatology Group**
Dawn Waterworth	Janssen Research & Development, LLC, Spring House, PA, United States	dwaterwo@its.jnj.com	Clinical Groups	**Dermatology Group**

Kirsi Kalpala	Pfizer, New York, NY, United States	kirsi.kalpala@pfizer.com	**Clinical Groups**	**Dermatology Group**
Ying Wu	Pfizer, New York, NY, United States	ying.wu3@pfizer.com	**Clinical Groups**	**Dermatology Group**
Pirkko Pussinen	Hospital District of Helsinki and Uusimaa, Helsinki, Finland	pirkko.pussinen@helsinki.fi	**Clinical Groups**	**Odontology Group**
Aino Salminen	Hospital District of Helsinki and Uusimaa, Helsinki, Finland	aino.m.salminen@helsinki.fi	**Clinical Groups**	**Odontology Group**
Tuula Salo	Hospital District of Helsinki and Uusimaa, Helsinki, Finland	tuula.salo@helsinki.fi	**Clinical Groups**	**Odontology Group**
David Rice	Hospital District of Helsinki and Uusimaa, Helsinki, Finland	david.rice@helsinki.fi	**Clinical Groups**	**Odontology Group**
Pekka Nieminen	Hospital District of Helsinki and Uusimaa, Helsinki, Finland	pekka.nieminen@helsinki.fi	**Clinical Groups**	**Odontology Group**
Ulla Palotie	Hospital District of Helsinki and Uusimaa, Helsinki, Finland	ulla.palotie@helsinki.fi	**Clinical Groups**	**Odontology Group**
Maria Siponen	Northern Savo Hospital District, Kuopio, Finland	maria.siponen@uef.fi	**Clinical Groups**	**Odontology Group**
Liisa Suominen	Northern Savo Hospital District, Kuopio, Finland	liisa.suominen@uef.fi	**Clinical Groups**	**Odontology Group**
Päivi Mäntylä	Northern Savo Hospital District, Kuopio, Finland	paivi.mantyla@uef.fi	**Clinical Groups**	**Odontology Group**
Ulvi Gursoy	Hospital District of Southwest Finland, Turku, Finland	ulvi.gursoy@utu.fi	**Clinical Groups**	**Odontology Group**
Vuokko Anttonen	Northern Ostrobothnia Hospital District, Oulu, Finland	vuokko.anttonen@oulu.fi	**Clinical Groups**	**Odontology Group**
Kirsi Sipilä	Research Unit of Oral Health Sciences Faculty of Medicine, University of Oulu, Oulu, Finland; Medical Research Center, Oulu, Oulu University Hospital and University of Oulu, Oulu, Finland	kirsi.sipila@oulu.fi	**Clinical Groups**	**Odontology Group**
Rion Pendergrass	Genentech, San Francisco, CA, United States	pendergrass.sarah@gene.com	**Clinical Groups**	**Odontology Group**
Hannele Laivuori	Institute for Molecular Medicine Finland (FIMM), HiLIFE, University of Helsinki, Helsinki, Finland	hannele.laivuori@helsinki.fi	**Clinical Groups**	**Women's Health and Reproduction Group**
Venla Kurra	Pirkanmaa Hospital District, Tampere, Finland	venla.kurra@tuni.fi	**Clinical Groups**	**Women's Health and Reproduction Group**
Laura Kotaniemi-Talonen	Pirkanmaa Hospital District, Tampere, Finland	laura.kotaniemi-talonen@tuni.fi	**Clinical Groups**	**Women's Health and Reproduction Group**
Oskari Heikinheimo	Hospital District of Helsinki and Uusimaa, Helsinki, Finland	oskari.heikinheimo@helsinki.fi	**Clinical Groups**	**Women's Health and Reproduction Group**
Ilkka Kalliala	Hospital District of Helsinki and Uusimaa, Helsinki, Finland	ilkka.kalliala@hus.fi	**Clinical Groups**	**Women's Health and Reproduction Group**
Lauri Aaltonen	Hospital District of Helsinki and Uusimaa, Helsinki, Finland	lauri.aaltonen@helsinki.fi	**Clinical Groups**	**Women's Health and Reproduction Group**
Varpu Jokimaa	Hospital District of Southwest Finland, Turku, Finland	varpu.jokimaa@utu.fi	**Clinical Groups**	**Women's Health and Reproduction Group**
Johannes Kettunen	Northern Ostrobothnia Hospital District, Oulu, Finland	Johannes.Kettunen@oulu.fi	**Clinical Groups**	**Women's Health and Reproduction Group**
Marja Vääräsmäki	Northern Ostrobothnia Hospital District, Oulu, Finland	marja.vaarasmaki@oulu.fi	**Clinical Groups**	**Women's Health and Reproduction Group**
Outi Uimari	Northern Ostrobothnia Hospital District, Oulu, Finland	outi.uimari@oulu.fi	**Clinical Groups**	**Women's Health and Reproduction Group**
Laure Morin-Papunen	Northern Ostrobothnia Hospital District, Oulu, Finland	lmp@cc.oulu.fi	**Clinical Groups**	**Women's Health and Reproduction Group**
Maarit Niinimäki	Northern Ostrobothnia Hospital District, Oulu, Finland	maarit.niinimaki@oulu.fi	**Clinical Groups**	**Women's Health and Reproduction Group**
Terhi Piltonen	Northern Ostrobothnia Hospital District, Oulu, Finland	terhi.piltonen@oulu.fi	**Clinical Groups**	**Women's Health and Reproduction Group**
Katja Kivinen	Institute for Molecular Medicine Finland (FIMM), HiLIFE, University of Helsinki, Helsinki, Finland	katja.kivinen@helsinki.fi	**Clinical Groups**	**Women's Health and Reproduction Group**
Elisabeth Widen	Institute for Molecular Medicine Finland (FIMM), HiLIFE, University of Helsinki, Helsinki, Finland	elisabeth.widen@helsinki.fi	**Clinical Groups**	**Women's Health and Reproduction Group**
Taru Tukiainen	Institute for Molecular Medicine Finland (FIMM), HiLIFE, University of Helsinki, Helsinki, Finland	taru.tukiainen@helsinki.fi	**Clinical Groups**	**Women's Health and Reproduction Group**
Mary Pat Reeve	Institute for Molecular Medicine Finland (FIMM), HiLIFE, University of Helsinki, Helsinki, Finland	mary.reeve@helsinki.fi	**Clinical Groups**	**Women's Health and Reproduction Group**
Mark Daly	Institute for Molecular Medicine Finland (FIMM), HiLIFE, University of Helsinki, Helsinki, Finland; Broad Institute of MIT and Harvard; Massachusetts General Hospital	mark.daly@helsinki.fi	**Clinical Groups**	**Women's Health and Reproduction Group**

Niko Välimäki	University of Helsinki, Helsinki, Finland	niko.valimaki@helsinki.fi	Clinical Groups	**Women's Health and Reproduction Group**
Eija Laakkonen	University of Jyväskylä, Jyväskylä, Finland	eija.k.laakkonen@jyu.fi	Clinical Groups	**Women's Health and Reproduction Group**
Jaakko Tyrmi	University of Oulu, Oulu, Finland / University of Tampere, Tampere, Finland	jaakko.tyrmi@oulu.fi	Clinical Groups	**Women's Health and Reproduction Group**
Heidi Silven	University of Oulu, Oulu, Finland	heidi.silven@student.oulu.fi	Clinical Groups	**Women's Health and Reproduction Group**
Eeva Sliz	University of Oulu, Oulu, Finland	eeva.sliz@oulu.fi	Clinical Groups	**Women's Health and Reproduction Group**
Riikka Arffman	University of Oulu, Oulu, Finland	riikka.arffman@oulu.fi	Clinical Groups	**Women's Health and Reproduction Group**
Susanna Savukoski	University of Oulu, Oulu, Finland	susanna.savukoski@oulu.fi	Clinical Groups	**Women's Health and Reproduction Group**
Triin Laisk	Estonian biobank, Tartu, Estonia	triin.laisk@ut.ee	Clinical Groups	**Women's Health and Reproduction Group**
Natalia Pujol	Estonian biobank, Tartu, Estonia	natalia.pujolgualdo@oulu.fi	Clinical Groups	**Women's Health and Reproduction Group**
Mengzhen Liu	Abbvie, Chicago, IL, United States	mengzhen.liu@abbvie.com	Clinical Groups	**Women's Health and Reproduction Group**
Bridget Riley-Gillis	Abbvie, Chicago, IL, United States	bridget.rileygillis@abbvie.com	Clinical Groups	**Women's Health and Reproduction Group**
Rion Pendergrass	Genentech, San Francisco, CA, United States	penders2@gene.com	Clinical Groups	**Women's Health and Reproduction Group**
Janet Kumar	GlaxoSmithKline, Collegeville, PA, United States	janet.x.kumar@gsk.com	Clinical Groups	**Women's Health and Reproduction Group**
Kirsi Auro	GlaxoSmithKline, Espoo, Finland	kirsi.m.auro@gsk.com	Clinical Groups	**Women's Health and Reproduction Group**
Iiris Hovatta	University of Helsinki, Finland	iiris.hovatta@helsinki.fi	Clinical Groups	**Depression group**
Chia-Yen Chen	Biogen, Cambridge, MA, United States	chiayen.chen@biogen.com	Clinical Groups	**Depression group**
Erkki Isometsä	Hospital District of Helsinki and Uusimaa, Helsinki, Finland	erkki.isometsa@hus.fi	Clinical Groups	**Depression group**
Kumar Veerapen	Broad Institute, Cambridge, MA, United States	veerapen@broadinstitute.org	Clinical Groups	**Depression group**
Hanna Ollila	Institute for Molecular Medicine Finland (FIMM), HiLIFE, University of Helsinki, Helsinki, Finland	hanna.m.ollila@helsinki.fi	Clinical Groups	**Depression group**
Jaana Suvisaari	Finnish Institute for Health and Welfare (THL), Helsinki, Finland	jaana.suvisaari@thl.fi	Clinical Groups	**Depression group**
Thomas Damm Als	Aarhus University, Denmark	tda@biomed.au.dk	Clinical Groups	**Depression group**
Antti Mäkitie	Department of Otorhinolaryngology - Head and Neck Surgery, University of Helsinki and Helsinki University Hospital, Helsinki, Finland	antti.makitie@helsinki.fi	Clinical Groups	**ENT (ear, nose and throath) Group**
Argyro Bizaki-Vallaskangas	Pirkanmaa Hospital District, Tampere, Finland	argyro.bizaki-vallaskangas@tuni.fi	Clinical Groups	**ENT (ear, nose and throath) Group**
Sanna Toppila-Salmi	University of Helsinki, Finland	sanna.salmi@helsinki.fi	Clinical Groups	**ENT (ear, nose and throath) Group**
Tytti Willberg	Hospital District of Southwest Finland, Turku, Finland	tytti.willberg@tyks.fi	Clinical Groups	**ENT (ear, nose and throath) Group**
Elmo Saarentaus	Institute for Molecular Medicine Finland (FIMM), HiLIFE, University of Helsinki, Helsinki, Finland	elmo.saarentaus@helsinki.fi	Clinical Groups	**ENT (ear, nose and throath) Group**
Antti Aarnisalo	Hospital District of Helsinki and Uusimaa, Helsinki, Finland	antti.aarnisalo@hus.fi	Clinical Groups	**ENT (ear, nose and throath) Group**
Eveliina Salminen	Hospital District of Helsinki and Uusimaa, Helsinki, Finland	eveliina.e.salminen@hus.fi	Clinical Groups	**ENT (ear, nose and throath) Group**
Elisa Rahikkala	Northern Ostrobothnia Hospital District, Oulu, Finland	elisa.rahikkala@ppshp.fi	Clinical Groups	**ENT (ear, nose and throath) Group**
Johannes Kettunen	Northern Ostrobothnia Hospital District, Oulu, Finland	johannes.kettunen@oulu.fi	Clinical Groups	**ENT (ear, nose and throath) Group**
Kristiina Aittomäki	Department of Medical Genetics, Helsinki University Central Hospital, Helsinki, Finland	kristiina.aittomaki@helsinki.fi	Clinical Groups	**POI (premature ovarian failure) Group**
Fredrik Åberg	Transplantation and Liver Surgery Clinic, Helsinki University Hospital, Helsinki University, Helsinki, Finland	fredrik.aberg@helsinki.fi	Clinical Groups	**LiverScore Group**

Mitja Kurki	Institute for Molecular Medicine Finland (FIMM), HiLIFE, University of Helsinki, Helsinki, Finland; Broad Institute, Cambridge, MA, United States	mkurki@broadinstitute.org	FinnGen Analysis working grou	FinnGen Analysis working group
Samuli Ripatti	Institute for Molecular Medicine Finland (FIMM), HiLIFE, University of Helsinki, Helsinki, Finland	samuli.ripatti@helsinki.fi	FinnGen Analysis working grou	FinnGen Analysis working group
Mark Daly	Institute for Molecular Medicine, Finland (FIMM), HiLIFE, University of Helsinki, Helsinki, Finland; Broad Institute of MIT and Harvard; Massachusetts General Hospital	mark.daly@helsinki.fi	FinnGen Analysis working grou	FinnGen Analysis working group
Juha Karjalainen	Institute for Molecular Medicine Finland (FIMM), HiLIFE, University of Helsinki, Helsinki, Finland	juha.karjalainen@helsinki.fi	FinnGen Analysis working grou	FinnGen Analysis working group
Aki Havulinna	Institute for Molecular Medicine Finland (FIMM), HiLIFE, University of Helsinki, Helsinki, Finland	aki.havulinna@helsinki.fi	FinnGen Analysis working grou	FinnGen Analysis working group
Juha Mehtonen	Institute for Molecular Medicine Finland (FIMM), HiLIFE, University of Helsinki, Helsinki, Finland	juha.mehtonen@helsinki.fi	FinnGen Analysis working grou	FinnGen Analysis working group
Priit Palta	Institute for Molecular Medicine Finland (FIMM), HiLIFE, University of Helsinki, Helsinki, Finland	priit.palta@helsinki.fi	FinnGen Analysis working grou	FinnGen Analysis working group
Shabbeer Hassan	Institute for Molecular Medicine Finland (FIMM), HiLIFE, University of Helsinki, Helsinki, Finland	shabbeer.hassan@helsinki.fi	FinnGen Analysis working grou	FinnGen Analysis working group
Pietro Della Briotta Parolo	Institute for Molecular Medicine Finland (FIMM), HiLIFE, University of Helsinki, Helsinki, Finland	pietro.dellabriottaparolo@helsinki.fi	FinnGen Analysis working grou	FinnGen Analysis working group
Wei Zhou	Broad Institute, Cambridge, MA, United States	wzhou@broadinstitute.org	FinnGen Analysis working grou	FinnGen Analysis working group
Mutaamba Maasha	Broad Institute, Cambridge, MA, United States	mmaasha@broadinstitute.org	FinnGen Analysis working grou	FinnGen Analysis working group
Kumar Veerapen	Broad Institute, Cambridge, MA, United States	veerapen@broadinstitute.org	FinnGen Analysis working grou	FinnGen Analysis working group
Shabbeer Hassan	Institute for Molecular Medicine Finland (FIMM), HiLIFE, University of Helsinki, Helsinki, Finland	shabbeer.hassan@helsinki.fi	FinnGen Analysis working grou	FinnGen Analysis working group
Susanna Lemmelä	Institute for Molecular Medicine Finland (FIMM), HiLIFE, University of Helsinki, Helsinki, Finland	susanna.lemmela@helsinki.fi	FinnGen Analysis working grou	FinnGen Analysis working group
Manuel Rivas	University of Stanford, Stanford, CA, United States	mrivas@stanford.edu	FinnGen Analysis working grou	FinnGen Analysis working group
Mari E. Niemi	Institute for Molecular Medicine Finland (FIMM), HiLIFE, University of Helsinki, Helsinki, Finland	mari.e.niemi@helsinki.fi	FinnGen Analysis working grou	FinnGen Analysis working group
Aarno Palotie	Institute for Molecular Medicine Finland (FIMM), HiLIFE, University of Helsinki, Helsinki, Finland	aarno.palotie@helsinki.fi	FinnGen Analysis working grou	FinnGen Analysis working group
Aoxing Liu	Institute for Molecular Medicine Finland (FIMM), HiLIFE, University of Helsinki, Helsinki, Finland	aoxing.liu@helsinki.fi	FinnGen Analysis working grou	FinnGen Analysis working group
Arto Lehisto	Institute for Molecular Medicine Finland (FIMM), HiLIFE, University of Helsinki, Helsinki, Finland	arto.lehisto@helsinki.fi	FinnGen Analysis working grou	FinnGen Analysis working group
Andrea Ganna	Institute for Molecular Medicine Finland (FIMM), HiLIFE, University of Helsinki, Helsinki, Finland	aganna@broadinstitute.org	FinnGen Analysis working grou	FinnGen Analysis working group
Vincent Llorens	Institute for Molecular Medicine Finland (FIMM), HiLIFE, University of Helsinki, Helsinki, Finland	vincent.llorens@helsinki.fi	FinnGen Analysis working grou	FinnGen Analysis working group
Hannele Laivuori	Institute for Molecular Medicine Finland (FIMM), HiLIFE, University of Helsinki, Helsinki, Finland	hannele.laivuori@helsinki.fi	FinnGen Analysis working grou	FinnGen Analysis working group
Taru Tukiainen	Institute for Molecular Medicine Finland (FIMM), HiLIFE, University of Helsinki, Helsinki, Finland	taru.tukiainen@helsinki.fi	FinnGen Analysis working grou	FinnGen Analysis working group
Mary Pat Reeve	Institute for Molecular Medicine Finland (FIMM), HiLIFE, University of Helsinki, Helsinki, Finland	mary.reeve@helsinki.fi	FinnGen Analysis working grou	FinnGen Analysis working group
Henrike Heyne	Institute for Molecular Medicine Finland (FIMM), HiLIFE, University of Helsinki, Helsinki, Finland	hheyne@broadinstitute.org	FinnGen Analysis working grou	FinnGen Analysis working group
Nina Mars	Institute for Molecular Medicine Finland (FIMM), HiLIFE, University of Helsinki, Helsinki, Finland	nina.mars@helsinki.fi	FinnGen Analysis working grou	FinnGen Analysis working group
Joel Rämö	Institute for Molecular Medicine Finland (FIMM), HiLIFE, University of Helsinki, Helsinki, Finland	joel.ramo@helsinki.fi	FinnGen Analysis working grou	FinnGen Analysis working group
Elmo Saarentaus	Institute for Molecular Medicine Finland (FIMM), HiLIFE, University of Helsinki, Helsinki, Finland	elmo.saarentaus@helsinki.fi	FinnGen Analysis working grou	FinnGen Analysis working group
Hanna Ollila	Institute for Molecular Medicine Finland (FIMM), HiLIFE, University of Helsinki, Helsinki, Finland	hanna.m.ollila@helsinki.fi	FinnGen Analysis working grou	FinnGen Analysis working group
Rodos Rodosthenous	Institute for Molecular Medicine Finland (FIMM), HiLIFE, University of Helsinki, Helsinki, Finland	rodos.rodosthenous@helsinki.fi	FinnGen Analysis working grou	FinnGen Analysis working group
Satu Strausz	Institute for Molecular Medicine Finland (FIMM), HiLIFE, University of Helsinki, Helsinki, Finland	satu.strausz@helsinki.fi	FinnGen Analysis working grou	FinnGen Analysis working group
Tuula Palotie	University of Helsinki and Hospital District of Helsinki and Uusimaa, Helsinki, Finland	tuula.palotie@helsinki.fi	FinnGen Analysis working grou	FinnGen Analysis working group
Kimmo Palin	University of Helsinki, Helsinki, Finland	kimmo.palin@helsinki.fi	FinnGen Analysis working grou	FinnGen Analysis working group

Javier Garcia-Tabuenca	University of Tampere, Tampere, Finland	javier.graciatabuenca@tuni.fi	FinnGen Analysis working grou	FinnGen Analysis working group
Harri Siirtola	University of Tampere, Tampere, Finland	harri.siirtola@tuni.fi	FinnGen Analysis working grou	FinnGen Analysis working group
Tuomo Kiiskinen	Institute for Molecular Medicine Finland (FIMM), HiLIFE, University of Helsinki, Helsinki, Finland	tuomo.kiiskinen@helsinki.fi	FinnGen Analysis working grou	FinnGen Analysis working group
Jiwoo Lee	Institute for Molecular Medicine Finland (FIMM), HiLIFE, University of Helsinki, Helsinki, Finland; Broad Institute, Cambridge, MA, United States	jiwoo.lee@helsinki.fi	FinnGen Analysis working grou	FinnGen Analysis working group
Kristin Tsuo	Institute for Molecular Medicine Finland (FIMM), HiLIFE, University of Helsinki, Helsinki, Finland; Broad Institute, Cambridge, MA, United States	kristintsuo@fas.harvard.edu	FinnGen Analysis working grou	FinnGen Analysis working group
Amanda Elliott	Institute for Molecular Medicine Finland (FIMM), HiLIFE, University of Helsinki, Helsinki, Finland; Broad Institute, Cambridge, MA, USA and Massachusetts General Hospital, Boston,	aelliott@broadinstitute.org	FinnGen Analysis working grou	FinnGen Analysis working group
Kati Kristiansson	THL Biobank / Finnish Institute for Health and Welfare (THL), Helsinki, Finland	kati.kristiansson@thl.fi	FinnGen Analysis working grou	FinnGen Analysis working group
Mikko Arvas	Finnish Red Cross Blood Service / Finnish Hematology Registry and Clinical Biobank, Helsinki, Finland	mikko.arvas@veripalvelu.fi	FinnGen Analysis working grou	FinnGen Analysis working group
Kati Hyvärinen	Finnish Red Cross Blood Service, Helsinki, Finland	kati.hyvarinen@veripalvelu.fi	FinnGen Analysis working grou	FinnGen Analysis working group
Jarmo Ritari	Finnish Red Cross Blood Service, Helsinki, Finland	jarmo.ritari@veripalvelu.fi	FinnGen Analysis working grou	FinnGen Analysis working group
Olli Carpén	Helsinki Biobank / Helsinki University and Hospital District of Helsinki and Uusimaa, Helsinki	olli.carpen@helsinki.fi	FinnGen Analysis working grou	FinnGen Analysis working group
Johannes Kettunen	Northern Finland Biobank Borealis / University of Oulu / Northern Ostrobothnia Hospital District, Oulu, Finland	johannes.kettunen@oulu.fi	FinnGen Analysis working grou	FinnGen Analysis working group
Katri Pylkäs	University of Oulu, Oulu, Finland	katri.pylkas@oulu.fi	FinnGen Analysis working grou	FinnGen Analysis working group
Eeva Sliz	University of Oulu, Oulu, Finland	eeva.sliz@oulu.fi	FinnGen Analysis working grou	FinnGen Analysis working group
Minna Karjalainen	University of Oulu, Oulu, Finland	minna.k.karjalainen@oulu.fi	FinnGen Analysis working grou	FinnGen Analysis working group
Tuomo Mantere	Northern Finland Biobank Borealis / University of Oulu / Northern Ostrobothnia Hospital District, Oulu, Finland	tuomo.mantere@oulu.fi	FinnGen Analysis working grou	FinnGen Analysis working group
Eeva Kangasniemi	Finnish Clinical Biobank Tampere / University of Tampere / Pirkanmaa Hospital District, Tampere, Finland	eeva.kangasniemi@pshp.fi	FinnGen Analysis working grou	FinnGen Analysis working group
Sami Heikkinen	University of Eastern Finland, Kuopio, Finland	sami.heikkinen@uef.fi	FinnGen Analysis working grou	FinnGen Analysis working group
Arto Mannermaa	Biobank of Eastern Finland / University of Eastern Finland / Northern Savo Hospital District, Kuopio, Finland	arto.mannermaa@uef.fi	FinnGen Analysis working grou	FinnGen Analysis working group
Eija Laakkonen	University of Jyväskylä, Jyväskylä, Finland	eija.k.laakkonen@jyu.fi	FinnGen Analysis working grou	FinnGen Analysis working group
Nina Pitkänen	Auria Biobank / University of Turku / Hospital District of Southwest Finland, Turku, Finland	Niina.Pitkanen@tyks.fi	FinnGen Analysis working grou	FinnGen Analysis working group
Samuel Lessard	Translational Sciences, Sanofi R&D, Framingham, MA, USA	samuel.lessard@sanofi.com	FinnGen Analysis working grou	FinnGen Analysis working group
Clément Chatelain	Translational Sciences, Sanofi R&D, Framingham, MA, USA	clement.chatelain@sanofi.com	FinnGen Analysis working grou	FinnGen Analysis working group
Perttu Terho	Auria Biobank / University of Turku / Hospital District of Southwest Finland, Turku, Finland	perttu.terho@tyks.fi	Biobank directors	Biobank directors
Sirpa Soini	THL Biobank / Finnish Institute for Health and Welfare (THL), Helsinki, Finland	sirpa.soini@thl.fi	Biobank directors	Biobank directors
Jukka Partanen	Finnish Red Cross Blood Service / Finnish Hematology Registry and Clinical Biobank, Helsinki, Finland	jukka.partanen@veripalvelu.fi	Biobank directors	Biobank directors

Eero Punkka	Helsinki Biobank / Helsinki University and Hospital District of Helsinki and Uusimaa, Helsinki	eero.punkka@hus.fi	**Biobank directors**	**Biobank directors**
Raisa Serpi	Northern Finland Biobank Borealis / University of Oulu / Northern Ostrobothnia Hospital District, Oulu, Finland	raisa.serpi@ppshp.fi	**Biobank directors**	**Biobank directors**
Sanna Siltanen	Finnish Clinical Biobank Tampere / University of Tampere / Pirkanmaa Hospital District, Tampere, Finland	sanna.siltanen@pshp.fi	**Biobank directors**	**Biobank directors**
Veli-Matti Kosma	Biobank of Eastern Finland / University of Eastern Finland / Northern Savo Hospital District, Kuopio, Finland	veli-matti.kosma@uef.fi	**Biobank directors**	**Biobank directors**
Teijo Kuopio	Central Finland Biobank / University of Jyväskylä / Central Finland Health Care District, Jyväskylä, Finland	teijo.kuopio@ksshp.fi	**Biobank directors**	**Biobank directors**
Anu Jalanko	Institute for Molecular Medicine Finland (FIMM), HiLIFE, University of Helsinki, Helsinki, Finland	anu.jalanko@helsinki.fi	**FinnGen Teams**	**Administration**
Huei-Yi Shen	Institute for Molecular Medicine Finland (FIMM), HiLIFE, University of Helsinki, Helsinki, Finland	huei-yi.shen@helsinki.fi	**FinnGen Teams**	**Administration**
Risto Kajanne	Institute for Molecular Medicine Finland (FIMM), HiLIFE, University of Helsinki, Helsinki, Finland	risto.kajanne@helsinki.fi	**FinnGen Teams**	**Administration**
Mervi Aavikko	Institute for Molecular Medicine Finland (FIMM), HiLIFE, University of Helsinki, Helsinki, Finland	mervi.aavikko@helsinki.fi	**FinnGen Teams**	**Administration**
Mitja Kurki	Institute for Molecular Medicine Finland (FIMM), HiLIFE, University of Helsinki, Helsinki, Finland; Broad Institute, Cambridge, MA, United States	mkurki@broadinstitute.org	**FinnGen Teams**	**Analysis**
Juha Karjalainen	Institute for Molecular Medicine Finland (FIMM), HiLIFE, University of Helsinki, Helsinki, Finland	juha.karjalainen@helsinki.fi	**FinnGen Teams**	**Analysis**
Pietro Della Briotta Parolo	Institute for Molecular Medicine Finland (FIMM), HiLIFE, University of Helsinki, Helsinki, Finland	pietro.dellabriottaparolo@helsinki.fi	**FinnGen Teams**	**Analysis**
Arto Lehisto	Institute for Molecular Medicine Finland (FIMM), HiLIFE, University of Helsinki, Helsinki, Finland	arto.lehisto@helsinki.fi	**FinnGen Teams**	**Analysis**
Juha Mehtonen	Institute for Molecular Medicine Finland (FIMM), HiLIFE, University of Helsinki, Helsinki, Finland	juha.mehtonen@helsinki.fi	**FinnGen Teams**	**Analysis**
Wei Zhou	Broad Institute, Cambridge, MA, United States	wzhou@broadinstitute.org	**FinnGen Teams**	**Analysis**
Masahiro Kanai	Broad Institute, Cambridge, MA, United States	mkanai@broadinstitute.org	**FinnGen Teams**	**Analysis**
Mutaamba Maasha	Broad Institute, Cambridge, MA, United States	mmaasha@broadinstitute.org	**FinnGen Teams**	**Analysis**
Kumar Veerapen	Broad Institute, Cambridge, MA, United States	veerapen@broadinstitute.org	**FinnGen Teams**	**Analysis**
Hannele Laivuori	Institute for Molecular Medicine Finland (FIMM), HiLIFE, University of Helsinki, Helsinki, Finland	hannele.laivuori@helsinki.fi	**FinnGen Teams**	**Clinical Endpoint Development**
Aki Havulinna	Institute for Molecular Medicine Finland (FIMM), HiLIFE, University of Helsinki, Helsinki, Finland	aki.havulinna@helsinki.fi	**FinnGen Teams**	**Clinical Endpoint Development**
Susanna Lemmelä	Institute for Molecular Medicine Finland (FIMM), HiLIFE, University of Helsinki, Helsinki, Finland	susanna.lemmela@helsinki.fi	**FinnGen Teams**	**Clinical Endpoint Development**
Tuomo Kiiskinen	Institute for Molecular Medicine Finland (FIMM), HiLIFE, University of Helsinki, Helsinki, Finland	tuomo.kiiskinen@helsinki.fi	**FinnGen Teams**	**Clinical Endpoint Development**
L. Elisa Lahtela	Institute for Molecular Medicine Finland (FIMM), HiLIFE, University of Helsinki, Helsinki, Finland	laura.lahtela@helsinki.fi	**FinnGen Teams**	**Clinical Endpoint Development**
Mari Kaunisto	Institute for Molecular Medicine Finland (FIMM), HiLIFE, University of Helsinki, Helsinki, Finland	mari.kaunisto@helsinki.fi	**FinnGen Teams**	**Communication**
Elina Kilpeläinen	Institute for Molecular Medicine Finland (FIMM), HiLIFE, University of Helsinki, Helsinki, Finland	elina.kilpelainen@helsinki.fi	**FinnGen Teams**	**E-Science**
Timo P. Sipilä	Institute for Molecular Medicine Finland (FIMM), HiLIFE, University of Helsinki, Helsinki, Finland	timo.p.sipila@helsinki.fi	**FinnGen Teams**	**E-Science**
Oluwaseun Alexander Dada	Institute for Molecular Medicine Finland (FIMM), HiLIFE, University of Helsinki, Helsinki, Finland	alexander.dada@helsinki.fi	**FinnGen Teams**	**E-Science**
Awaisa Ghazal	Institute for Molecular Medicine Finland (FIMM), HiLIFE, University of Helsinki, Helsinki, Finland	awaisa.ghazal@helsinki.fi	**FinnGen Teams**	**E-Science**
Anastasia Kytölä	Institute for Molecular Medicine Finland (FIMM), HiLIFE, University of Helsinki, Helsinki, Finland	anastasia.shcherban@helsinki.fi	**FinnGen Teams**	**E-Science**
Rigbe Weldatsadik	Institute for Molecular Medicine Finland (FIMM), HiLIFE, University of Helsinki, Helsinki, Finland	rigbe.weldatsadik@helsinki.fi	**FinnGen Teams**	**E-Science**

Kati Donner	Institute for Molecular Medicine Finland (FIMM), HiLIFE, University of Helsinki, Helsinki, Finland	kati.donner@helsinki.fi	**FinnGen Teams**	**Genotyping**
Timo P. Sipilä	Institute for Molecular Medicine Finland (FIMM), HiLIFE, University of Helsinki, Helsinki, Finland	timo.p.sipila@helsinki.fi	**FinnGen Teams**	**Genotyping**
Anu Loukola	Helsinki Biobank / Helsinki University and Hospital District of Helsinki and Uusimaa, Helsinki	anu.loukola@hus.fi	**FinnGen Teams**	**Sample Collection Coordination**
Päivi Laiho	THL Biobank / Finnish Institute for Health and Welfare (THL), Helsinki, Finland	paivi.laiho@thl.fi	**FinnGen Teams**	**Sample Logistics**
Tuuli Sistonen	THL Biobank / Finnish Institute for Health and Welfare (THL), Helsinki, Finland	tuuli.sistonen@thl.fi	**FinnGen Teams**	**Sample Logistics**
Essi Kaiharju	THL Biobank / Finnish Institute for Health and Welfare (THL), Helsinki, Finland	essi.kaiharju@thl.fi	**FinnGen Teams**	**Sample Logistics**
Markku Laukkanen	THL Biobank / Finnish Institute for Health and Welfare (THL), Helsinki, Finland	markku.laukkanen@thl.fi	**FinnGen Teams**	**Sample Logistics**
Elina Järvensivu	THL Biobank / Finnish Institute for Health and Welfare (THL), Helsinki, Finland	elina.jarvensivu@thl.fi	**FinnGen Teams**	**Sample Logistics**
Sini Lähteenmäki	THL Biobank / Finnish Institute for Health and Welfare (THL), Helsinki, Finland	sini.lahteenmaki@thl.fi	**FinnGen Teams**	**Sample Logistics**
Lotta Männikkö	THL Biobank / Finnish Institute for Health and Welfare (THL), Helsinki, Finland	lotta.mannikko@thl.fi	**FinnGen Teams**	**Sample Logistics**
Regis Wong	THL Biobank / Finnish Institute for Health and Welfare (THL), Helsinki, Finland	regis.wong@thl.fi	**FinnGen Teams**	**Sample Logistics**
Auli Toivola	THL Biobank / Finnish Institute for Health and Welfare (THL), Helsinki, Finland	auli.toivola@thl.fi	**FinnGen Teams**	**Sample Logistics**
Minna Brunfeldt	THL Biobank / Finnish Institute for Health and Welfare (THL), Helsinki, Finland	minna.brunfeldt@thl.fi	**FinnGen Teams**	**Registry Data Operations**
Hannele Mattsson	THL Biobank / Finnish Institute for Health and Welfare (THL), Helsinki, Finland	hannele.mattsson@thl.fi	**FinnGen Teams**	**Registry Data Operations**
Kati Kristiansson	THL Biobank / Finnish Institute for Health and Welfare (THL), Helsinki, Finland	kati.kristiansson@thl.fi	**FinnGen Teams**	**Registry Data Operations**
Susanna Lemmelä	Institute for Molecular Medicine Finland (FIMM), HiLIFE, University of Helsinki, Helsinki, Finland	susanna.lemmela@helsinki.fi	**FinnGen Teams**	**Registry Data Operations**
Sami Koskelainen	THL Biobank / Finnish Institute for Health and Welfare (THL), Helsinki, Finland	sami.koskelainen@thl.fi	**FinnGen Teams**	**Registry Data Operations**
Tero Hiekkalinna	THL Biobank / Finnish Institute for Health and Welfare (THL), Helsinki, Finland	tero.hiekkalinna@helsinki.fi	**FinnGen Teams**	**Registry Data Operations**
Teemu Paajanen	THL Biobank / Finnish Institute for Health and Welfare (THL), Helsinki, Finland	teemu.paajanen@thl.fi	**FinnGen Teams**	**Registry Data Operations**
Priit Palta	Institute for Molecular Medicine Finland (FIMM), HiLIFE, University of Helsinki, Helsinki, Finland	priit.palta@helsinki.fi	**FinnGen Teams**	**Sequencing Informatics**
Kalle Pärn	Institute for Molecular Medicine Finland (FIMM), HiLIFE, University of Helsinki, Helsinki, Finland	kalle.parn@helsinki.fi	**FinnGen Teams**	**Sequencing Informatics**
Mart Kals	Institute for Molecular Medicine Finland (FIMM), HiLIFE, University of Helsinki, Helsinki, Finland	mart.kals@helsinki.fi	**FinnGen Teams**	**Sequencing Informatics**
Shuang Luo	Institute for Molecular Medicine Finland (FIMM), HiLIFE, University of Helsinki, Helsinki, Finland	shuang.luo@helsinki.fi	**FinnGen Teams**	**Sequencing Informatics**
Vishal Sinha	Institute for Molecular Medicine Finland (FIMM), HiLIFE, University of Helsinki, Helsinki, Finland	vishal.sinha@helsinki.fi	**FinnGen Teams**	**Sequencing Informatics**
Tarja Laitinen	Pirkanmaa Hospital District, Tampere, Finland	tarja.laitinen@pshp.fi	**FinnGen Teams**	**Trajectory**
Mary Pat Reeve	Institute for Molecular Medicine Finland (FIMM), HiLIFE, University of Helsinki, Helsinki, Finland	mary.reeve@helsinki.fi	**FinnGen Teams**	**Trajectory**
Marianna Niemi	University of Tampere, Tampere, Finland	marianna.niemi@tuni.fi	**FinnGen Teams**	**Trajectory**
Kumar Veerapen	Broad Institute, Cambridge, MA, United States	veerapen@broadinstitute.org	**FinnGen Teams**	**Trajectory**
Harri Siirtola	University of Tampere, Tampere, Finland	harri.siirtola@tuni.fi	**FinnGen Teams**	**Trajectory**
Javier Gracia-Tabuenca	University of Tampere, Tampere, Finland	javier.graciatabuenca@tuni.fi	**FinnGen Teams**	**Trajectory**
Mika Helminen	University of Tampere, Tampere, Finland	mika.helminen@tuni.fi	**FinnGen Teams**	**Trajectory**
Tiina Luukkaala	University of Tampere, Tampere, Finland	tiina.luukkaala@tuni.fi	**FinnGen Teams**	**Trajectory**
Iida Vähätalo	University of Tampere, Tampere, Finland	iida.vahatalo@epshp.fi	**FinnGen Teams**	**Trajectory**
Jyrki Pitkänen	Institute for Molecular Medicine Finland (FIMM), HiLIFE, University of Helsinki, Helsinki, Finland	jyrki.pitkanen@helsinki.fi	**FinnGen Teams**	**Data protection officer**
Marco Hautalahti	Finnish Biobank Cooperative - FINBB	marco.hautalahti@finbb.fi	**FinnGen Teams**	**FINBB - Finnish biobank cooperative**

Johanna Mäkelä	Finnish Biobank Cooperative - FINBB	johanna.makela@finbb.fi	**FinnGen Teams**	**FINBB - Finnish biobank cooperative**
Sarah Smith	Finnish Biobank Cooperative - FINBB	sarah.smith@finbb.fi	**FinnGen Teams**	**FINBB - Finnish biobank cooperative**
Tom Southerington	Finnish Biobank Cooperative - FINBB	tom.southerington@finbb.fi	**FinnGen Teams**	**FINBB - Finnish biobank cooperative**
