# Supplementary material for: Immune system-wide Mendelian randomization and triangulation analyses support autoimmunity as a modifiable component in dementia-causing diseases
Source: Nat Aging. 2022 Oct 14;2(10):956–72. doi: 10.1038/s43587-022-00293-x (PMC10154235; doi:10.1038/s43587-022-00293-x)
Supplement: Supplementary file 1 [file 43587_2022_293_MOESM2_ESM.pdf]

Corresponding author(s): Joni V. Lindbohm

Last updated by author(s): Aug 31, 2022

Reporting Summary

Nature Research wishes to improve the reproducibility of the work that we publish. This form provides structure for consistency and transparency in reporting. For further information on Nature Research policies, see our Editorial Policies and the Editorial Policy Checklist.

Statistics

For all statistical analyses, confirm that the following items are present in the figure legend, table legend, main text, or Methods section.

n/a	Confirmed	
☐	☒	The exact sample size (*n*) for each experimental group/condition, given as a discrete number and unit of measurement
☐	☒	A statement on whether measurements were taken from distinct samples or whether the same sample was measured repeatedly
☐	☒	The statistical test(s) used AND whether they are one- or two-sided<br>*Only common tests should be described solely by name; describe more complex techniques in the Methods section.*
☐	☒	A description of all covariates tested
☐	☒	A description of any assumptions or corrections, such as tests of normality and adjustment for multiple comparisons
☐	☒	A full description of the statistical parameters including central tendency (e.g. means) or other basic estimates (e.g. regression coefficient) AND variation (e.g. standard deviation) or associated estimates of uncertainty (e.g. confidence intervals)
☐	☒	For null hypothesis testing, the test statistic (e.g. F, t, r) with confidence intervals, effect sizes, degrees of freedom and P value noted<br>*Give P values as exact values whenever suitable.*
☒	☐	For Bayesian analysis, information on the choice of priors and Markov chain Monte Carlo settings
☒	☐	For hierarchical and complex designs, identification of the appropriate level for tests and full reporting of outcomes
☐	☒	Estimates of effect sizes (e.g. Cohen's *d*, Pearson's *r*), indicating how they were calculated

Our web collection on statistics for biologists contains articles on many of the points above.

Software and code

Policy information about availability of computer code

Data collection	No software was used for data collection.
Data analysis	We used following softwares for imputation, handling the genetic and phenotypic data:<br><br>Cromwell 61<br>PLINK v2.00aLM3<br>BCFtools 1.7 and 1.9<br>Eagle 2.3.5<br>Beagle 4.1 (08Jun17.d8b)<br>The full genotyping and imputation protocol for FinnGen is described at dx.doi.org/10.17504/protocols.io.nmndc5e<br>For data transformations, visualization and plotting of the results, we used R (3.6.0, 4.1.0, 4.1.2), including packages:<br>data.table 1.14.2<br>dplyr 1.0.7<br>tidyr 1.1.4<br>survival 3.2.13<br>survminer 0.4.9<br>ggplot2 3.3.5<br>plyr 1.8.6<br>cluster 2.1.2<br>lubridate 1.8.0<br>stats 4.1.1<br>readxl 1.3.1<br>scales 1.1.1

tidyverse 1.3.1
Hmisc 4.6.0
devtools 2.4.2
TwoSampleMR 0.5.6
MRInstruments 0.3.2
metafor 3.0.2.

Other software include: ClueGO version 2.5.8, Cytoscape version 3.7.2.

ggplot2, survminer, TwoSampleMR, MRInstruments, and ipw

For manuscripts utilizing custom algorithms or software that are central to the research but not yet described in published literature, software must be made available to editors and reviewers. We strongly encourage code deposition in a community repository (e.g. GitHub). See the Nature Research guidelines for submitting code & software for further information.

Data

Policy information about availability of data

All manuscripts must include a data availability statement. This statement should provide the following information, where applicable:

- Accession codes, unique identifiers, or web links for publicly available datasets
- A list of figures that have associated raw data
- A description of any restrictions on data availability

The updated data availability statement is here as it is described in the manuscript:
Data availability
This study used publicly available data at https://www.mrbase.org/, https://www.uniprot.org/, http://cpdb.molgen.mpg.de/, https://www.genome.jp/kegg/, and https://www.opentargets.org/. Data used in Mendelian Randomization are deposited to Zenodo: https://zenodo.org/deposit/7042008. Data, protocols, and other metadata of the Whitehall II and FinnGen studies are available according to the data sharing policies of these studies. Pre-existing data access policy for Whitehall II specify that research data requests can be submitted to study steering committee; these will be promptly reviewed for confidentiality or intellectual property restrictions and will not unreasonably be refused. Detailed information on data sharing can be found here: https://www.ucl.ac.uk/epidemiology-health-care/research/epidemiology-and-public-health/research/whitehall-ii/data-sharing Individual-level patient or protein data may further be restricted by consent, confidentiality, or privacy laws/considerations. The FinnGen data can be accessed through Finnish Biobanks' FinBB portal (www.finbb.fi). FinnGen summary statistics are freely available at https://www.finngen.fi/en/access_results, with results for new data freezes updated every 6 months.

Field-specific reporting

Please select the one below that is the best fit for your research. If you are not sure, read the appropriate sections before making your selection.

☒ Life sciences ☐ Behavioural & social sciences ☐ Ecological, evolutionary & environmental sciences

For a reference copy of the document with all sections, see nature.com/documents/nr-reporting-summary-flat.pdf

Life sciences study design

All studies must disclose on these points even when the disclosure is negative.

Sample size	We used all available data on individuals 18 or older at the end of follow-up. The study is based on FinnGen Data Freeze 8 with 339,233 individuals. In the Whitehall II study we used the 6,545 individuals with plasma proteomic data available.
Data exclusions	Inverse-probability-weighted analyses in FinnGen used only 117,773 participants aged above 45 and with no medication studied at baseline. This was done to ensure that all participants were old enough to develop dementia over the follow-up and to simulate randomized control trial design using observational data.
Replication	The study did not include direct replication with same methods in different cohort. Instead it used six complimentary approaches to study the same research question and all provided consistent results.
Randomization	The study used inverse-probability-weighted analyses to simulate randomized control trial design using observational data. This is an observational study.
Blinding	No blinding was relevant for this observational study.

Reporting for specific materials, systems and methods

We require information from authors about some types of materials, experimental systems and methods used in many studies. Here, indicate whether each material, system or method listed is relevant to your study. If you are not sure if a list item applies to your research, read the appropriate section before selecting a response.

Materials & experimental systems

n/a	Involved in the study
☒	☐ Antibodies
☒	☐ Eukaryotic cell lines
☒	☐ Palaeontology and archaeology
☒	☐ Animals and other organisms
☐	☒ Human research participants
☒	☐ Clinical data
☒	☐ Dual use research of concern

Methods

n/a	Involved in the study
☒	☐ ChIP-seq
☒	☐ Flow cytometry
☒	☐ MRI-based neuroimaging

Human research participants

Policy information about studies involving human research participants

Population characteristics

This study included participants from different study cohorts, all of European ancestry. FinnGen Data Freeze 8 comprises 339,233 individuals (56.3% women; mean age 59.8 at the end of follow-up in 2019, with standard deviation, s.d. 17.3 years).

In Whitehall II the mean age was 56.1 (SD 5.9) and 73.0% were men and 92.3% of European ancestry.

Recruitment

Random sample of subjects from Finnish population-based and clinical biobanks. Participation was voluntary. A proportion of FinnGen was ascertained through hospital biobanks and disease-based collections.

In 1985-1988, all civil servants aged 35 to 55 years based in 20 departments in London, UK, were invited to participate in the Whitehall II cohort study, and 73% (n=10 308) agreed. Blood samples for proteomic analyses were collected from a random subsample of 6,545 dementia-free individuals in 1997-99 which was the baseline for this study.
.

Ethics oversight

Patients and control subjects in FinnGen provided informed consent for biobank research, based on the Finnish Biobank Act. Alternatively, separate research cohorts, collected prior the Finnish Biobank Act came into effect (in September 2013) and start of FinnGen (August 2017), were collected based on study-specific consents and later transferred to the Finnish biobanks after approval by Fimea (Finnish Medicines Agency), the National Supervisory Authority for Welfare and Health. Recruitment protocols followed the biobank protocols approved by Fimea. The Coordinating Ethics Committee of the Hospital District of Helsinki and Uusimaa (HUS) statement number for the FinnGen study is Nr HUS/990/2017.

The FinnGen study is approved by Finnish Institute for Health and Welfare (permit numbers: THL/2031/6.02.00/2017, THL/1101/5.05.00/2017, THL/341/6.02.00/2018, THL/2222/6.02.00/ 2018, THL/283/6.02.00/2019, THL/1721/5.05.00/2019, THL/1524/5.05.00/2020, and THL/2364/ 14.02/2020), Digital and population data service agency (permit numbers: VRK43431/2017-3, VRK/6909/2018-3, VRK/4415/2019-3), the Social Insurance Institution (permit numbers: KELA 58/522/2017, KELA 131/522/2018, KELA 70/522/2019, KELA 98/522/2019, KELA 138/522/2019, KELA 2/522/2020, KELA 16/522/2020, Findata THL/2364/14.02/2020 and Statistics Finland (permit numbers: TK-53-1041-17 and TK/143/07.03.00/2020 (earlier TK-53-90-20).

The Biobank Access Decisions for FinnGen samples and data utilized in FinnGen Data Freeze 7 include: THL Biobank BB2017_55, BB2017_111, BB2018_19, BB_2018_34, BB_2018_67, BB2018_71, BB2019_7, BB2019_8, BB2019_26, BB2020_1, Finnish Red Cross Blood Service Biobank 7.12.2017, Helsinki Biobank HUS/359/2017, Auria Biobank AB17-5154 and amendment #1 (August 17 2020), Biobank Borealis of Northern Finland_2017_1013, Biobank of Eastern Finland 1186/2018 and amendment 22 § /2020, Finnish Clinical Biobank Tampere MH0004 and amendments (21.02.2020 & 06.10.2020), Central Finland Biobank 1-2017, and Terveystalo Biobank STB 2018001.

In Whitehall II study, participants provided written informed consent for inclusion at each examination. Research ethics approval was granted by the University College London Hospital Committee on the Ethics of Human Research.

Note that full information on the approval of the study protocol must also be provided in the manuscript.
